# Designing the Next Generation of Biomaterials through Nanoengineering

**DOI:** 10.1002/adma.202501761

**Published:** 2025-07-11

**Authors:** Ryan Davis, Ishaan Duggal, Nicholas A. Peppas, Akhilesh K. Gaharwar

**Affiliations:** ^1^ Department of Biomedical Engineering College of Engineering Texas A&M University College Station TX 77843 USA; ^2^ Institute for Biomaterials, Drug Delivery, and Regenerative Medicine The University of Texas at Austin Austin TX 78712 USA; ^3^ Division of Molecular Pharmaceutics and Drug Delivery, College of Pharmacy The University of Texas at Austin Austin TX 78712 USA; ^4^ McKetta Department of Chemical Engineering The University of Texas at Austin Austin TX 78712 USA; ^5^ Department of Biomedical Engineering The University of Texas at Austin Austin TX 78712 USA; ^6^ Department of Surgery and Perioperative Care Dell Medical School University of Texas at Austin Austin TX 78712 USA; ^7^ Department of Pediatrics Dell Medical School University of Texas at Austin Austin TX 78712 USA; ^8^ Department of Material Science and Engineering College of Engineering Texas A&M University College Station TX 77843 USA; ^9^ Interdisciplinary Program in Genetics Texas A&M University College Station TX 77843 USA; ^10^ Center for Remote Health Technologies and Systems Texas A&M University College Station TX 77843 USA

**Keywords:** bioimaging, biomaterials, drug delivery, nanoengineering, regenerative medicine

## Abstract

Recent advances in biomaterials science have applied nanoengineering to develop biomaterials with superior properties and tailored functionalities. These unique attributes are achieved due to the ability of nanoengineering to provide precise control over material interactions with living systems at the molecular scale. Here, key nanotechnologies employed to develop the next generation of biomaterials are critically evaluated. A diverse range of nanomaterials, differing in base materials, shapes, sizes, or surface properties can be integrated into various fabrication processes to develop these advanced biomaterials. Further investigation is required into properties such as surface energy, defects, porosity, and crystallinity, as these critically influence the physical, chemical, and biological characteristics of nanoengineered materials. Consequently, we explore diverse biomedical applications of nanoengineered biomaterials, including regenerative medicine, biomolecular delivery, additive manufacturing, immune engineering, cancer therapeutics, bioimaging, biosensing, antimicrobial devices, and tissue adhesives. Additionally, their current limitations are analyzed and emerging strategies for designing the next generation of nanoengineered biomaterials are highlighted.

## Graphical abstract

Nanoengineering enables precise control over biomaterial interactions with living systems by tuning surface energy, defects, porosity, and crystallinity. This review highlights how these nanoscale design parameters drive advances in regenerative medicine, drug delivery, bioprinting, biosensing, and bioimaging, while outlining key translational challenges and future directions.

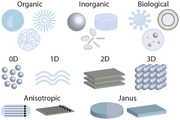

## 1 Introduction

Biomaterials are well‐defined materials designed to take a form that can promote the course of any therapeutic or diagnostic procedure,^[^ ^1^ ^]^ through interactions with living systems. These interactions are primarily governed by chemical structure and are influenced by material properties such as surface roughness or mechanical stiffness. In conventional biomaterials, control over these properties, and ultimately these interactions, is limited to the macroscopic scale. Recent advances in biomaterial fabrication have leveraged nanoengineering to precisely tune material interactions with cells, proteins, microbes, and other extracellular matrix components. Nanoengineering biomaterials lead to improved cellular functions, targeted delivery of small molecule therapeutics and biologics, and controlled response to stimuli.

The concept of nanomaterials originated in 1959 when Richard Feynman proposed the manipulation of individual atoms to achieve complex structures on the nanoscale size range.^[^ ^2^ ^]^ Since then, the field of nanotechnology has evolved to innovations across multiple disciplines such as electronics, materials science, and biomedicine. Nanoengineering has significantly advanced the design of biomaterials for both therapeutic and diagnostic applications. This approach is grounded in the principle that the source, structure, and geometry of nanoscale building blocks dictate the properties of the bulk material. For instance, silicate nanoplatelets with charge anisotropy form hydrogels via reversible electrostatic interactions, imparting shear‐thinning and self‐healing characteristics.^[^ ^3^ ^]^ Similarly, porous bone scaffolds can be engineered by combining cell‐adhesive nanofibers with osteoinductive nanoparticles to mimic native bone architecture and enhance regeneration.^[^ ^4^ ^]^ The composition, dimensionality, and symmetry of nanoscale components can be precisely tuned to modulate biological responses and promote integration with host tissues.

Beyond material design, fabrication strategies such as biomimicry, self‐assembly, layer‐by‐layer deposition, and electrospinning offer precise spatial control over nanoengineered architectures. Techniques like flow‐induced nanofibril alignment enable the fabrication of biomaterials that replicate the hierarchical structures of wood, tendon, and shell, resulting in materials with tunable and superior mechanical properties.^[^ ^5^ ^]^ Dip‐pen nanolithography allows for high‐resolution patterning, advancing the development of ultrasensitive biosensors.^[^ ^6^ ^]^ These nanofabrication approaches support the creation of complex biomaterials with enhanced functionalities and responsiveness, tailored to interact with biological systems. At the nanoscale, material behavior is dominated by surface properties rather than bulk characteristics. Surface functionalization—such as introducing oxygen, nitrogen, or fluorine groups on graphene—modulates surface energy and influences thermal and electrical transport properties.^[^ ^7^ ^]^ In addition, emerging studies emphasize the critical roles of nanoscale defects, pore architecture, and crystallinity in tuning biomaterial performance. These parameters impact catalytic reactivity,^[^ ^8^ ^]^ mechanical strength and stress distribution,^[^ ^9^ ^]^ and cell‐material interactions,^[^ ^10^ ^]^ underscoring their importance in the rational design of nanoengineered biomaterials.

Nanoengineered biomaterials have broad applicability across regenerative medicine, drug delivery, cancer therapeutics, additive manufacturing, bioimaging, biosensing, antimicrobial coatings, and tissue adhesives. Through biomimetic surface patterning, modulation of enzymatic activity, and control of cellular functions, nanoengineering enhances the biophysical and biochemical cues that support tissue regeneration. The architectural precision and multifunctionality of nanomaterials enable tuning of drug release kinetics, increased therapeutic loading, and site‐specific delivery. In additive manufacturing, the incorporation of nanomaterials improves structural resolution^[^ ^11^ ^]^ and introduces dynamic responsiveness to internal^[^ ^12^ ^]^ or external stimuli.^[^ ^13^ ^]^ Immunoactive nanomaterials can interface with the host immune system to modulate physiological responses, while others function as biosensors or fluorescent imaging agents for real‐time biomarker monitoring. Antimicrobial nanomaterials are being developed to reduce implant‐associated infections, and nanoengineered tissue adhesives demonstrate improved adhesion strength and responsiveness to environmental triggers.

Despite rapid advancements in research regarding the application of nanoengineered biomaterials,^[^ ^14^ ^]^ the clinical translation of these strategies has not progressed at a similar rate.^[^ ^15^ ^]^ This is likely due to the limited mechanistic understanding of nanomaterials’ effect on biomaterial properties. Safety is a crucial aspect of regulatory approval, and the increased bioretention times and high reactivity of nanomaterials lead to uncertainty regarding their long‐term biological effects.^[^ ^16^ ^]^ Advanced techniques including proximity labeling,^[^ ^17^ ^]^ single‐molecule localization microscopy,^[^ ^18^ ^]^ and spatial transcriptomics^[^ ^19^ ^]^ have been explored to further investigate these material‐system interactions at a molecular level. Outside of clinical translation, the further use of nanoengineered biomaterials in different applications is also limited by this lack of understanding. For example, further analysis of the mechanism of nanoparticle internalization can explain the variability in nanoparticle uptake based on sex and could also lead to the expansion of subcellular organelle targeting with nanomaterials.

This review highlights recent advancements in nanoengineered biomaterials while addressing some often‐overlooked considerations. Foremost, we discuss the classification of nanomaterials based on source, shape, or their structurally dependent properties. We then explore the fabrication of different forms of nanoengineered biomaterials. Next, we discuss parameters unique to nanoengineered biomaterials and their roles in bioactivity. Recent examples of innovative applications using nanoengineered biomaterials are discussed, focusing on the improvement of various systems due to nanoengineering. Finally, we discuss the current state of nanoengineered biomaterials research and provide an outlook on the future directions within the field.

## 2 Origins and Properties of Building Nanoblocks

**Figure 1:**
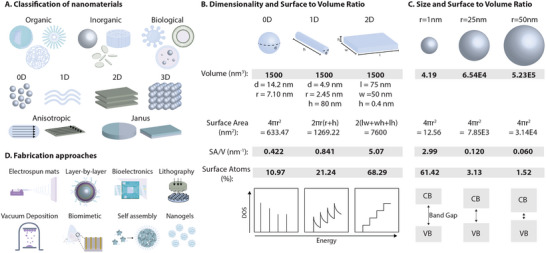
Nanoengineered biomaterials exhibit unique properties due to their source, shape, and structural characteristics. A) Nanomaterials can be classified based on their source, whether organic, inorganic, or of biological origin. The geometric dimensions of nanomaterials are also categorizing metrics, as they directly influence physical, chemical, and biological properties. Some nanomaterials have properties that vary within individual structures due to anisotropy or Janus characteristics. B) Two‐dimensional nanomaterials have a larger surface area‐to‐volume ratio than both 0D and 1D nanomaterials of the same volume. However, nanomaterial energy states become less discrete as their dimensionality increases. C) Increasing the size of nanomaterials results in a decrease in surface area to volume ratio and the percentage of atoms at the surface. Band gap size is also directly proportional to surface area to volume ratio, as larger energies are required to excite electrons. D) Nanoengineered biomaterials can be fabricated in a variety of forms, including electrospun mats, nanogels, bioelectronics, self‐assembling materials, layer‐by‐layer structures, vacuum deposited materials, lithographic, and biomimetic materials. Each of these forms exhibits unique advantages in biomedical applications. The figure was created in Adobe Illustrator using some of the icons from BioRender.com.

The ideal biomaterial is designed to achieve maximum compatibility with the host tissues. Key factors in this interaction include mechanical performance and biochemical activity. In nanoengineered biomaterials, these properties are initiated at the molecular level and largely determined by the material's composition and structure. Nanoengineered biomaterials are commonly classified by 1) their source—organic, inorganic, or biological; 2) their dimensionality—from 0D to 3D; or 3) their structural symmetry as isotropic, anisotropic, or Janus materials (**Figure** **1**A).

### 2.1 Classification of Nanomaterials By Source

Organic nanomaterials, including chitosan,^[^ ^20^ ^]^ polydopamine,^[^ ^21^ ^]^ and silk fibroin,^[^ ^22^ ^]^ are naturally derived polymers valued for their cell‐instructive properties and biocompatibility. These materials are widely applied in wound healing and tissue engineering due to their inherent bioactivity. Synthetic organic nanomaterials such as poly(lactic‐co‐glycolic acid) (PLGA) offer tunable characteristics—such as degradation rate^[^ ^23^ ^]^ and mechanical stiffness^[^ ^24^ ^]^—making them well‐suited for controlled drug delivery applications.^[^ ^25^, ^26^ ^]^ Inorganic materials, such as nanoclays and silica nanoparticles, enable efficient drug loading,^[^ ^27^, ^28^ ^]^ improved cellular uptake,^[^ ^29^, ^30^ ^]^ and reduced clearance.^[^ ^31^, ^32^ ^]^ Other inorganic nanoparticles such as gold nanoparticles and graphene, offer adjustable conductivity, surface plasmon resonance, and magnetic responsiveness.^[^ ^33^ ^]^ Although some, like bioactive glass, do not exhibit inherent bioactivity, they can direct cellular responses through the release of therapeutic ions upon degradation.^[^ ^34^, ^35^, ^36^ ^]^ Biologically derived nanomaterials, composed of proteins, lipids, or nucleic acids, leverage innate biological functionality to enable precise interactions with cellular pathways. These materials are often engineered as vesicles for intracellular delivery of therapeutics.^[^ ^37^ ^]^ Despite challenges in clinical translation due to batch‐to‐batch variability, biological nanomaterials hold promise in applications such as vaccine delivery^[^ ^38^ ^]^ and tissue regeneration.^[^ ^39^ ^]^ Composite nanomaterials integrate organic, inorganic, and/or biological components to combine and enhance the functionality of individual constituents. Examples include metal–organic frameworks (MOFs), comprising metal ions and organic linkers, and layered double hydroxides, which merge inorganic clays with polymers. These hybrid systems exhibit synergistic behavior, enabling applications such as antitumor therapy^[^ ^40^ ^]^ and hemostatic intervention,^[^ ^41^ ^]^ where single‐component systems may be insufficient.

### 2.2 Nanomaterial Properties Are Shape‐Dependent

Nanomaterials can be classified by dimensionality, which strongly influences their electronic behavior, surface energy, and resulting functionalities.^[^ ^42^ ^]^ Depending on the number of spatial dimensions below 100 nm, nanomaterials are generally categorized as zero‐dimensional (0D), one‐dimensional (1D), or two‐dimensional (2D) (Figure 1B).^[^ ^43^ ^]^ 0D nanomaterials, such as metal nanoparticles, quantum dots, and fullerenes, exhibit quantum confinement in all dimensions, restricting electron motion and producing discrete energy levels.^[^ ^44^, ^45^, ^46^ ^]^ These properties enable size‐dependent fluorescence^[^ ^47^ ^]^ and tunable absorption spectra, which are widely exploited in bioelectronics,^[^ ^48^ ^]^ biosensing,^[^ ^49^ ^]^ and imaging applications.^[^ ^50^, ^51^ ^]^ 1D nanomaterials, including nanowires, nanoribbons, and nanofibers, confine electrons in two dimensions and allow axial electron and force propagation.^[^ ^52^ ^]^ In piezoelectric systems, this geometry enhances dipole alignment and electromechanical coupling. Aligned nanofibers within hydrogels^[^ ^53^ ^]^ or elastomers^[^ ^54^ ^]^ improve mechanical stiffness through anisotropic force distribution,^[^ ^55^ ^]^ while structural mimicry of native fibrous tissues enhances implant integration and regenerative outcomes.^[^ ^56^, ^57^, ^58^, ^59^ ^]^ 2D nanomaterials, such as nanosheets and nanodiscs, confine electrons in one dimension and possess a high surface area‐to‐volume ratio (SA/V), resulting in elevated surface energy and chemical reactivity (Figure 1C).^[^ ^60^, ^61^, ^62^, ^63^ ^]^ These materials facilitate high‐efficiency drug loading and sustained release,^[^ ^64^, ^65^ ^]^ and their intercalation into polymer matrices enhances mechanical properties.^[^ ^66^ ^]^ 2D materials are widely used in photothermal therapy,^[^ ^67^ ^]^ small molecule and protein delivery,^[^ ^68^, ^69^ ^]^ and thermoelectrics.^[^ ^70^, ^71^ ^]^ 3D nanomaterials are formed by assembling lower‐dimensional units (0D, 1D, or 2D) into hierarchical architectures. Their properties depend on the structure and synergy of their nanoscale components. For example, assemblies of 0D nanoparticles show enhanced Raman signals^[^ ^72^ ^]^ and porosity;^[^ ^73^, ^74^ ^]^ annealed 1D fibers offer high elasticity and thermoelectric efficiency;^[^ ^75^, ^76^, ^77^ ^]^ and 2D‐layered composites enhance drug absorption and cell adhesion.^[^ ^78^, ^79^ ^]^ These complex architectures integrate nanoscale function with macroscale form, offering multifunctional performance not achievable with individual nanostructures.

### 2.3 Structural Variability of Nanomaterial Properties

In addition to electron confinement, the size of nanomaterials also affects their directional variability, or structural anisotropy (Figure 1A). In 0D nanomaterials, properties are usually isotropic, or independent of direction. This limits the extent to which changes in size affect material properties. As aspect ratio is increased, anisotropy is introduced, enhancing tunability and stimuli responsiveness.^[^ ^80^ ^]^ 1D and 2D nanomaterials are inherently anisotropic as these materials exhibit unique properties along their non‐nanoscale axes. Nanowires and nanofibers exhibit high magnetism^[^ ^81^ ^]^ and strain‐stiffening properties^[^ ^82^ ^]^ in the axial direction, while nanosheets have strong optical absorption along crystallographic directions. A special case of structural asymmetry is seen in Janus nanomaterials, named after the two‐faced Roman God (Figure 1A). These materials contain two distinctly contrasting properties in separate regions of their structures.^[^ ^83^ ^]^ Unlike the directional dependence of anisotropic materials, Janus characteristics contrast without respect to orientation. This duality enables the integration of diverse components and is often utilized in designing nanomotors,^[^ ^84^ ^]^ facilitating dual drug delivery,^[^ ^85^ ^]^ and eliciting strong responses to multiple external stimuli.^[^ ^86^ ^]^ While anisotropy and Janus properties are separate, nanomaterials can be engineered to exhibit both characteristics. For example, selective crosslinking of two‐phase emulsions was performed to produce anisotropic hemispherical gels with separate hydrophobic and hydrophilic regions.^[^ ^87^ ^]^ The asymmetrical wettability allowed for selective functionalization of different regions of the particles, further amplifying their Janus characteristics.

## 3 Fabrication Approaches for Nanoengineered Biomaterials

### 3.1 Introducing Biomimicry for Improved Biological Activity

Emulating natural biological processes is an important criterion in biomaterial design. This can be achieved by using naturally derived polymers that provide native biochemical and structural cues, delivering growth factors or biomolecules that guide cellular behavior in a physiological manner, or incorporating stem‐cell‐based strategies that replicate tissue regeneration and repair mechanisms. However, each of these approaches only considers a portion of the complex physiological systems present. Biomimetic systems provide a more comprehensive approach to tissue engineering, addressing the interplay of biophysical and biochemical cues in natural biological environments (Figure 1D). By designing a system that can recapitulate the mechanical properties and chemical composition of its target environment, regenerative activity is enhanced. For example, nanocarriers that mimic the morphology and function of human cells enhanced loaded enzyme activity and avoided rapid clearance.^[^ ^88^ ^]^ Through electrical induction, a nanofiber‐polymer composite was stimulated to orient itself in a cylindrical shape resembling the outer layers of natural blood vessels. These biomimetic grafts improved endothelial and smooth muscle cell activity, leading to rapid regeneration of functional blood vessels.^[^ ^89^ ^]^

### 3.2 Dynamic Spatiotemporal Activity of Self‐Assembling Structures

The supramolecular assembly of amphiphilic molecules into well‐organized structures offers a strategic approach to modulate their spatial and temporal characteristics, thereby enabling more precise control over the assembly process (Figure 1D).^[^ ^90^ ^]^ The kinetic and thermodynamic factors that enable local and specific molecular interactions, such as van der Waals forces, π–π stacking, van der Waals forces, hydrophobic interactions, and hydrogen bonding help maintain molecules in a stable state, reaching a condition of minimal energy in the system.^[^ ^91^ ^]^ Biocompatible precursors such as peptides, polymers, or metallic nanoparticles non‐destructively enter the cell and are triggered to form reactive monomers by changes in pH, enzymatic activity, reactive oxygen species (ROS), or glutathione levels.^[^ ^92^ ^]^ These supramolecular systems are especially advantageous for targeted delivery and cancer treatment, as intracellular aggregation can prolong the bioavailability of therapeutics^[^ ^93^ ^]^  and lead to improved antitumor immunity.^[^ ^94^ ^]^ For example, manganese dioxide nanoparticles were modified with either adamantane or beta‐cyclodextrin, leading to intracellular aggregation. Upon assembly, the bioresponsive particles reduced premature efflux and performed ROS depletion, O_2_ generation, and curcumin release to stimulate macrophage activity.^[^ ^94^ ^]^ Intracellular self‐assembly is also advantageous for biosensing and imaging applications, as cytoplasmic glutathione reduction can activate the formation of nanospikes on internalized microgels to enhance their fluorescent intensity for microscopic imaging.^[^ ^95^ ^]^ Moreover, the mechanical stress generated during self‐assembly process results in disrupting cellular structures, leading to cell death.^[^ ^96^ ^]^ These assemblies also exhibit photo/chemical stability and extended half‐life compared to small molecular contrast agents, making them highly effective in photoacoustic imaging.^[^ ^97^ ^]^ Synthetic nanomaterials can also be engineered to mimic the process by which dynamic proteins assemble to form complex intracellular networks.

### 3.3 Utilizing Electrostatic Interactions for Layer‐By‐Layer Materials

Inspired by the reversible condensation mechanism of DNA‐histone complexes, layer‐by‐layer nanomaterials represent an innovative strategy with potential in antibacterial therapy and modulating cell behavior (Figure 1D).^[^ ^98^ ^]^ Electrostatic interactions predominantly govern these processes, although van der Waals forces, hydrophobic interactions, and hydrogen bonding may also be harnessed to influence assembly. By leveraging the metal oxides, or clay barriers within the layers, the properties of these systems can be tailored further.^[^ ^99^ ^]^ In a recent study, by combining self‐healing technology and layer‐by‐layer assembly, a single injection vaccine was successfully reported.^[^ ^100^ ^]^ They have also been applied to the field of cancer therapeutics to achieve combined therapeutic and diagnostic utilities.^[^ ^101^ ^]^ These systems have also found utility toward developing metallic nanoparticles‐based hybrid nano‐entities for enhanced antimicrobial activity.^[^ ^102^ ^]^

### 3.4 Nanoengineered Hydrogels Emulate Extracellular Matrices

Due to their ability to replicate the complexity of the cell and tissue microenvironment, hydrogels have gained significant interest in the field of regenerative medicine (Figure 1D).^[^ ^103^ ^]^ Techniques such as lithography, 3D printing, and electrospinning which can help emulate the spatial characteristics of the extracellular matrix and tissue have enabled the field to better recapitulate the microenvironment by incorporating these hydrogels as scaffolds.^[^ ^104^ ^]^ On the nanoscale level, these nanogels have adjustable sizes ranging from a few nanometers to several thousand nanometers and a large surface area for multivalent bioconjugation. Additionally, they possess an internal network for incorporating biologically related molecules. By structurally engineering these nanogels environmental responsiveness, therapeutic delivery, and molecular recognition could be achieved simultaneously.^[^ ^105^ ^]^ Their applications are well reported in the field of bioimaging and biosensors as well.^[^ ^106^ ^]^

### 3.5 Electrospun Mats Form Nanofibrous Scaffolds

The large surface area relative to volume, processing flexibility, and controllable phase structure associated with electrospun mats opens them up to numerous biomedical applications (Figure 1D).^[^ ^107^ ^]^ Technological advancements have allowed the fabrication of electrospun fibers with aligned, patterned arrangements to direct cell alignment.^[^ ^108^ ^]^ The need to find fouling‐resistant and hydrophobic materials has resulted in researchers trying to identify newer polymeric candidates. One such polymer that has demonstrated simultaneous hydrophobicity and resistance to protein adsorption is zwitterionic amphiphilic copolymer poly(trifluoroethyl methacrylate‐random‐sulfobetaine methacrylate).^[^ ^109^ ^]^ These electrospun mats can also be seamlessly integrated with other technologies. In a recent study, electrospun multi‐walled carbon nanotubes containing polycaprolactone/silk fibroin nanofibers were used to stack brown adipose‐derived stem cells. These nanofibers demonstrated accelerated angiogenesis and decreased inflammation compared to no treatment.^[^ ^110^ ^]^

### 3.6 High‐Resolution Lithography for Smart Biomaterials

Lithography‐based techniques offer precise spatial control over surface topography at micro‐ to nanoscale resolutions, making them highly valuable in bioengineering applications (Figure 1D).^[^ ^111^ ^]^ Early work in photolithography utilized UV light to generate microscale patterns by selectively exposing specific regions of material surfaces, enabling the fabrication of patterned scaffolds and alignment of cellular growth.^[^ ^112^ ^]^ Advances in electron beam lithography have pushed resolution limits further due to the shorter wavelengths of electron beams. Electron beam lithography has enabled the direct‐write patterning of nanoscale features such as proteins, with significantly greater precision than traditional photolithography.^[^ ^113^ ^]^ For example, polymers bearing pendant trehalose groups have been developed as negative resistors that both crosslink to surfaces and protect sensitive biomolecules from damage during vacuum exposure and electron irradiation.^[^ ^114^ ^]^ To meet the needs of large‐area patterning and scalable production, particularly in semiconductor and micro‐electro‐mechanical systems, ultraviolet‐assisted nanoimprint lithography has been adopted for its high‐throughput capabilities and resolution.^[^ ^115^ ^]^ Recently, ultrasonic nanoimprinting has emerged as a room‐temperature technique that uses focused ultrasonic energy to create nanostructures with high fidelity. This process, driven by alternating dislocation generation and recovery, enables rapid fabrication with fine dimensional control across a wide range of substrates, supporting scalable production of nanostructured biomaterials for applications such as biosensing.^[^ ^116^ ^]^ Multiphoton lithography has further extended lithographic capabilities into the third dimension, enabling the fabrication of complex 3D microstructures using organic semiconductor composites.^[^ ^117^ ^]^ These constructs demonstrate high electrical conductivity and preserve biomolecular activity, such as laminin incorporation, suggesting utility in bioelectronics and biosensor fabrication. Together, these lithographic techniques facilitate the fabrication of smart biomaterials with programmable architectures, high spatial precision, and application‐specific functionality for next‐generation biomedical devices.

### 3.7 Designing Functionality with Vacuum‐Deposited Surfaces

Vacuum deposition enables precise thin‐film fabrication at the atomic or molecular scale, critical for advanced electronics, nanotechnology, and biomaterials (Figure 1D). The different methods for vacuum deposition can either be classified as physical, where the material is physically vaporized and condensed onto the surface, or chemical, which relies on chemical reactions to form a solid film on the substrate.^[^ ^118^ ^]^ Recently, the sequential process of atomic layer deposition has emerged as an alternative, wherein precursors and reactants are introduced separately, ensuring self‐limiting deposition and precise control over nanofilm thickness. This self‐limiting property enhances uniformity and step coverage, especially on large areas or high‐aspect‐ratio structures. Atomic layer deposition also finds use in biomedical applications including chemotherapy. The precise fabrication method allows for controlled loading of cobalt oxide into titanium oxide pores, optimizing energy band structures for efficient sonodynamic therapy. Forming a heterojunction between porous titanium dioxide and cobalt oxide enhanced the production of reactive oxygen species and boosted antitumor effects.^[^ ^119^ ^]^

## 4 Material Properties at the Nanoscale

**Figure 2:**
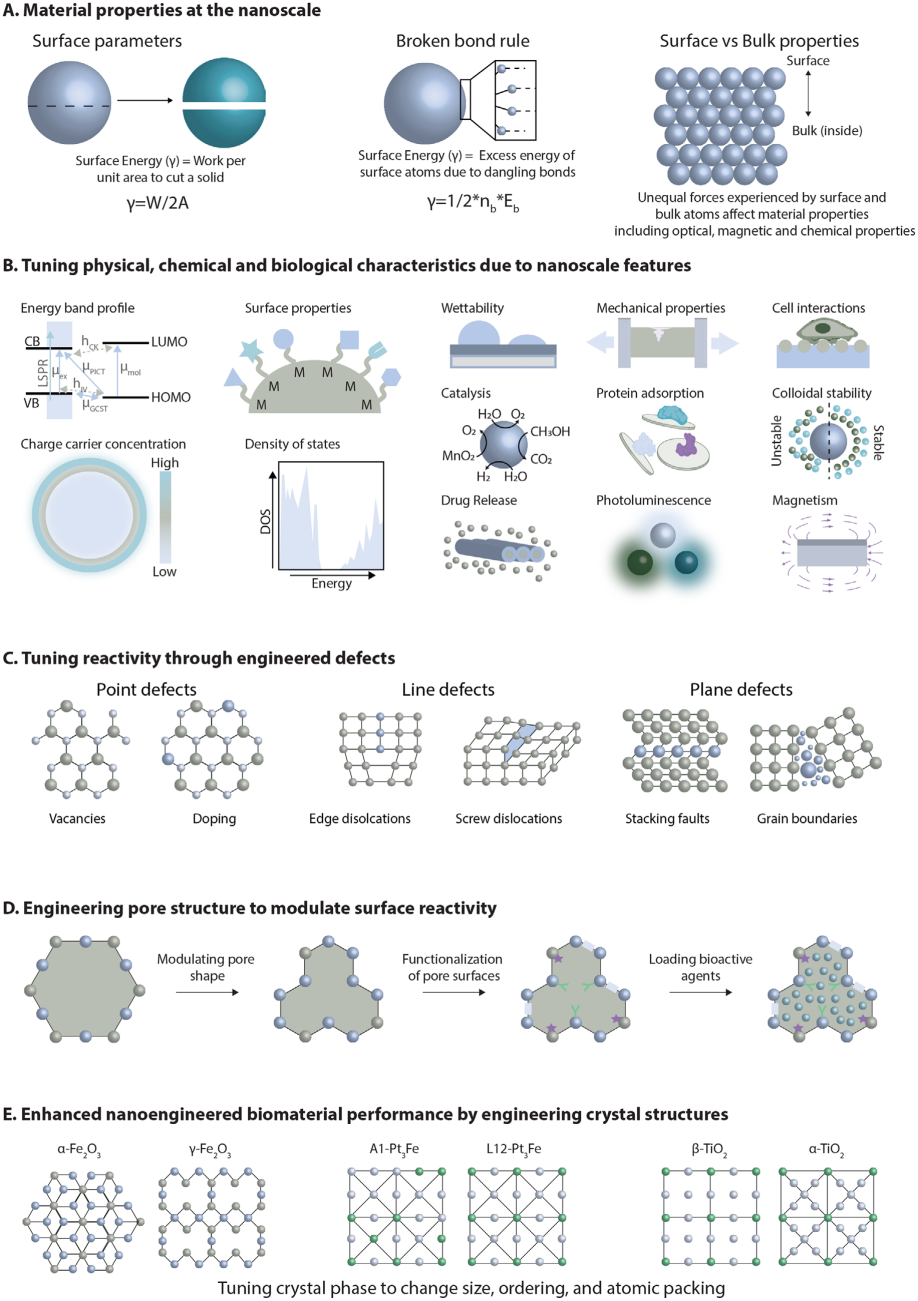
Engineering nanomaterial surface parameters through modulating crystal phases, defects, or pore surfaces. A) The surface energy of nanomaterials depends primarily on the presence of dangling bonds at the surface. Unequal intermolecular forces between surface and bulk atoms in nanomaterials can result in surface atoms displaying unique characteristics. B) Surface energy directly influences a wide range of material properties, including adhesion, stability, protein adsorption, and magnetism, among others. Engineering surface properties enable the optimization of nanoparticles for various biomedical applications. C) By engineering 0D point defects, 1D line defects, or 2D plane defects, the reactivity of nanoengineered materials can be tuned. D) Modifying void shape, surface chemistry, or loading cargo within porous nanomaterials can affect the reactive surface area. E) By modulating the inherent crystal structure of nanomaterials, their performance in a variety of biomedical applications can be enhanced. The figure was created in Adobe Illustrator using some of the icons from BioRender.com.

Classical thermodynamics and continuum mechanics effectively explain the properties of macroscale systems but become less applicable at the nanoscale. This limitation arises from factors such as increased surface or interface trapping, quantum confinement, the discrete nature of atoms, and structural relaxation. Coordination deficiency and atomic bond contraction at interfaces or boundary layers of nanosystems result in distinct properties compared to their bulk counterparts, as evident in characteristics such as melting points, electrical conductivity, and Young's modulus.^[^ ^120^ ^]^ These differences underscore the need to focus on surface characteristics to better understand the complex interplay of nanoscale phenomena (**Figure** **2**A). At the nanoscale, material properties are predominantly governed by surface characteristics due to the high surface‐to‐volume ratio. As described by the broken bond rule, unsatiated dangling bonds at nanomaterial surfaces lead to heightened reactivity and altered behaviors, such as reduced melting points. Key surface parameters, including surface energy, wettability, and charge, critically influence interfacial interactions and functionalities, driving their importance in a wide range of nanoengineered biomaterial applications.

The catalytic activity of gold nanoparticles can be enhanced by specific crystal facets, while MoS_2_ films can be tuned for surface energy and wettability through variations in crystallinity and thickness.^[^ ^121^ ^]^ In silicon nanowires, surface stress can affect mechanical behavior and electromechanical performance.^[^ ^122^ ^]^ These studies highlight how surface parameters play a pivotal role in governing the properties of nanomaterials and how the study of underlying thermodynamic principles is crucial for designing nanomaterials to meet the specific application.

### 4.1 Hill's Nanothermodynamics: Role of Subdivision Potential

One of the earliest efforts to this end came through Terrell L. Hill who in 1962 generalized the Gibbs equation of classical thermodynamics to extend it also for small systems.^[^ ^123^ ^]^ Through this approach, he pioneered a new framework that would later be termed “nanothermodynamics”—a specialized extension of classical thermodynamics focused on understanding the unique energetic and statistical behaviors of systems with nanoscale dimensions. His work focused on introducing the finite size effects within the macroscopic thermodynamics framework and incorporated the concept of subdivision potential (ℰ). Hill's formulation of nanothermodynamics is grounded in the ensemble approach for small systems. As per Hill's work, nanosystems require the introduction of a composite intensive property at the ensemble level (subdivision potential). The internal energy is now given as

(1) $$\partial \;U_{t}=T\partial S_{T}-p\partial V_{t}+\sum_{i}\mu_{i}\partial N_{it}+\varepsilon \partial N$$

where chemical potential called the sub‐division potential is introduced and given by:

(2) $$E=\left(\partial U_{t}/\partial N\right)\;St,V_{t},N_{it}$$

This becomes important at the nanoscale since S_t_, V_t_, and N_it_ are all held constant macroscopic systems. In nanosystems, if an assembly of small units is subdivided, 𝒩 will increase while V and N_i_ decrease, highlighting size effects.

In the design of self‐assembling biomaterial complexes, Hill's small‐system thermodynamics can provide valuable insights into their stability and solution behavior. One illustrative study proposed a comprehensive thermodynamic framework to describe the interactions between polymers and surfactants by treating these complexes as semi‐open small systems composed of fluctuating subsystems.^[^ ^124^ ^]^ The authors described these at a macroscopic, small‐system, and subsystem levels of organization. The model used the excess free energy term (ℰ), which captures energy changes arising from fluctuations and finite‐size effects. Interestingly, it was determined that polymer's molecular weight had no influence on polymer–surfactant interactions and that the average number of surfactants per aggregate rises as more surfactant binds. These findings underscore the potential of small‐system thermodynamics to inform the design and understanding of engineered nanostructures.

### 4.2 Internal Energy Dependence on Surface Properties

Phenomena such as melting point depression in nanomaterials underscore the need to highlight the effect of a high surface area to volume ratio associated with nanoparticles alongside accounting for the finite size effects as discussed above. Traditionally, surface thermodynamics has focused on surfaces on bulk systems under constant pressure assumptions. However, more recent work, especially with nanoparticles, has incorporated the varying pressure term, considering the assumption that internal pressure can be significantly higher than external pressure.^[^ ^125^ ^]^ To derive this relationship, consider an ideal one‐component system that consists of an isotropic homogeneous condensed phase (_C_) and an isotropic homogeneous fluid phase (_F_). These phases are separated by the surface of tension (_S_), an isotropic surface with negligible thickness. Based on previous derivations, the internal energy function (U) for the formation of a new surface at constant temperature (T), pressure (P), shape, and strain, considering proportional variations in the entropy (S), volume (V), area (A), and number of atoms (N), can be expressed as:^[^ ^125^ ^]^

(3) $$\begin{array}{ccc} U & = & \left(T_{C}S_{C}-P_{C}V_{C}+\mu_{C}N_{C}\right)+\left(T_{F}S_{F}-P_{F}V_{F}+\mu_{F}N_{F}\right)\\ & & +\left(T_{S}S_{S}+\gamma A\right) \end{array}$$

(4) $$\;U_{S}=U-\left(U_{C}+U_{F}\right)$$

(5) $$\;U_{S}=T_{S}\;S_{S}+\gamma A$$

where *γ is* the intensive surface parameter associated with the surface area change dA. Thus, the surface entropy (S_s_) and energy (γA) are considered as excess entropy and energy on account of the presence of the surface of tension over the entropy or energy that would exist in the same system if the surface of tension were absent, while all other relevant parameters remain unchanged.

The Gibbs‐Duhem relation for surfaces involves looking at the changes in specific surface free energy (γ) related to variations in the surface excesses of components and temperature.

(6) $$\;U_{S}=T_{S}\;S_{S}+A\partial \gamma +\left(\gamma -\gamma_{s}\right)\partial A=0$$

Mechanistically, the Gibbs‐Duhem relation for surfaces could be used toward explaining the decrease in melting point for nanoparticles relative to bulk materials. This has been ascribed to the stepwise melting of small particles, beginning with surface melting followed by the solid core. This size‐dependent effect can be strategically utilized to lower the processing temperatures of lead‐free metallic materials. A recent investigation demonstrated the fabrication of tin‐silver alloy nanoparticles with reduced melting points by tailoring surface energy through particle size reduction and the incorporation of surfactants or stabilizers.^[^ ^126^ ^]^ By reducing the average nanoparticle diameter from 64 nm to 10 nm, the authors succeeded in lowering the melting temperature from 220 °C to 194.3 °C and highlighted their use as lead‐free solders with low melting points. Additionally, they observed a broadening of the phase transition, which was attributed to the finite size effect. By understanding the Gibbs‐Duhem relation, the internal energy of different nanoparticles can be engineered, expanding their applications in catalysis and photothermal therapy.

### 4.3 Surface Energy Is a Product of Dangling Surface Bonds

The cleavage of any solid material results in breakage of existing atomic bonds resulting in altering the prior atomic environment for relaxed surface atoms which are now devoid of atomic neighbors present in the bulk state. Surface energy can also be calculated semi‐empirically by the broken bond rule. In this equation, surface energy is described as the energy required to form a unit area of a new surface, where *W* is the reversible work involved. The subscripts *h*, *k*, and *l* are Miller indices that specify the orientation of a plane. Therefore, the surface energy is equivalent to the total binding energy (*E_b_*) of the surface atoms (*n_hk_*_l_).

(7) $$\;\gamma_{hkl}=\frac{W_{hkl}}{2A_{hkl}}=\frac{n_{hkl\;}E_{b}}{2}$$

While dangling bonds were once thought to arise solely from surface cleavage, recent research has focused on deliberately engineering surfaces to create and control these reactive sites. A notable example is the precise manipulation of dangling bonds on semiconductor silicon surfaces using scanning tunneling microscopy to tailor new quantum‐well states.^[^ ^127^ ^]^ This study reported that the interacting dangling bonds could demonstrate new excited states in the system thereby demonstrating potential of atomic‐scale quantum engineering.

### 4.4 Surface Energy Determines Nanoengineered Biomaterial Properties

Owing to their large surface area to volume ratio, nanomaterials are characterized by significantly elevated surface‐free energies.^[^ ^128^ ^]^ Surface free energy can be thought of as the increase in energy associated with taking an atom from the bulk of a material and placing it at the surface, creating unsatiated dangling bonds.^[^ ^129^ ^]^ Another way to think about surface energy (γt) is commonly and conveniently by looking at its additive polar (γp) and dispersive (γd) components. In surface energy considerations, dipole–dipole, and  hydrogen bonding are drawn together as polar forces while dispersion forces include van der Waals dispersion forces or London forces.^[^ ^130^ ^]^ Surface energy also controls size‐dependent characteristics of nanoparticles such as crystal structure and chemical stability, as reflected in turn in physical and chemical properties like melting temperature, interaction binding energy, Debye temperature, heat capacity, and the solubility. It can also influence biological properties like cell uptake and protein adsorption. By analyzing surface energy, one can better understand the factors influencing particle stability, including morphology, dimensions, crystal structure, and temperature‐dependent behavior.^[^ ^131^ ^]^

The Gibbs free energy of a nanoparticle system is composed of the bulk and surface free energies. Due to the substantial number of atoms present on the surface of nanomaterials, the surface contribution becomes more significant.^[^ ^132^ ^]^ This is crucial for understanding the stability of phase‐transforming nanoparticles. It is well established that differences in the surface energy between two polymorphs of a material can lead to a reversal in their Gibbs free energies at the nanoscale, altering their relative phase stabilities.^[^ ^132^ ^]^ This phenomenon of phase stability reversal has been experimentally observed in nanocrystalline titania.^[^ ^133^ ^]^ Thus, the factors influencing thermodynamic equilibrium, which determines the structures and properties of nanomaterials, should be rigorously investigated. The high surface free energy associated with inorganic nanoparticles can also result in them aggregating into larger particles thereby influencing their stability. Thereby careful consideration of attractive and repulsive forces influencing a nanoparticle is critical.

Early seminal work by Pawlow successfully demonstrated the effects of surface energy and size on the melting temperature of a particle.^[^ ^134^ ^]^ He also investigated the difference in the melting point of nanoparticles from that of bulk material. From his observations, the temperature shift ΔT between the surface and bulk of a spherical particle can be obtained by the relation:

(8) $$\Delta T=\frac{\frac{2\gamma }{R\Omega s}}{\eta_{l}-\eta_{s}}\;$$

where R describes the particle radius, *γ* is the surface energy per unit area, *Ω*_s_ is the molar volume, and *η* describes the specific entropy per mole. Interestingly, a size effect reflected by (2/*R*) comes into play, since the surface energy *γ* is energy per unit area. This equation indicates the significant difference in melting temperatures between smaller particles and bulk materials.

Shape‐controlled inorganic nanomaterials can be generated via tuning their nucleation and growth through experimental conditions. From the early work of Mullin, nucleation has been defined as a process whereby a second phase is generated from one phase.^[^ ^135^ ^]^ The homogeneous formation of nuclei is considered a thermodynamic process, driven by supersaturation and determined by the total free energy (*ΔG*) of a nanoparticle. This total free energy is the sum of the surface and bulk‐free energies (*ΔG_v_*), as expressed in the equation:

(9) $$\Delta G=4\pi r^{2}\gamma -\frac{4}{3}\left(\pi r^{3}\Delta G_{v}\right)$$

where *r* describes particle radius and *γ* denotes the surface energy. The free energy of the bulk crystal (Δ*G*_ν_) is the free energy change associated with the transformation to a unit volume of particles and is denoted as:

(10) $$\Delta \;G_{v}=-\;\frac{2\gamma }{r}=-\frac{2k_{B}TlnS}{v}$$

Based on prior studies, the reliance of the final surface free energy on both the surface energy and surface area has been shown. This affords an opportunity to minimize the surface free energies for a given volume to tune the synthesis of nanoparticles with anisotropic shapes.^[^ ^135^ ^]^ The Wulff construction model further points towards the influence of surface free energy and surface kinks on the growth rate of nanoparticles, with higher surface free energy or more kinks being associated with fast crystal growth rate resulting in eventually reducing surface area until equilibrium is achieved.^[^ ^136^ ^]^ However, the final morphology of the nanoparticles is determined by the slowest growth rate of the crystal surface. Spherical nanoparticles are obtained when sufficient energy is supplied to the bulk reaction solution which results in the nuclei too grow quickly under thermodynamic control.^[^ ^135^ ^]^ To address this issue, adding surfactants or additives to selectively bind or grow onto specific facets of nanoparticles is an effective strategy to alter surface properties and reduce surface tension for desirable shapes.

### 4.5 Tuning Physical, Chemical, and Biological Characteristics Due to Nanoscale Features

Nanoscale materials exhibit unique features that enable precise tuning of their physical, chemical, and biological properties, making them versatile for diverse applications (Figure 2B). Key properties such as colloidal stability, wettability, and protein adsorption are strongly influenced by surface characteristics, including surface energy, charge distribution, and morphology. Surface wettability, in particular, plays a critical role in determining nano‐bio interactions, especially within biological systems.^[^ ^137^ ^]^ Wettability is governed by surface energy, where higher surface energies typically enhance wettability by reducing the contact angle between solid biomaterials and physiological fluids.

At the nanoscale, the ability to manipulate charge carrier concentration and energy band profiles facilitates the design of advanced semiconductors and optoelectronic devices. Furthermore, tailoring the density of states and magnetism enables applications in quantum computing and magnetic storage. Nanoscale materials also exhibit distinctive photoluminescence, which supports imaging and sensing technologies. Mechanical properties such as stiffness and toughness, along with catalytic activity, can be finely tuned to improve performance in drug delivery, cell interactions, and energy systems. Together, these attributes highlight the transformative potential of nanoscale materials in addressing complex multidisciplinary challenges.

Cell attachment to the surface of biomaterials plays a key role in guiding later stages such as cell growth, specialization, and tissue development at the material‐tissue interface. Gaining insight into how surface‐free energy influences interactions between biomaterials and biological systems is essential for the informed design of functional biomaterials. Generally, surfaces with higher surface free energy or greater wettability tend to enhance cell adhesion, even when protein adsorption is limited, whereas surfaces with lower surface free energy typically do not support effective cell attachment or spreading.^[^ ^138^ ^]^ For example, reduced osteoblast adhesion occurs on materials with low wettability.^[^ ^139^ ^]^ This reduction is likely because polar molecules on wettable surfaces form chemical bonds with cell surface groups, while non‐polar molecules bond through weaker van der Waals interactions. Due to the complex nature of cell‐surface interactions and the interconnected factors involved, this relationship is not straightforward, and some studies have also found preferential cell growth on low‐energy surfaces.^[^ ^140^ ^]^

Surface energy can influence the complex dynamic interactions between nanoparticles in a biological fluid and the biomolecules (such as proteins, lipids, metabolites, and nucleic acids) that surround them.^[^ ^141^ ^]^ The complexity of the protein corona is largely due to the intricate nature of biological systems and the need for nanoparticles to reduce their surface energy. This corona can significantly influence key properties such as surface energy, surface charge, hydrodynamic radius, and aggregation/stability. Additionally, nanoparticles can cause structural and conformational changes in the proteins within the corona.

Surface energy significantly impacts a variety of material characteristics, prompting extensive efforts to optimize it for different biomaterials. In nanoengineered biomaterials, precise management of surface energy is typically achieved through strategies like coatings, functionalization with specific molecules, or altering surface topography at the nanoscale.^[^ ^142^ ^]^ This tailored approach allows researchers to enhance the performance and suitability of biomaterials for diverse biomedical uses, including implants, drug delivery systems, and tissue engineering scaffolds.^[^ ^143^ ^]^

## 5 Overlooked Considerations of Nanomaterials

### 5.1 Tuning Reactivity Through Engineered Defects

Defect engineering impacts the catalytic activity of nanomaterials by altering electronic energy states. Point defects caused by removing or replacing atoms such as oxygen, selenium, or sulfur in the nanomaterial lattice structure, form discrete energy states at certain regions within the material (Figure 2C). These localized electronic states can act as charge traps that prevent electrons in the conduction band from recombining with holes in the valence band, improving the efficiency of nanosonosensitizers^[^ ^144^ ^]^ and photocatalytic nanomaterials.^[^ ^145^ ^]^ For example, vacancies in 0D and 2D transition metal‐based nanomaterials lead to enhanced responses to ultrasound,^[^ ^144^ ^]^ near‐infrared light,^[^ ^146^ ^]^ or electrical energy.^[^ ^147^ ^]^ Additionally, the introduction of vacancies can lead to a narrowed band gap, increasing the electrical conductivity of the material. Oxygen vacancies on 2D InVO_4_ nanobelts promote interfacial charge transfer, increasing n‐type conductivity and contributing to improved CO_2_ reduction.^[^ ^148^ ^]^ In photoluminescent nanomaterials, excited electrons can recombine with defect states at a higher energy level than the valence band resulting in altered emission spectra.^[^ ^145^, ^149^ ^]^ Controlling the degree of phenyl acetylene‐doped sp^3^‐carbon defects on single‐walled carbon nanotubes led to a tunable increase in photoluminescent emission intensity.^[^ ^149^ ^]^ Defects can also generate sites with increased chemical reactivity for catalysis.^[^ ^150^ ^]^ Oxygen vacancies in Co_3_O_4_ nanocubes affected the distribution of charge density and boosted the initiation of radicals to catalyze limonene oxidation.^[^ ^151^ ^]^ While defects have also been shown to affect the magnetic properties of nanomaterials,^[^ ^152^ ^]^ a concise correlation requires further investigation. Defects change the surface reactivity of nanomaterials which can affect interactions with cells. For example, replacing oxygen atoms in Fe_3_O_4_ nanoparticles with silver modified the interactions between nanoparticles and polycaprolactone scaffolds, leading to increased surface roughness and hydrophilicity which improved cell adhesion and viability of human melanocytes.^[^ ^10^ ^]^ Similarly, improved cell signaling was achieved on TiO_2_ nanotubes by replacing oxygen with zinc and strontium. This led to significantly enhanced adhesion, proliferation, and growth of adipose‐derived stem cells compared to an unmodified surface.^[^ ^153^ ^]^ By engineering atomic vacancies into molybdenum disulfide (MoS_2_) nanostructures, their interaction with mitochondrial regulatory mechanisms can be amplified. These defect‐rich MoS_2_ nanoflowers demonstrated the ability to stimulate mitochondrial biogenesis, suggesting that tuning surface and defect properties can be a powerful strategy for directing cellular function.^[^ ^8^ ^]^

Extended defects such as line defects and grain boundaries create localized energy states that occur on larger regions of the crystal structure. In addition to electronic and chemical behaviors, these localized states can impact the mechanical properties of nanomaterials. Dislocations are line defects that create mobile lattice distortions around which the defect states alter stress distribution throughout the material.^[^ ^154^ ^]^ Within nickel‐based single‐crystal superalloys, increasing the density of dislocations caused for a higher creep resistance.^[^ ^155^ ^]^ Grain boundaries occur at the interface of differently oriented regions of the crystal lattice.

These defects prevent the movement of dislocations which increases the mechanical strength in nanomaterials. Tuning the orientation of grain boundaries in metallic nanocrystals can impart pseudo‐elasticity, allowing for improved recovery from large strains.^[^ ^156^ ^]^ Forming soft grain boundaries in brittle oxide perovskite ceramic nanofibers led to improved flexibility, indicated by structural stability after cyclic 40% strains.^[^ ^157^ ^]^ The impact of extended defects on nanomaterial mechanical properties can also result in altered biological behavior. Copper nano‐precipitates in an iron‐manganese alloy led to grain refinements which improved the biodegradation rate and cell viability of osteosarcoma fibroblasts.^[^ ^158^ ^]^

### 5.2 Engineering Pore Structure to Modulate Surface Reactivity

Tuning the shape, surface properties, and therapeutic cargo within porous nanomaterials provides control over the reactive surface area to improve performance in various biomedical applications (Figure 2D). Catalytic properties are improved by the addition of pores as there are more accessible sites for chemical reactions.^[^ ^159^ ^]^ Controlling the void volume and size in core‐shell Ni‐Ru nanoparticle catalysts enhanced the diffusion efficiency of reactants and products, leading to 99% conversion of isosorbide‐diketone to diaminoisomannide.^[^ ^160^ ^]^ High porosities are also beneficial for exposing electrochemically active sites in energy storage devices.^[^ ^161^ ^]^ In zinc ion hybrid supercapacitors based on porous carbon nanosheets, pore sizes of 7.5 A or larger provided a low energy barrier for the diffusion of Zn^2+^ ions.^[^ ^162^ ^]^ The loading efficiency and release rates of therapeutic delivery devices are increased in highly porous structures.^[^ ^163^ ^]^ In a MOF‐based nanocarrier, increasing the pore size from 2.2 to 2.6 nm led to a 32% increase in loading capacity of paclitaxel.^[^ ^164^ ^]^ Pore engineering in polylactic acid nanofibers improved the release kinetics of antibacterial nanoparticles, leading to antibacterial efficiencies of over 99% against *Escherichia coli*.^[^ ^165^ ^]^ Interconnected pores in tissue scaffolds encourage the growth and migration of encapsulated cells.^[^ ^166^ ^]^

Bilayer scaffolds based on cellulose nanofibers were engineered to have specific pore sizes to simulate the epidermis and dermis skin layers, providing fibroblasts and keratinocytes with a biomimetic porous structure.^[^ ^167^ ^]^ Additionally, gas‐sensing nanomaterials achieve enhanced diffusion and accessibility of reactive sites upon the introduction of porosity.^[^ ^168^ ^]^ Zinc oxide nanorods rich with surface pores were used to detect n‐butanol with a low detection limit near 0.1ppm and a high selectivity relative to non‐surface engineered nanorods.^[^ ^169^ ^]^ By packing linear polymers within the pores of a 2D covalent organic framework, hierarchal pore structures with enhanced gas permeability and selectivity were achieved. The control over pore structure led to a reduction in the diffusion of bulky molecules but increased preferential transport of H_2_.^[^ ^170^ ^]^

### 5.3 Enhanced Nanoengineered Biomaterial Performance by Engineering Crystal Structure

Crystallinity engineering in nanomaterials involves enhancing the inherent crystal structure to impart specific properties for different applications. Optimizing crystal size or phase can affect the catalytic activity, mechanical properties, or the stimuli responsiveness of nanomaterials (Figure 2E). Controlling crystal growth can improve the catalytic properties of nanomaterials as smaller crystals provide a higher surface area of reactive sites.^[^ ^171^ ^]^ Tuning the *c*‐axis thickness of mordenite zeolite crystals to 40 nm significantly enhanced the catalysis of dimethyl ether carbonylation.^[^ ^172^ ^]^ Due to the Hall Petch effect, smaller crystal sizes lead to increased strength.^[^ ^9^ ^]^ Molecular dynamics simulations of copper‐nickel alloys confirmed this relationship in nanocrystals larger than 11.5 nm, showing that deformation was dominated by dislocation propagation. However, in smaller crystals, grain boundary sliding dominated deformation, resulting in an inverse Hall Petch effect.^[^ ^173^ ^]^ The increased number of surface atoms present on smaller crystals also affects how the nanomaterial responds to magnetic fields. By reducing the crystal size of iron oxide nanoparticles from 37 to 10 nm, precise manipulation from ferromagnetism to superparamagnetism was achieved.^[^ ^174^ ^]^

As some crystal phases are more active than others, phase engineering regulates bonding between catalytically active sites of nanomaterials and reactive intermediates. This allows for better control over the efficiency of chemical reactions. For example, a transformation from a disordered A1 crystal phase to an ordered L1(2) phase boosted the rate of acetylene hydrogenation two‐fold in Pt_3_Fe nanoparticles.^[^ ^175^ ^]^ Etching unconventional 4H and 2H phases in heterophase gold nanostructures led to excellent catalysis of the hydrogen evolution reaction, comparable to a commercial catalyst.^[^ ^176^ ^]^ Changing the crystal phase of a nanomaterial can also affect its interactions with other materials, impacting the mechanical properties of nanocomposites.^[^ ^177^ ^]^ Nano‐sized vanadium precipitates with two different crystal phases were separately alloyed with high‐nitrogen austenitic stainless steel. The MeX‐type precipitates with a face‐centered cubic structure maintained semi‐coherence with the stainless‐steel matrix, providing enhanced strength without sacrificing ductility. Me_2_X precipitates with a hexagonal close‐packed structure formed an incoherent relationship with the matrix which led to a weaker, more brittle composite.^[^ ^178^ ^]^ Different crystal phases can have different electronic band gaps which affects luminescent properties as different energies are required to emit light.^[^ ^179^ ^]^ In a recent study, water‐induced phase transformations allowed the conversion of blue light‐emitting Cs_3_Cu_2_I_5_ nanocrystals to yellow light‐emitting Cs_3_Cu_2_I_3_ crystals.^[^ ^180^ ^]^ Additionally, it was determined that by controlling crystal phase growth through cadmium content in CsPb_1‐_ *_x_* Cd*_x_* Br_3_ nanorods, the band gaps, emission spectra, and conductivity were effectively tunable.^[^ ^181^ ^]^

## 6 Applications of Nanoengineered Biomaterials

In this section, we will review the strategies that have leveraged the attractive properties of nanomaterials to improve the performance of biomaterials in different applications.

### 6.1 Nanoengineered Biomaterials for Regenerative Medicine

Regenerative medicine involves promoting the restoration of damaged tissues through one of two main approaches: acellular strategies or cell therapies. In the first approach, regeneration is achieved with tunable, bioactive materials that provide native cells with the proper microenvironment to proliferate and form tissues. The second approach involves delivering exogenous cells that can address potential deficiencies in cellular processes, leading to healthy tissue formation. Nanomaterials are incorporated into these strategies to improve their efficiency.

#### 6.1.1 Leveraging the Inherent Bioactivity of Nanomaterials

**Figure 3:**
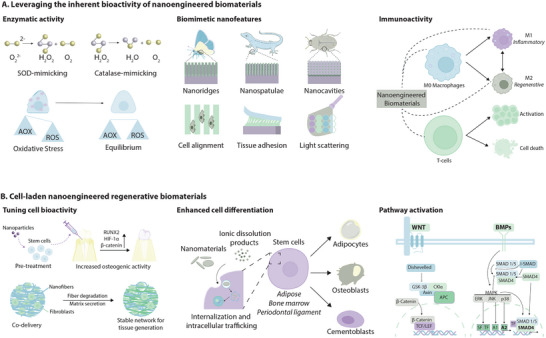
Nanoengineered biomaterials promote tissue regeneration through inherent bioactivity and enhanced cellular functions. A) Nanomaterials can improve the regenerative potential of damaged tissues by clearing intracellular reactive oxygen species, mimicking the bioactivity of enzymes. Additionally, naturally existing nanostructures are often replicated in nanoengineered biomaterial design for improved cellular interactions, bulk physical properties, and stimuli‐responsiveness. The inherent immunoactivity of nanomaterials results in the fabrication of biomaterials that achieve positive interactions with the natural immune system for efficient regeneration. B) Pre‐treating cells with nanomaterials can lead to improved regenerative signaling for applications including osteogenesis, while co‐delivery of nanomaterials with cells can provide a dynamic network for cells to adhere and proliferate throughout the regenerative process. Cellular internalization of nanomaterials or their dissolution products enhances their differentiation to specific lineages. The positive effects on regeneration are also due to the activation of different signaling pathways. The figure was created in Adobe Illustrator using the icons from BioRender.com.

To achieve the level of cellular activity required for tissue regeneration, a conducive extracellular microenvironment is crucial. This microenvironment involves a complex network of biochemical and biophysical cues that result in the activation and differentiation of stem cells to form tissues. Multi‐component biomaterial systems seek to address the complexities involved in creating a regenerative microenvironment. Generally, natural polymers function as the base material, imparting biocompatibility to the system. Different biophysical aspects such as mechanical properties, hydrophobicity, and biodegradation profiles can also be controlled with different base polymers. The encapsulation and sustained release of small molecule therapeutics, minerals, or proteins provide biochemical cues to stimulate regeneration in situ. Often, these systems result in non‐specific delivery and rapid clearance, reducing clinical potential. In these acellular systems, nanomaterials are leveraged for their inherent biochemical activity; specifically, their enzyme‐like conversion activity and biomimetic nanofeatures (**Figure** **3**A).

By incorporating copper/tannic acid‐based nanoparticles in a crosslinked silk fibroin network, an anti‐inflammatory osteochondral scaffold was formed. Tannic acid is inherently antioxidative, indicated by its ability to reverse intracellular glutathione depletion and prevent Fenton oxidation by complexing iron ions. Synergistic antioxidant effects were observed in the nanoparticles as coordination of the copper and tannic acid leads to the formation of a crystalline structure with enzyme‐like scavenging activity. Further, the particles enhanced regeneration by reversing the pro‐inflammatory effects of interleukin‐1 beta.^[^ ^182^ ^]^ This is due to the ability of tannic acid to chelate with metal ions, forming hydrophilic metal‐phenolic network surfaces with significantly elevated free energies that promote cell adhesion and proliferation.^[^ ^183^ ^]^ Manganese dioxide nanosheets also exhibit enzyme‐like characteristics, catalyzing the conversion of H_2_O_2_ to O_2_. When incorporated within a hyaluronic acid hydrogel, this allowed for the reduction of hypoxia and oxidative stress, promoting diabetic wound healing in mice.^[^ ^184^ ^]^ Exosomes derived from pro‐regenerative M2 macrophages were also added to the hydrogel system. In vitro, the exosomes stimulated blood perfusion and increased the population of CD31/CD34_+_ cells in the wound, indicating enhanced angiogenesis.

In some cases, acellular techniques utilize structural nanofeatures to provide biophysical regenerative cues. Materials with nanoscale roughness mimic components of the natural extracellular matrix, supporting cell adhesion and proliferation. Poly (lactic acid)‐based nanofibers within a nerve scaffold promoted revascularization through directing and accelerating endothelial cell migration. The alignment of fibers emulated the topology of the fibrin cables that form after a peripheral nerve injury, providing similar cues to facilitate nerve regeneration.^[^ ^185^ ^]^ A similar effect was observed by fabricating a peripheral nerve scaffold with Morpho butterfly wings. The nanoridges present on the wings promoted the elongation and sustained functionality of neurites. Graphene oxide nanosheets were also incorporated within the scaffold and reduced to form reduced graphene oxide. When used as an additive in polymer composites, reduced graphene oxide increases the presence of polar functional groups at the surface, elevating the surface free energy and resulting in favorable cell interactions.^[^ ^186^ ^]^ In the reduction of graphene oxide, the removal of charge scattering sites in addition to the restoration of п‐bonds within the carbon backbone imparted significant conductivity to the nanosheets, enhancing the regenerative activity of the scaffold.^[^ ^187^ ^]^

#### 6.1.2 Cell‐Laden Nanoengineered Biomaterials Promote Regeneration

Tissues with limited to no regenerative potential such as bone, cartilage, and heart muscle lack the endogenous cell activity to achieve unassisted regeneration of functional tissue. Stem cell‐based regenerative strategies have emerged as a potential approach to address this due to their self‐renewal, multilineage differentiation, and immunomodulatory effects. However, these methods are limited by nonspecific differentiation and low bioretention of exogenous cells. Nanomaterials can be integrated within these systems to enhance differentiation and direct cell bioactivity (Figure 3B).

Cartilage regeneration is difficult to achieve through stem cell‐based methods due to their tendency to form mechanically inferior fibrocartilage.^[^ ^188^ ^]^ By engineering bone marrow mesenchymal stem cells with intracellular copper sulfate nanoparticles, their ability to form natural hyaline cartilage was remarkably improved. The release of copper ions by the nanoparticles activated lysyl oxidase and promoted the synthesis of insulin‐like growth factor‐1, enhancing chondrocyte proliferation and glycosaminoglycan production resulting in articular cartilage formation.^[^ ^189^ ^]^

Similarly, bone regeneration can be achieved through the stimulation of exogenous stem cells by bioactive nanomaterials. Rough titanium nanosurfaces with high surface free energies have shown the ability to drive the differentiation of stem cells toward osteogenic lineages.^[^ ^190^ ^]^ Proteins rapidly adsorb to the high‐energy surfaces upon implantation, as the combination of integrin‐mediated cell‐protein interactions and mechanical stimulus trigger signaling pathways and affect cell morphology.^[^ ^191^ ^]^ 2D titanium carbide nanoflakes significantly enhanced the ability of human periodontal ligament cells to create new bone in rat models in vivo. Indicated by increased RUNX2, HIF‐1α, and β‐catenin, nanoflakes activated the Wnt/β‐catenin signaling pathway to induce bone formation and inhibit osteoclasts.^[^ ^104^ ^]^ After 24 days, ligament cells treated with nanosheets led to a 2‐fold increase in bone volume percentage, new bone area, and bone mineral density in rats.^[^ ^192^ ^]^

Nanomaterials can also enhance regeneration through the stabilization and increased production of biomolecules in physiological environments. Silica‐coated graphene oxide nanosheets were incorporated within gelatin‐methacrylate hydrogels to promote bone morphogenic protein (BMP)‐induced osteogenesis of encapsulated human mesenchymal stem cells. Nanosheets functioned as a reservoir for BMPs by limiting the loss of BMP‐2 and BMP‐7 to surrounding media, creating a positive feedback loop for continued production and retention. Enhancement of the BMP‐SMAD1/5 pathway by the nanoengineered hydrogels significantly increased mineralization in vitro and produced a 385‐fold increase in bone volume in an in vivo mouse model.^[^ ^193^ ^]^ The improved osteoinductive activity of the system can also be partially attributed to the unique wettability of graphene‐based materials. These materials are generally hydrophobic, but this property can be engineered through surface modifications.^[^ ^194^ ^]^ By functionalizing a graphene oxide surface with a thermoresponsive polymer, the ability to switch between a hydrophobic and hydrophilic surface can be achieved. This led to a controlled antibacterial effect on gram‐negative bacteria due to strong hydrophobic interactions at the graphene surface.^[^ ^195^ ^]^ Coating the graphene oxide surface with silica forms a hydrophilic surface, more conducive to cell attachment and proliferation.

Regeneration of cardiac muscle is highly dependent on the ability to reproduce the natural conductivity of the microenvironment. Gold nanoparticles with highly tunable electrical properties have been used to enhance the ability of stem cell‐based treatments to restore heart tissues after myocardial infarction. Brown adipose stem cells and core‐shell gold‐platinum nanoparticles (Au@Pt‐NPs) were loaded within an alginate hydrogel to form a cardiac patch for regeneration. The Au@Pt‐NPs prolonged the survival of encapsulated cells by ROS scavenging through the conversion of H_2_O_2_ to O_2_. The protection of cardiac myocytes under oxidative stress by Au@Pt‐NPs was indicated by the high expression of cx43 and c‐TnT after 28 days of treatment. By improving the propagation of electrical pulses through scar tissue, the nanoengineered hydrogel increased communication between isolated and healthy myocardium to facilitate regeneration.^[^ ^196^ ^]^

In an alternative approach to nanoengineering for cardiac regeneration, conductive phosphorene nanosheets were combined with hydrogen sulfide‐loaded electrospun albumin microfibers to create a cardiac patch. Phosphorene is a layered 2D material, like graphene, but increased interlayer charge distribution leads to a higher polar surface energy component.^[^ ^197^ ^]^ This enhanced wettability is likely the driving force behind the enhanced protein expression by stem cell spheroids incorporated with phosphorene as compared to graphene.^[^ ^198^ ^]^ Primary myocardial cells grown in the cardiac patch showed increased connexin 43 protein expression to cells cultured on fibers alone, suggesting enhanced ability to transmit electrical signals. Thermal energy produced by the nanosheets in response to NIR‐light led to interdigitation of the albumin fibers and imparted tissue adhesive properties to the nanocomposite. Additionally, the inherent angiogenic activity of phosphorene contributed to the regenerative properties of the scaffold. The nanosheets alone showed significant promotion of endothelial cell migration when compared to controls as well as the nanocomposite. In combination with the immunomodulatory effects of hydrogen sulfide, the nanocomposite “band‐aid”‐like structure was able to reduce the size of cavities, restore geometry, and improve the function of the left ventricle in infarcted rat hearts over 28 days.^[^ ^199^ ^]^

#### 6.1.3 Immunoactive Nanomaterials Enhance Regenerative Outcomes

The immune response is initiated by a cascade of proteins that adsorb to the biomaterial surface upon implantation, ultimately determining the regenerative response by the body. Surface characteristics such as chemistry, roughness, or charge dictate which proteins adsorb and ultimately, the type of response generated by the body: pro‐inflammatory or pro‐regenerative. Avoiding chronic inflammation and eliciting a pro‐regenerative response upon implantation is crucial to providing native cells with the proper microenvironment to proliferate and form tissues. Immunoactive nanomaterials improve the host response to biomaterials mainly by directing macrophage polarization and affecting T‐cell activation.

Directing macrophage polarization is a crucial step in driving the immune response. Increasing the population of pro‐regenerative M2 macrophages can be achieved through the sustained delivery of small molecule and protein therapeutics.^[^ ^200^ ^]^ However, the limited half‐life of these materials hinders the ability to maintain therapeutic concentrations over extended periods. Nanoengineered immunoactive polymers are a promising alternative, as these materials can stimulate macrophages and degrade at a controlled rate. An ultrashort peptide with a length of 5 amino acids (Ser‐Glu‐Ser‐Ser‐Glu) was designed to self‐assemble into nanofibers and form an ultrasound‐responsive hydrogel to promote M2 macrophage polarization. The serine‐glutamic acid domain within the peptide nanofibers led to the attenuation of pro‐inflammatory metabolites by macrophages, leading to an increase in M2 macrophages and the stimulation of osteogenesis in bone marrow mesenchymal stem cells in a co‐culture model. Self‐assembly into nanofibers led to twofold increase in bone volume in femoral defect mouse models compared to treatment with non‐assembled peptides.^[^ ^201^ ^]^

T‐cells represent another important cellular immunological component, recognizing and storing information about foreign antigens to provide an adaptive immune response. In autoimmune diseases, uncontrolled T‐cell activation leads to the continuous production of pro‐inflammatory factors. Recently, poly(lactic‐co‐glycolic acid) nanoparticles coated with macrophage membrane were employed as “nanodecoys,” presenting as endogenous cells to avoid clearance. The nanoparticles were able to bind CD4^+^ cell membrane PD‐L1 receptors and inhibit T‐cell activation, alleviating inflammation in both induced arthritic and ulcerative colitic mice models.^[^ ^202^ ^]^

Certain disease states progress by mimicking camouflaging strategies similar to those used by nanomaterials, tricking the body into suppressing inflammatory signals that would otherwise target and clear the antigen. For example, intracellular methicillin‐resistant *Staphylococcus aureus* (MRSA) survives by manipulating macrophage activation toward M2 phenotypes. To address this, a polymeric nanosystem was designed with poly(2‐methacryloyloxyethyl phosphorylcholine) domains that allow specific uptake by macrophages. Upon internalization through scavenger receptor‐B1, the nanovesicles achieve endosomal escape and embedded ferrocene‐containing monomers are oxidized by intracellular H_2_O_2_, causing the release of loaded IFN‐γ, stimulating M1 macrophage polarization. Intracellular oxidation degraded the ferrocene‐containing monomers to Fe^2+^, causing Fenton reactions that produced ROS and further enhanced the inflammatory response. In mice injected with MRSA‐infected macrophages, the polymeric nanovesicles cleared the bacteria within 8 days, faster than free IFN‐γ and untreated controls in the same period.^[^ ^203^ ^]^ The levels of MRSA remaining in mice after 6 days of treatment decreased by 3 orders of magnitude relative to Van, the commercial standard of care.

### 6.2 Overcoming Biological Barriers for Successful Drug Delivery

Drug delivery to the target site remains a challenge due to numerous intracellular and biochemical barriers encountered in vivo. Nanotherapeutics have subsequently gained tremendous interest in overcoming many of these obstacles encountered in the successful delivery of small and large molecules. In the case of small molecules, the major applications revolve around improving the solubility and circulation time, modulating release profiles, and achieving site‐specific delivery. As for large molecules, the focus of research has been on stabilization, improving delivery efficiency, and minimizing undesirable side effects. The rational design of nanotherapeutics involves careful consideration of the payload properties and nanoparticle design strategies to collectively overcome issues encountered.

#### 6.2.1 Structural Nanoengineering Enables Small Molecule Delivery

**Figure 4:**
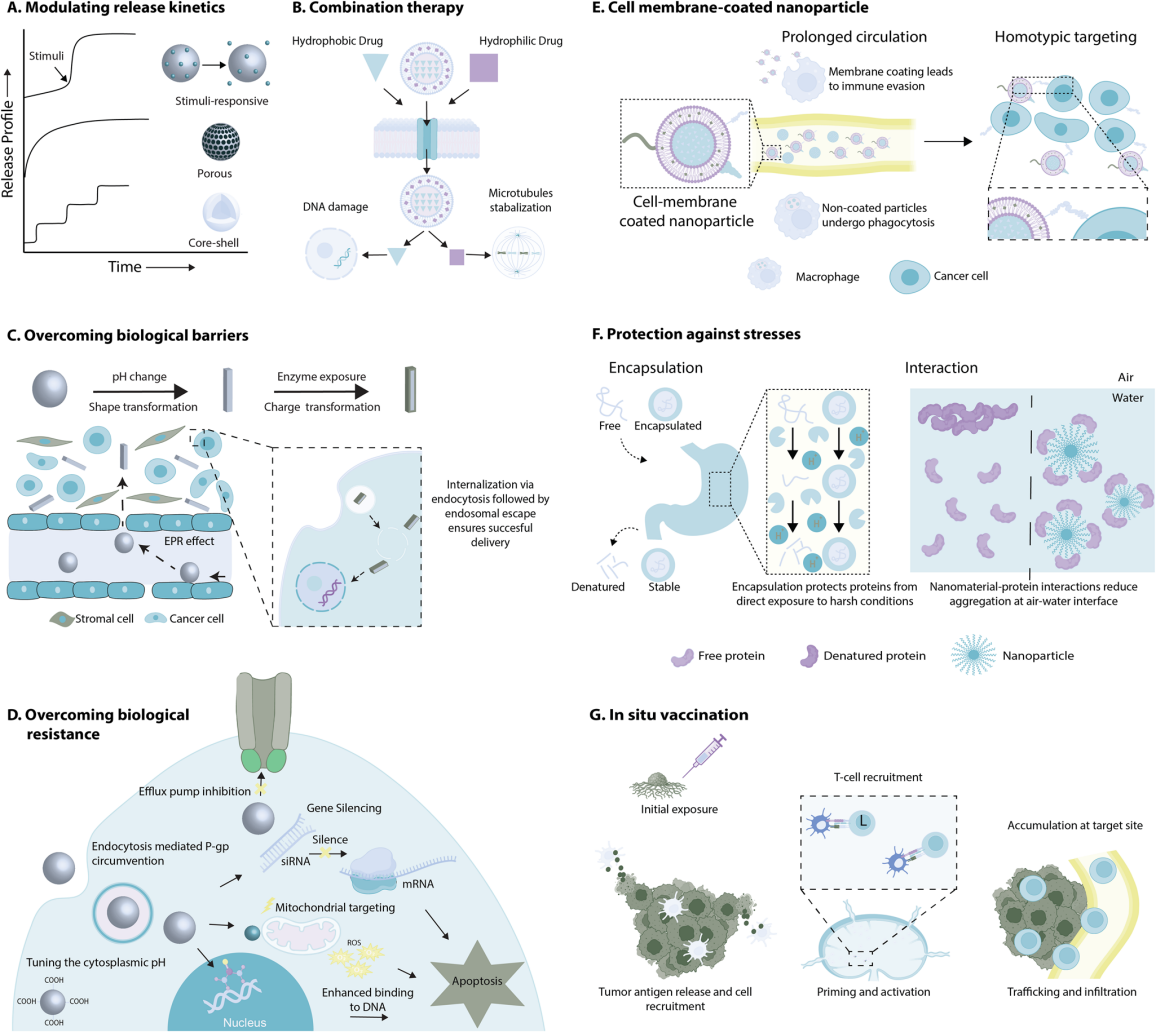
Designing nanoparticles for successful delivery of small molecules and biologics. A) The structural engineering of nanoparticles—including their architecture, composition, and surface characteristics—allows for precise control over their behavior in vivo. B) Amphiphilic nanomaterials can be utilized for combination therapies via simultaneous deliveries of hydrophobic and hydrophilic molecules. C) Nanoengineered biomaterials can be designed to overcome biological barriers and perform intracellular therapeutic release. D) Avoiding clearance through endocytosis‐mediated P‐glycoprotein (P‐gp) circumvention reduces multidrug resistance in cancer cells. E) Coating nanomaterial surfaces with cell membranes leads to immune evasion and allows for increased local delivery of biologic agents. F) By employing encapsulation and chemical stabilization strategies, nanoparticles can protect biologics from protein denaturation, thereby maintaining their efficacy. G) Nanomaterials are also being explored as the delivery vehicles for tumor‐targeting agents, adjuvants, and cytokines, and for the capture of tumor antigens within the tumor microenvironment. The figure was created in Adobe Illustrator using the icons from BioRender.com.

Nanomaterials have demonstrated utility for increasing circulation time, reducing efflux pump‐mediated drug resistance, combination therapy, enhancing drug accumulation in target tissues, and achieving controlled release (**Figure** **4**). Extensive studies have focused toward improving the residence time of small molecules through their encapsulation into nanocarriers. PEGylation is an established method of surface coating to achieve steric stabilization and stealth properties.^[^ ^204^ ^]^ Early studies indicated that Doxil, an FDA‐approved PEGylated liposomal formulation of doxorubicin, extended the half‐life of the free drug in the human body by over 100 times.^[^ ^205^ ^]^

The field has since progressed, and more recently sophisticated techniques have been explored to enhance the in vivo residence times of drug‐loaded nanomaterials to achieve prolonged action. These include coating nanoparticles with natural cell membranes as well as genetic engineering approaches.^[^ ^206^ ^]^ Extensive work has been done on coating nanomaterials with red blood cell membranes, as this imparts self‐markers such as CD47 proteins, peptides, glycans, and acidic sialyl moieties, allowing nanoparticles to avoid immune recognition.^[^ ^206^, ^207^ ^]^ In a recent study, genetic engineering of the macrophages used for coating PLGA nanoparticles to express proline‐alanine‐serine (PAS) peptide chains resulted in a 1.7‐fold increase in residence time.^[^ ^208^ ^]^ Due to the high solubility and lack of charge conferred by the PAS polypeptides, these genetically modified nanoparticles demonstrate enhanced circulation times and reduced immune response.

Zwitterionic polymers, owing to their neutral charge and robust electrostatically driven hydration, are increasingly being utilized to prolong the systemic circulation of nanoparticles by virtue of reduced non‐specific protein adsorption and opsonization.^[^ ^209^ ^]^ A recent study focused on designing zwitterionic nanoparticles demonstrated outstanding resistance to nonspecific protein adsorption and circulation time close to 100 hours.^[^ ^210^ ^]^ The relatively low surface energy associated with zwitterionic nanoparticles also results in minimizing protein adsorption and immune recognition, leading to prolonged circulation times in the bloodstream.

Apart from improving the half‐life, efforts have also been directed towards modulating the release properties of these nanocarriers to achieve pulsatile release, stimuli‐triggered release, and avoiding burst release (Figure 4A,B). These efforts have focused on engineering nanoparticles by modifying the architecture, composition, and loading techniques of the nanoparticles.^[^ ^211^ ^]^ Layered nanoparticles represent one such design strategy that provides flexibility to fine‐tune the desired properties of the nanocarrier by modulating different layer properties.^[^ ^212^ ^]^ Multilayered gold nanostructures were recently designed for controlled and simultaneous delivery of two chemotherapeutics, demonstrating tunable drug release kinetics through architectural control over the number of layers. Such systems also find utility in self‐boosting immunization approaches wherein by controlling the lag phase period, the latter antigen exposure can be tuned. In an interesting study investigating the effect of phase composition on the bioactivity of calcium phosphate nanoparticles, it was noted that through incorporating different calcium phosphate phases in various proportions, drug release profiles could be tailored hours to years.^[^ ^213^ ^]^ They explored the use of various calcium phosphate phases, and as anticipated, the release rate of model drugs was closely linked to the degradation rate of each phase. This demonstrated the potential to tune drug release from a few hours—using highly soluble phases like monocalcium phosphate—to several years with less soluble ones like hydroxyapatite.

Utilizing structural cues in the nanoparticle to provide site‐specific release has also been an actively researched field. To this end, a shell‐by‐shell, redox stimuli‐responsive release strategy was recently employed to reverse the tumor immunosuppressive microenvironment through simultaneous delivery of anticancer agents and CRISPR/Cas9.^[^ ^213^ ^]^ The system demonstrated successful control over the inhibition of lactate dehydrogenase A and the mitochondrial tricarboxylic acid cycle.

A vast majority of nanoengineered drug delivery research has been directed toward improving the targeted site localization through passive and active targeting (Figure 4C,D).^[^ ^214^ ^]^ Passive targeting incorporates materials that respond to stimuli including pH, temperature, or enzymatic activity as cues in the nanomaterial design. Active targeting focuses on a targeting molecule that binds to a tumor‐specific antigen or receptor; the system is expected to enhance drug accumulation at the tumor site. In a recent study focused on achieving spatial‐specific drug release, a multi‐responsive self‐assembled nanodrug delivery system was developed to deliver dipyridamole. This system is composed of anti‐diabetic metformin bound to an anticancer agent by a matrix metalloproteinase‐2 (MMP‐2) responsive peptide.^[^ ^215^ ^]^ These nanoparticles targeted tumors using the enhanced permeability and retention effect. At the tumor site, MMP‐2 cleaved the peptide, initiating three separate actions by the nanoparticles; platelet binding by dipyridamole, metformin release to downregulate PD‐L1 and repolarize M2 macrophages, and targeting the integrin αvβ3 receptor for precise tumor cell delivery and killing. Recently, an interesting study looked at coating self‐assembled particles coated with glioma cancer cell membranes modified with a tumor‐targeting folate (FA) ligand, enabling them to act as a “disguiser” for enhanced tumor specificity.^[^ ^216^ ^]^ The particles exhibited outstanding tumor‐targeting abilities due to the specific FA ligand and homologous targeting function of GCM, showing potential for glioma therapy.

#### 6.2.2 Leveraging Nanoengineering for Enhanced Stability of Biological Agents

The high specificity and potency associated with different biological agents open them for use in wide‐ranging disorders. However, the associated instability and immunogenicity limit their clinical use. The design of nanoparticles capable of achieving site‐specific and controlled delivery of different peptides, proteins, antibodies, DNA, and RNA therapy while ensuring their stability is required.^[^ ^217^ ^]^ Nanoengineering improves biological delivery through stabilization and enhanced cellular uptake.

The clinical failure of many commonly utilized polymeric nanotherapeutics highlights the need to reexamine and investigate newer design strategies for overcoming biological barriers. One such strategy that has received major interest in the last two decades is biomimetic drug delivery systems. Taking inspiration from different pathogens around the mechanisms to evade the host immune system, extensive work has been done in designing improved drug delivery systems.^[^ ^218^ ^]^ To this end, a pH‐responsive biomimetic metal‐organic framework delivery system was developed for plasmid and nanozyme co‐delivery to treat ulcerative colitis. In lieu of improving the targeting ability, the system was coated with macrophage membranes. At the site of action, the nanozyme effectively scavenged reactive oxygen species and modulated the inflammatory microenvironment, while the CD98‐targeting CRISPR/Cas9 plasmids alleviated inflammation by suppressing CD98 gene expression at the genomic level.^[^ ^219^ ^]^ Inspired by the ability of enteropathogenic bacteria to adhere to intestinal epithelia and modulate tight junction interactions, UEA I anchored NPs engineered nanoparticles encapsulating bacterial fragments to target claudin‐4 were recently used for paracellular delivery.^[^ ^220^ ^]^ The UEA I surface functionalization allowed for apical glycocalyx attachment while the bacterial fragments ensured transient tight junction opening.

Due to the potential genotoxicity of nucleic acid‐based therapeutics upon off‐target delivery, spatiotemporal delivery is critical. Therefore, significant research efforts have been directed toward precise control and synthesis of stimuli‐responsive polymeric nanomaterials for local delivery (Figure 4E). A recent study aimed at engineering amphiphilic poly(β‐Amino ester) nanoparticles for lung endothelial cells delivery of non‐integrating DNA plasmids and precise control of transfection efficiency. By structurally controlling the ratio of two alkyl chains in the backbones, length of the fluorous ligands, and addition of PEG in the end cap, lung endothelial cell targeting, and high transfection were achieved. The fluoridation of these nanoparticles helped stabilize the polymer chains in the nanocomplexes, improved cell uptake, and facilitated hydrogen bonding interactions with glycocalyx in the lung.^[^ ^221^ ^]^ The positive surface charge of the nanoparticles also ensured better contact with negatively charged glycocalyx on surface of endothelial cells.

Non‐invasive delivery of biologics remains a significant challenge on account of the numerous intracellular, biochemical, and physiological barriers encountered during the transit.^[^ ^222^ ^]^ This is another domain where nanoengineered biomaterials have demonstrated usage. Recently, structural engineering approaches used to design a pH‐sensitive self‐unpacking capsule containing metal‐organic framework nanoparticles coated with a zwitterionic hydrogel for oral peptide delivery showed great promise.^[^ ^223^ ^]^ These systems were shown to successfully overcome the mucosal and acidic barriers and successfully delivered exendin‐4 for extended duration (Figure 4F). Recent studies have also focused on integrating these nanocarriers in delivery vehicles such as hydrogel dressings or biomimetic scaffolds wherein they can be used toward combination therapy.^[^ ^224^ ^]^

The limited success of cancer vaccines due to tumor antigen variability and logistical issues with identifying and delivering tumor antigens has resulted in other strategies being actively explored.^[^ ^225^ ^]^ One such strategy that has since then gained tremendous interest is in situ vaccines, which take advantage of the tumor‐associated antigens present at the tumor site to elicit potent cytotoxic T lymphocyte responses (Figure 4G). To this end, a recent study used mannan conjugated acid‐responsive liposome‐coated polydopamine nanoparticles. Interestingly, tumor site localization was achieved through liposome decomposition, while the nanoparticles facilitated the photothermal therapy and immunogenic cell‐death‐induced tumor‐associated antigens capture. The mannose‐modified nanoparticles target dendritic cells, releasing R848 to activate antigen presentation. Consequently, this in situ vaccine not only effectively matures dendritic cells but also significantly enhances their impact on cytotoxic T lymphocyte cells.^[^ ^226^ ^]^

### 6.3 Nanoengineering Imparts Dynamicity to 3D‐Printed Biomaterials

**Figure 5:**
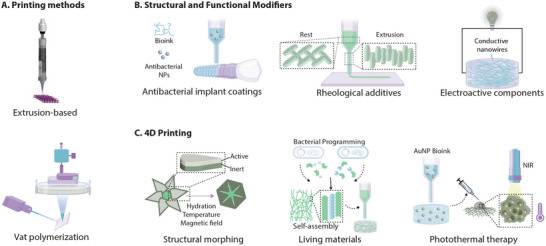
Nanoengineering dynamic biomaterials with additive manufacturing. A) Extrusion‐based printing and vat polymerization are the two main modalities of additive manufacturing utilized to fabricate nanoengineered biomaterials. B) The incorporation of nanomaterials into conventional inks imparts bioactivity, tuned printability, and additional functionalities. C) Nanoengineering allows for 4D printing, where time behaves as an additional dimension where properties vary. In response to changes in hydration levels, temperature, magnetic fields, or other external stimuli, the structure and properties of smart nanocomposites undergo corresponding changes. Engineered living materials can be generated by programming microbes to release self‐assembling bioactive molecules that are combined with conventional bioinks. Stimuli‐responsive nanomaterials can also be incorporated within traditional printing materials to achieve printable smart materials. The figure was created in Adobe Illustrator using the icons from BioRender.com.

Additive manufacturing has evolved from 3D printing for prototyping devices, to the inclusion of electronic components, to printing bioactive materials with favorable cell interactions. Different modalities such as extrusion‐based or vat polymerization have been applied to fabricate complex hierarchical structures with high biocompatibility (**Figure** **5**A). Nanomaterials can enhance printing outcomes by providing precise control over structural features, mechanical properties, and stimuli‐responsiveness.

#### 6.3.1 Structural and Functional Modifications via Nanoengineering

Nanoengineering in 3D printing imparts a dimension of tunability to printed structures as their adhesive properties, biodegradation, or antibacterial properties can be affected in a concentration‐dependent manner. Nanomaterials can be incorporated within the bulk to achieve systematic effects on material properties while nanoengineering at the surface imparts local biophysical cues to biological materials (Figure 5B).^[^ ^227^ ^]^ For example, a biomimetic scaffold was fabricated through the incorporation of Strontium ions and dimethyloxalylglycine‐loaded mesoporous silica nanoparticles in gelatin nanofibers. The nanofibers were then embedded within the pores of an extruded PCL scaffold for bone regeneration. The nanoengineered scaffolds promoted angiogenesis and osteogenesis through the HIF‐1α pathway. Scaffolds including nanofibers without loaded nanoparticles led to a 2‐fold increase in bone mineral density over 8 weeks, which was further enhanced by the addition of nanoparticles.^[^ ^228^ ^]^ Control over the degradation rate and kinetics of co‐delivery were controlled through the density of nanofibers. Similarly, zinc oxide nanoparticles were incorporated within a polyvinylidene fluoride (PVDF) wound dressing to improve its piezoelectricity and antibacterial activity.

Zinc oxide particles increased the crystalline polarization of PVDF toward its β phase which controls its piezoelectric properties. This polarization directly improved the piezoelectric activity of the wound dressing. Additionally, the inherent antimicrobial properties of zinc oxide nanoparticles enhanced the antibacterial activity of the wound dressing against *Escherichia coli, Staphylococcus aureus*, and methicillin‐resistant *Staphylococcus aureus*.^[^ ^229^ ^]^ The addition of zinc oxide nanoparticles increases the overall surface energy, but lowers the polar component, resulting in hydrophobicity and reduced bacterial adhesion and survival.^[^ ^230^ ^]^

The use of smart materials such as bioinks allows for 4D printing, in which time acts as an additional dimension where structural and functional changes can occur. This can be used to print adaptable structures for soft robotics and tissue engineering. Responsive nanomaterials are often leveraged to achieve 4D‐printed structures change over time in response to external stimuli.^[^ ^231^ ^]^ Reduced graphene oxide nanosheets have photothermal properties that have been utilized to control the actuation of a soft hydrogel robot. The nanoengineered hydrogel was stimulated by NIR light to perform actions such as grasping and lifting objects through the production of thermal energy.^[^ ^232^ ^]^ Similarly, photothermal gold nanoparticles controlled the shape memory behavior of a polyurethane matrix. Heat generated by the embedded nanoparticles allowed for in situ expansion capabilities and photothermal tumor ablation.^[^ ^233^ ^]^ Apart from photothermal properties, 4D printing can also be achieved with magnetic nanoparticles. Ferromagnetic iron oxide nanoparticles specifically patterned within soft hydrogels result in soft actuators that perform precise, complex movements in response to external magnetic fields.^[^ ^234^ ^]^

#### 6.3.2 Nanomodifiers Enhance Cytocompatibility of Bioprinted Structures

Bioprinting involves the combination of biological components to form a bioink and recreate a desired tissue or organ.^[^ ^235^ ^]^ This requires a biomimetic, cell‐friendly microenvironment to replicate physiological phenomena. Nanomaterials are added to bioinks to improve material‐living system interactions and preserve cell viability (Figure 5B). In an alginate and gelatin‐based bioink, copper‐doped bioactive glass nanoparticles introduced cell‐adhesive ligands that led to the rapid spreading of embedded osteosarcoma cells and mouse bone marrow mesenchymal stem cells. Copper ions released by the nanoparticles contributed to angiogenesis indicated by the increased intracellular production of VEGF.^[^ ^236^ ^]^ Research has also shown that nanosilicates can modulate the shear‐thinning properties of bioinks and support printability even at high cell densities of up to 10 million cells/mL. At rest, laponite discs are oriented in a house‐of‐cards structure due to charge interactions between anionic faces and cationic edges. During extrusion, these nanosilicate discs orient parallel to the direction of flow, resulting in shear‐thinning properties on the macroscale. Nanosilicates improved the resolution of a gelatin‐based bioink, allowing for the direct fabrication of free‐standing microvessels to model vasculature.^[^ ^237^ ^]^

Smart nanomaterials can also be added to bioinks to achieve 4D bioprinting (Figure 5C). The stimuli‐responsiveness achieved through nanoengineering allows for the modulation of cellular processes within printed constructs, forming “living materials” with dynamic responses to external stimuli. For example, *E. coli* was genetically engineered to form a chemically crosslinked bioink from self‐assembling nanofibers. Based on fibrin‐derived protein domains, the nanofibers formed supramolecular assemblies through knob–hole interactions. Through separate methods to induce the nanofibers, the bioink could be stimulated to synthesize anticancer therapeutics, sequester specific biomolecules in solution, or regulate the growth of encapsulated cells.^[^ ^238^ ^]^

### 6.4 Other Emerging Applications of Nanoengineered Biomaterials

#### 6.4.1 Toward Closed‐Loop Devices through Nanoengineered Bioimaging

**Figure 6:**
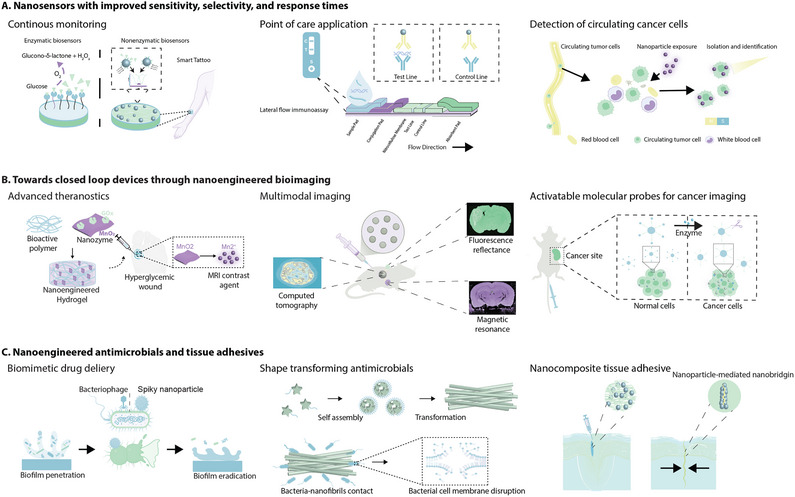
Engineered nanoparticles open newer avenues in sensing, imaging, and antimicrobial therapy. A) The inherent unique optical, efficient target capture and signal generation abilities as well as superparamagnetic properties associated with nanoengineered biomaterials facilitate their integration into non‐enzymatic biosensors, immunoassays, and for cancer cell detection, respectively. B) Engineering nanoparticles through surface functionalization with ligands capable of specific biomarker targeting ability and distinct imaging functionalities results in enhanced targeting and complementary functions. Nanoparticles also provide opportunities for functional integration through combined diagnostic and therapeutic functions, enabling theranostic applications in closed‐loop devices. C) Incorporating unique structural features such as sharp edges, high surface area, and environmental responsiveness can improve bacterial cell membrane disruption, increase adhesion, and facilitate better targeting. The incorporation of nanoparticles reinforces the adhesive matrix, providing better structural integrity and higher surface area for stronger adhesion to tissue surfaces. The figure was created in Adobe Illustrator using the icons from BioRender.com.

Bioimaging is crucial for the early detection and treatment of diseases. However, physiological barriers frequently lead to reduced efficacy of synthetic imaging probes in living systems. Nanomaterial‐based fluorescence probes with superior sensitivity, selectivity to the target, adequate dispersibility, high stability, resistance to photobleaching, and good contrast properties are necessary to address these limitations (**Figure** **6**A).^[^ ^239^ ^]^ Based on the fluorescence properties of these nanomaterials, their applications for live cell imaging have been divided into three categories. Type 1 and 2 were designated to nanomaterials with intrinsic fluorescence and fluorescence‐quenching properties, respectively. These materials were reported to be used for live‐cell imaging. The non‐quenching, non‐fluorescent nanomaterials, referred to as Type 3, were described as a platform for either immobilizing fluorescent probes on their surface or encapsulating fluorescent dyes within their core.^[^ ^240^ ^]^

The multifunctionality associated with many nanomaterials warrants their incorporation in closed‐loop devices for simultaneous diagnostic and delivery use. To this end, nanocomposite self‐healing hyaluronic acid hydrogels loaded with nanoenzymes and microbubbles demonstrated use for simultaneous diabetes diagnosis and treatment.^[^ ^12^ ^]^ The catalytic ability of graphene oxide‐manganese dioxide nanoenzymes demonstrated its utility in glucose detection and interestingly the manganese ions generated in situ showed potential in improving magnetic resonance imaging. Recently, multifunctional metal–organic nanocages were designed for synergistic photodynamic therapy through tumor‐targeting, drug transport, and cancer cell tracking.^[^ ^241^ ^]^ By structurally engineering the MOF the authors were able to make use of the high‐atomic number Os‐element to interact with X‐ray irradiation for dual radiosensitization and photosensitization, use the intrinsic NIR emission properties, and improve solubility for anticancer drug delivery. They successfully demonstrated the use of collaborative cancer theranostics on a single‐nanomaterial platform. Significant work has also been done in the field of drug‐resistant bacterial infections. Recently, bomb‐inspired self‐assembling nanospheres with aggregation‐induced emission were designed.^[^ ^242^ ^]^ These nanospheres demonstrated bacterial site specificity and showed rapid drug release in addition to excellent photothermal and photodynamic properties in vivo.

Nanomaterials are also employed to specifically target cancer site biomarkers, achieving real‐time drug monitoring at cancer sites. This becomes increasingly important due to the spatiotemporal variation and heterogeneities associated with the growth of different tumors, which frequently dictates the treatment sensitivity and outcome. Additionally, the use of responsive imaging probes can improve target‐to‐background ratio for improved sensitivity and specificity. Furthermore, the performance of these imaging probes can be further enhanced by carefully tuning the surface energy for desired stability, dispersion, and targeting efficiency. In a recent study, caspase‐3‐based fluorescence self‐monitoring was achieved through the use of graphene oxide‐based nanomaterials conjugated with fluorescein‐labeled caspase‐3‐specific substrate peptide.^[^ ^243^ ^]^ Toward the same goal, gold nanocluster‐decorated MOFs were also investigated.^[^ ^244^ ^]^ Enzymatic cleavage of the peptide linker between nanoclusters and MOFs led to fluorescence in HepG2 cells that were used to keep track of delivery. Dual‐mode therapeutic evaluation was achieved by simultaneously detecting released gold ions by inductively coupled plasma‐mass spectrometry.

Increasing focus is also being directed toward achieving multimodal imaging with nanoparticles merging the properties of two or more imaging modalities. These multimodal‐imaging probes not only help in achieving precise diagnosis but also monitor disease progression. Efforts have been directed towards engineering the nanoparticle architecture through control of inner and outer shell thicknesses in core‐shell nanoparticles to ensure optimal activity for the multimodal image probe while avoiding quenching effects often resulting in sub‐optimal properties of upconverting nanoparticles‐based probes.^[^ ^245^ ^]^ Efforts have also been focused toward designing semiconductor‐based heteronanocomposites to combine the enhanced hydroxyl radical production of copper‐containing materials in the tumor microenvironment with the high photothermal performance of molybdenum disulfide.^[^ ^246^ ^]^ As expected, the system displayed a synergistic effect while also enabling trimodal imaging—CT, IR thermal, and MRI—owing to the excellent X‐ray attenuation of molybdenum, the strong NIR absorbance of molybdenum disulfide‐copper oxide, and the paramagnetic nature of copper(II). The platform showed clear T1‐weighted MRI and CT contrast in both in vitro and in vivo settings, along with efficient infrared thermal imaging driven by robust photothermal conversion and significant localized heating at tumor sites.

By introducing controlled defects, such as nitrogen‐ or oxygen‐related vacancies, and manipulating the crystalline lattice, challenges in the development of NIR‐II fluorescence imaging can be addressed. In the past few years, the interest in NIR‐II fluorescence imaging has risen, driven by the high temporal resolution, high spatial resolution, low scattering, and strong penetrability of deep tissue, however efficient bandgap control remains a challenge.^[^ ^247^ ^]^ A recent study employed an oxidizing agent to introduce nitrogen‐ and oxygen‐related vacancies, inducing lattice distortions that alter electronic energy levels and enhance electron–phonon coupling, thereby facilitating polaron formation.^[^ ^248^ ^]^ Nitrogen‐related vacancies gave rise to non‐radiative polarons that enhanced NIR‐II absorption, enabling significant photothermal energy conversion, while oxygen‐associated defects facilitated radiative recombination, producing bright fluorescence with a peak emission at 1300 nm. The engineered carbon quantum dots were successfully used for “in vivo NIR‐II imaging,” clearly visualizing blood vessels and tumors in mice with a high signal‐to‐noise ratio and demonstrated “effective photothermal therapy,” achieving tumor ablation with minimal skin damage. In another study, by utilizing the Ag_2_S‐seed mediated synthesis, authors reported that an unusual orthorhombic crystalline phase imparted good emission properties.^[^ ^249^ ^]^ This was validated through comparing their ability to image the complex vascular system to commercial Ag_2_S nanoparticles.

#### 6.4.2 Nanosensors with Improved Sensitivity, Selectivity, and Short Response Times

Ever since first ideated by Clark and Lyons for glucose monitoring, the use of biosensors has expanded to applications in intra‐ and inter‐cellular signaling pathways, environmental sensing, and the continuous tracking of the spatio‐temporal dynamics of specific analytes.^[^ ^238^ ^]^ While a lot of the early work for glucose monitoring revolved around glucose oxidase enzyme immobilization and improving sensitivity, recent research has focused also on trying to identify non‐enzymatic approaches to overcome stabilization and microenvironment dependence issues encountered previously.^[^ ^250^ ^]^ These efforts have resulted in looking at noble metals, metal alloys, graphene, and metal nanoparticles as possible electrode materials.^[^ ^251^ ^]^ Nanomaterials can lower the detection limits of biosensors through improving the assay sensitivity, shortening response time, and increasing selectivity (Figure 6A). There have also been active research surrounding optimizing the nanomaterial properties for improving efficiency use in sensing, much of this has focused on modulating their surface properties through functionalization with luminescent tags and surface coatings.^[^ ^252^ ^]^

Metal nanoparticles have been extensively applied as nanobiosensors due to their ability to reduce response times and improve sensitivities by preconcentrating analytes before applying them to the sensing interface.^[^ ^253^ ^]^ Furthermore, opportunities exist toward combining them with different signal amplification strategies to further improve sensitivity. Recently, Fe_3_O_4_–Au core–shell nanoparticles were coupled with another signal amplification strategy to achieve highly dispersible and magnetically responsive nanoparticles for the detection of circulating tumor DNA. This research successfully combined the core‐shell nanoparticles with polymerase chain reactions to achieve localized genetic amplification on the nanoparticle surface. The authors subsequently demonstrated attomolar sensitivity and rapid detection of metastatic breast cancer circulating tumor DNA in vitro.^[^ ^254^ ^]^ It also becomes important to consider the innate structural features of different nanoparticles which can be leveraged to further improve sensitivity. The small measurement volume associated with a nanopore biosensor ensures that a significant change in resistance occurs even when a single analyte molecule passes through the nanopore hence ensuring sensitivity.^[^ ^255^ ^]^

The increased area of the space charge region and efficient separation of electron–hole pairs associated with 2D semiconductors has also resulted in their growing popularity in photoelectrochemical biosensors. Research in this field has primarily focused on optimizing excited electron generation, introducing specific materials to create new electron transfer pathways, and improving the separation of excited electrons.^[^ ^256^ ^]^ A recent study investigated the use of doping, heterojunction formation, and polarization modulation to synergistically optimize the generation and separation of excited electrons in photoelectrochemical materials. This resulted in the fabrication of a self‐powered biosensor for carcinoembryonic antigens with a low detection limit of 1.91 pg mL^−1^. The biosensor employs neodymium doping in bismuth ferrite to enhance its ferroelectric properties, improving charge separation and photocurrent. A heterojunction formed between bismuth vanadate and neodymium‐doped bismuth ferrite creates a built‐in electric field, facilitating efficient electron–hole pair separation. This combination enhances light absorption and charge transfer, boosting the photocurrent. Modulating the polarization further increases the photocurrent while maintaining stability under long‐term irradiation. In situ sensitization has gained some interest as a new electron transfer pathway, wherein dynamically modifying the electrode surface can improve charge separation efficiency and light absorption. This is often achieved through small‐molecule probes or enzymatic reactions. In a recent study, a small‐molecule silane probe functionalized onto graphitic carbon nitride results in the formation of silicon nanoparticles at the electrode surface.^[^ ^257^ ^]^ This leads to the formation of a heterojunction between the silicon particles and graphitic carbon nitride, which establishes a new electron transfer pathway, improving light absorption and electron separation efficiency. To this end, a hybrid electron extraction system based on platinum nanoclusters integrated with iron single‐atom catalysts was recently employed toward enabling efficient carrier extraction while accelerating the oxygen reduction reaction through forming a charge transfer pathway.^[^ ^258^ ^]^

Engineering these metal nanoparticles can also result in further increased selectivity. To achieve this, a single‐step periostin sensing platform was developed by concurrently employing two periostin‐specific ssDNA aptamers along with gold and silver nanoparticles, resulting in a low detection limit of 106.68 pM in buffer and 463.3 pM under serum‐spiked conditions.^[^ ^259^ ^]^ The use of both aptamers allows the nanoprobes to remain stably dispersed in solution while providing strong specificity toward periostin.^[^ ^259^ ^]^ Additionally, the core‐satellite structures generated upon binding of analyte resulted in increasing metal enhanced fluorescence effect and amplification of the overall fluorescence signal by facilitating efficient energy transfer.

The rapid isolation, easy manipulation, and high capture performance associated with immunomagnetic nanoparticles has resulted in them being actively explored for detection of circulating tumor cells. However, their clinical translation remains limited owing to the heterogeneous nature of tumors and nonspecific leukocyte adsorption. To this end, membrane‐coated magnetic nanoparticles have also found use as isolation medium wherein recently membrane coated magnetic nanoparticles demonstrating overexpressed scFv of the anti‐epidermal growth factor receptor (EGFR) antibody on the membrane surface were used.^[^ ^260^ ^]^ This enabled high efficiency in specific recognition of EGFR‐positive circulating tumor cells and their homology enabled repulsion upon encounter of leukocytes thereby reducing nonspecific adsorption.

Very recently there has been a tremendous interest in using the clustered regularly interspaced short palindromic repeats (CRISPR)‐associated nuclease (Cas) (CRISPR/Cas) systems in molecular diagnostics.^[^ ^261^ ^]^ This can be credited to the exceptional molecular target recognition specificity, rapid response time, simple isothermal reaction conditions, and effective signal amplification features.

#### 6.4.3 Enhancing Antimicrobial Effects Through Inherent Bioactivity

The emergence of antibiotic‐resistant pathogens over the past few decades has resulted in major research efforts towards the design of effective antimicrobial biomaterials (Figure 6B).^[^ ^262^ ^]^ Metallic nanoparticles such as gold, copper, and silver have been investigated to address this due to their biocompatibility and inherent antimicrobial properties.^[^ ^263^ ^]^ Electrostatic attraction between positively charged nanoparticles and the negatively charged bacterial cell wall can lead to membrane disruption, compromising the integrity of the bacterial cells. This interferes with vital processes such as respiration, nutrient uptake, and DNA replication. Oxidative stress via reactive oxygen species generation and free metal ion toxicity are also hypothesized to contribute to the mechanism of bactericidal activity.^[^ ^264^ ^]^ This provides an opportunity to engineer nanoparticles that can target specific Gram‐negative bacteria.^[^ ^265^ ^]^ However, freely administered nanomaterials are subject to rapid clearance via renal filtration or phagocytosis. In some cases, nanoparticles themselves can be used in undergoing chemical reactions to form more stable structures. Gold nanoparticles can form coordination bonds with thiolated polymers leading to the formation of crosslinked injectable networks.^[^ ^266^ ^]^ Recently, a copper MOF was chemically and ionically dual‐crosslinked to an alginate hydrogel to be utilized as an antibacterial agent.^[^ ^267^ ^]^

The reduced resistance associated with nanoparticles is driving major research efforts towards utilizing them for use in bacterial infections associated with acquired antibiotic resistance and biofilm. To this end, several structural engineering approaches are being leveraged to repurpose nanoparticles for synergistic treatments. In lieu of the enhanced localized bacterial capture and penetration of the cell envelope associated with bacteriophages, bioinspired spiky nanospheres have been recently designed.^[^ ^268^ ^]^ These nanospheres are able to take advantage of their topological features for deep lipid membrane penetration and can be tuned towards enhancing ROS‐catalytic activity. The authors reported engineering Fe−O−Mo‐based synergistic dual‐atom sites with strong metal−support interactions to enhance intrinsic ROS‐catalytic performance. Another interesting attempt towards improving penetration through Gram‐negative bacterial outer membrane has focused on utilizing shape‐transforming self‐assembled nanostructures capable of mechanically disrupting the bacterial envelope towards improved treatment outcomes.^[^ ^269^ ^]^ The authors reported these engineered nanofibrils are capable of disrupting metabolic pathways; activate innate and adaptive immunity among others. Interestingly, the nanofibrils stiffness was observed to have a major influence on its membrane disruption ability. Such approaches highlight the need for sophisticated nanoparticle design toward overcoming major global healthcare concerns.

Implant‐associated infections can lead to inflammation and the disruption of tissue function. When applied as implant coatings, antibacterial nanoengineered materials address this through one of two major approaches: biofilm inhibition and contact killing (Figure 6C).^[^ ^270^ ^]^ Nanoparticles coated with bacterial membranes were designed to compete with the original bacteria for binding sites, thereby preventing bacterial attachment to host cells. They were able to demonstrate that treatment of the gastric epithelial cells with these bacterial outer membranes coated nanoparticles resulted in reduced attachment and even drove detachment of already bound pathogenic bacteria.^[^ ^271^ ^]^ In another such attempt, functionalized iron oxide nanoparticles with silk fibroin deposited on a titanium substrate reduced the surface bacterial adhesion of methicillin‐resistant *Staphylococcus aureus* and *Escherichia coli* more than 100‐fold compared to a bare titanium surface.^[^ ^272^ ^]^

Contact killing involves the rapid adsorption and death of micro‐organisms that adhere to a surface. Amongst the different materials being explored for contact‐killing, quaternary ammonium salts are of great interest owing to their broad‐spectrum antibacterial activity. In a recent study, amphiphilic nanoengineered polyquaterniums were synthesized to promote bacterial phagocytosis and exhibit use in preventing microbial infections. Their strong charge‐positive property and rapid adsorption of bacteria after 10 seconds of contact led to the death of more than 99% of pathogens within 30 seconds.^[^ ^273^ ^]^ These contact‐killing properties also extend to different metals and metal oxides‐based nanoparticles.^[^ ^274^ ^]^

#### 6.4.4 Nanofeatures Facilitate Material–Matrix Interactions for Tissue Adhesion

In wound repair, tissue adhesives offer various advantages over alternatives such as sutures or staples. Ease of implementation, rapid sealing, and reduced patient discomfort have led to a surge in clinical demand for and the subsequent development of soft tissue adhesives based on natural and synthetic polymers. Conventional polymer‐based adhesives typically lack the mechanical strength and adhesive properties required to achieve sufficient wound closure. Nanomaterials can be added within these systems to improve these aspects and impart desired properties (Figure 6C). For example, laponite discs were added to a gelatin‐based skin scaffold to improve its tissue adhesion, cell‐material interactions, and hemostatic ability. By adding a small amount of laponite (1 w/v%) to gelatin methacrylate, the adhesive strength of the hydrogels increased nearly threefold. The anionic surface of laponite discs provided increased binding sites for cells, causing the population of cells attached to the scaffold to double within 5 days after seeding, compared to the unchanged population in the control group. The negative charge also led to improved clotting ability by increasing the concentration of clotting factors near the scaffold surface.^[^ ^275^ ^]^

Wearable bioelectronics also require adhesive properties, as these devices must interface with the human body and perform measurements with high sensitivity over extended periods of time. Hydrogel‐based soft bioelectronics are useful for their ability to conform to different shapes and adhere to soft tissues, however, these materials lack inherent conductivity and their swelling limits durability. Nanoengineering can impart conductivity to these structures and can also be used to reduce swelling, improving functionality as a soft bioelectronic. Carbon nanotubes functionalized with carboxyl and hydroxyl groups were added to a poly (acrylic acid) hydrogel to improve its conductivity and reduce swelling to form a soft strain sensor. Tannic acid was also distributed throughout the hydrogel matrix and interacted with the carbon nanotubes to form nanoconfinement structures that imparted toughness to the overall system. Nanotubes homogeneously dispersed throughout the hydrogel matrix led to a concentration‐dependent improvement in conductivity. Additionally, hydrophobic moieties present on the nanotubes contributed to a two‐fold reduction in equilibrium swelling degree.^[^ ^276^ ^]^

## 7 Toxicity and Biocompatibility of Nanoengineered Biomaterials

### 7.1 Unique Safety Concerns with Nanoengineered Biomaterials

**Figure 7:**
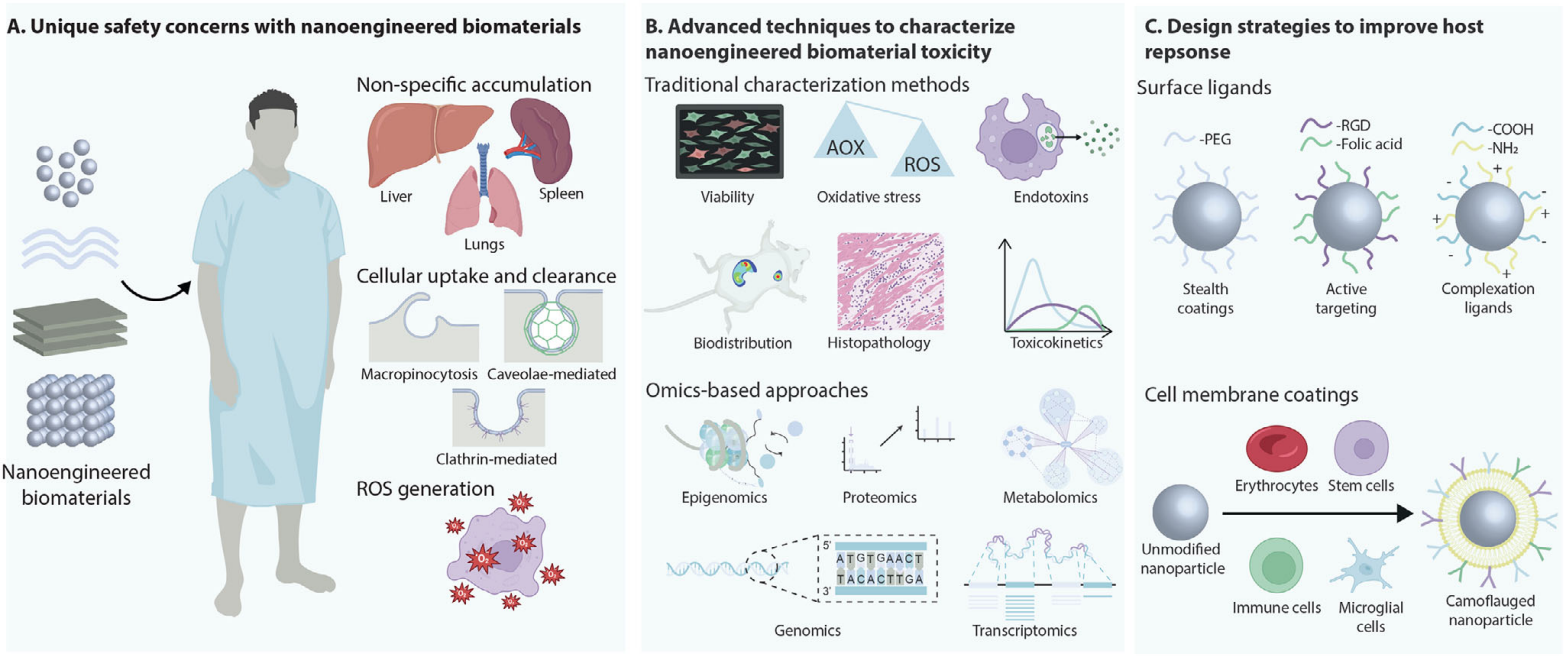
Emerging directions toward designing the next generation of nanoengineered biomaterials. A) Nanoengineered biomaterials pose unique safety concerns, including non‐specific accumulation, unwanted cellular uptake, and rapid clearance, and the generation of reactive oxygen species. B) Traditional toxicity assays primarily assess short‐term, localized effects such as cell viability, oxidative stress, and toxicokinetics, but often fail to reveal underlying biological mechanisms. In contrast, omics‐based approaches enable a more comprehensive understanding of the interactions between nanoengineered biomaterials and biological systems. C) Strategies to improve the host response to nanoengineered biomaterials include engineering surface ligands for stealth, active targeting, or complexation. Additionally, cloaking nanoengineered biomaterials with cell membranes enables immune evasion and can introduce self‐like receptors that promote specific cellular uptake and facilitate transport across biological barriers. The figure was created in Adobe Illustrator using the icons from BioRender.com.

The safety profile of biomaterials is fundamentally influenced by the host response, which is initiated by protein‐material interactions immediately upon exposure to the extracellular environment. At the nanoscale, small changes in particle shape, size, surface charge, or hydrophobicity can significantly alter these interactions, affecting immune recognition, biodistribution, and clearance mechanisms. Nanoengineered biomaterials possess a high surface area‐to‐volume ratio and small size, which enable them to penetrate biological barriers, accumulate in tissues, and engage in biological processes not typically observed with bulk or microscale materials. These unique behaviors present distinct safety concerns (**Figure** **7**A). For instance, polystyrene nanoparticles demonstrate enhanced cellular uptake, leading to increased mitochondrial damage and apoptosis, whereas polystyrene microparticles tend to promote bacterial aggregation and stimulate adaptive immune responses.^[^ ^277^ ^]^ Nickel oxide nanoparticles induce higher oxidative stress and inflammatory markers in rat liver tissue compared to their microscale counterparts.^[^ ^278^ ^]^ Similarly, as titanium dioxide nanoparticles decrease in size, they evade phagocytosis by alveolar macrophages, enabling translocation to distal organs and inducing oxidative stress responses.^[^ ^279^ ^]^ These examples underscore the necessity of evaluating nanoengineered biomaterials as distinct entities rather than extrapolating safety data from bulk materials. Their size‐ and surface‐dependent properties elicit unique biological effects that require dedicated investigation to ensure safe clinical translation.

### 7.2 Advanced Techniques to Characterize Nanoengineered Biomaterial Toxicity

Recent research has increasingly focused on advanced characterization methods to evaluate the biocompatibility and potential toxicity of nanoengineered biomaterials in both in vitro and in vivo systems. While traditional assays, such as those assessing cellular viability, oxidative stress, endotoxin levels, and acute inflammatory responses, remain valuable for identifying short‐term and localized effects,^[^ ^280^, ^281^ ^]^ they offer limited insight into the molecular mechanisms underlying long‐term, system‐wide biological interactions. To address these limitations, omics‐based approaches have emerged as powerful tools for elucidating the mechanisms of nanomaterial toxicity (Figure 7B): Proteomics characterizes the protein corona that forms on nanomaterial surfaces upon exposure to biological fluids. For example, liquid chromatography–mass spectrometry has revealed that the surface chemistry of graphene oxide‐based nanomaterials influences the enrichment of specific proteins (e.g., D‐binding protein, inter‐alpha trypsin inhibitor heavy chain H2), which correlates with enhanced cellular uptake and pro‐inflammatory responses.^[^ ^282^ ^]^ Genomics and transcriptomics provide insight into alterations in gene expression induced by material exposure. In a study of long‐term pulmonary exposure to multi‐walled carbon nanotubes, transcriptomic and epigenomic profiling identified nearly 1000 genes with changes in DNA methylation and mRNA expression. Affected pathways included immune regulation, extracellular matrix remodeling, and smooth muscle contraction.^[^ ^283^ ^]^ Epigenomics investigates modifications to DNA and chromatin architecture beyond changes in sequence. These alterations, such as methylation or histone modification, can influence gene expression and cellular phenotype, revealing long‐term effects of nanomaterial exposure. Metabolomics and lipidomics quantify dynamic changes in small molecules and lipids that reflect cellular metabolic responses. In a mouse model of systemic inflammation, mass spectrometry‐based profiling uncovered shifts in lipid classes—including elevated phosphatidylcholines and sphingomyelins, and decreased triacylglycerols and lysophosphatidylcholines—which corresponded with distinct macrophage activation profiles.^[^ ^284^ ^]^ Together, these high‐resolution techniques provide a systems‐level perspective on nano‐bio interactions, enabling mechanistic insights that complement traditional toxicological endpoints. By uncovering molecular signatures of toxicity, omics approaches support regulatory decision‐making and inform the safe design and clinical translation of nanoengineered biomaterials.

### 7.3 Design Strategies to Improve Host Response

In parallel with advances in characterization, the design of nanoengineered biomaterials has increasingly focused on strategies that enhance biocompatibility and minimize adverse host responses. These include surface modifications that prolong circulation, reduce immune recognition, and facilitate targeted cellular interactions. Actively engineering the nano‐bio interface enables more precise control over biological responses and reduces systemic toxicity. A central challenge in nano‐biomaterial design is evading clearance by the mononuclear phagocytic system. Surface coatings, such as PEGylation, offer a widely used solution by imparting steric stabilization and immune “stealth” properties (Figure 7C). For example, coating gold nanorods with polyethylene glycol (PEG) significantly extended their circulation time by enhancing colloidal stability and reducing opsonization and phagocytic clearance in vivo.^[^ ^285^ ^]^ Beyond passive stealth, active targeting strategies involve conjugation of ligands—such as folic acid or arginine‐glycine‐aspartic acid (RGD) peptides—to nanoparticle surfaces to promote receptor‐mediated uptake (Figure 7C). Folic acid targets overexpressed folate receptors on tumor cells; for example, silica nanoparticles functionalized with PEG and folic acid demonstrated enhanced uptake by MDA‐MB‐231 and HeLa cells, enabling efficient siRNA delivery and gene silencing.^[^ ^286^ ^]^ Similarly, RGD peptides bind to integrins (e.g., αvβ3) prevalent in tumor vasculature. In one study, gold nanostars modified with polydopamine and RGD peptides achieved selective internalization by HepG2 liver cancer cells, inducing mitochondrial and autophagy‐associated cell death with minimal off‐target effects.^[^ ^287^ ^]^ Cell membrane coatings offer a biomimetic strategy to evade immune surveillance by cloaking nanoparticles in membranes derived from red blood cells (RBCs), stem cells, or immune cells (Figure 7C). These coatings modulate the protein corona, presenting “self” signals that reduce clearance and enhance circulation. RBC membranes, for instance, have been used to coat doped bioactive glass nanoparticles, improving tumor accumulation and enhancing near‐infrared (NIR)‐induced apoptosis and tumor regression.^[^ ^288^, ^289^ ^]^ Stem cell membrane coatings offer additional targeting advantages through tissue‐specific homing. For example, membranes from pre‐conditioned adipose‐derived mesenchymal stem cells upregulated chemokine receptors (CCR1, CCR2, CXCR4), promoting chemotactic accumulation of nanoparticles in tumor sites.^[^ ^290^, ^291^ ^]^ In another example, coating ZIF‐8 nanoparticles with stem cell membranes facilitated homotypic uptake by mesenchymal stem cells and promoted osteogenic differentiation, enhancing regenerative efficacy.^[^ ^292^ ^]^ Immune cell membranes, particularly T‐cell‐derived coatings, add functional targeting capabilities. PLGA nanoparticles coated with T‐cell membranes retained surface proteins like FasL, PD1, and CD47, allowing them to evade macrophage uptake while inducing FasL‐mediated apoptosis in cancer cells via upregulation of BAX expression.^[^ ^293^ ^]^ A notable application of membrane coating is in crossing the blood‐brain barrier (BBB). For instance, microRNA nanosponges coated with BV2 microglial cell membranes demonstrated enhanced targeting of glioblastoma via CX3CL1 and CSF1 signaling, leading to selective uptake, suppression of oncogenic pathways, and extended survival in glioblastoma‐bearing mice.^[^ ^294^ ^]^ These membrane‐based and ligand‐mediated strategies demonstrate the versatility of surface engineering approaches in enhancing circulation, targeting specificity, and therapeutic efficacy of nanoengineered biomaterials across a range of biomedical applications.

## 8 Future Outlook

Progress in analytical chemistry and polymer science has contributed toward the field of nanoengineered biomaterials. By providing better structural control over these nanomaterials, their properties can be fine‐tuned towards achieving their successful use in fields of drug delivery, regenerative medicine, and other biomedical applications. As the relationship between properties like surface energy, porosity, crystallinity, and atomic defects with material functionalities are further explored mechanistically, the application nanoengineered materials will expand to achieve highly controlled, precise biomedical devices with clinical translation. In this section, we highlight the emerging research directions that are working toward this end.

### 8.1 Exploring Organelle‐Specific Targeting for Enhanced Therapeutic Effects

**Figure 8:**
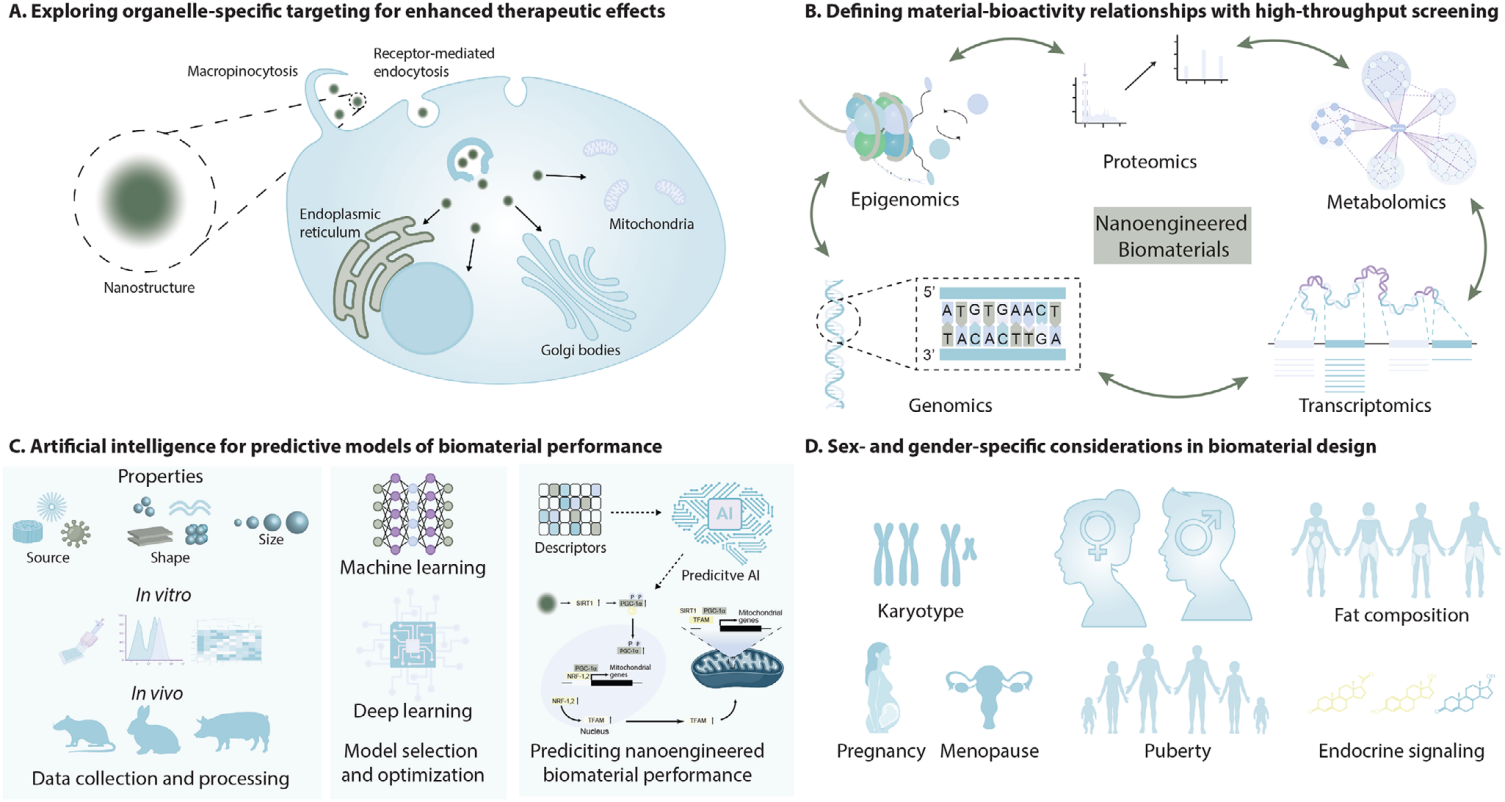
Emerging directions toward designing the next generation of nanoengineered biomaterials. A) Organelle‐specific targeting for structures such as mitochondria, endoplasmic reticula, nuclei, or Golgi bodies will allow for an improved understanding of nanoengineered biomaterial performance post‐internalization. B) High‐throughput molecular biology assays and the integration of multiple techniques will assist in defining relationships between material properties and their biological activity. C) Training different artificial intelligence models on the performance of nanoengineered biomaterials in vitro and in vivo will lead to the generation of predictive models that can suggest the most probable biomedical outcomes based on material properties. D) Sex and gender should be considered as primary criteria in the design and implementation of nanoengineered biomaterials to ensure their bioactivity can be tailored for specific applications. The figure was created in Adobe Illustrator using the icons from BioRender.com.

The uncertainty surrounding payload fete following intracellular delivery has highlighted the need to direct attention toward sub‐organelle targeting. This becomes increasingly important since many of the deleterious disorders originate through organelle dysfunction.^[^ ^295^ ^]^ Recently there has been growing interest in the field and much of the current work has been toward targeting mitochondria and nuclei.^[^ ^296^ ^]^ To this end, nuclear localization signal‐conjugated polymersome nanocarriers were reported to demonstrate successful nucleocytoplasmic transport to bypass nuclear pore complexes.^[^ ^297^ ^]^ There has also been active research in the field of organelle targeting for cancer treatment. This has focused on delivering drugs to multiple different organelles (**Figure** **8**A).^[^ ^298^ ^]^ As the field progresses there is a need to look for other potential targets and obtain an in‐depth understanding of the mechanisms of intracellular trafficking in organelle‐targeted nanoplatforms.

### 8.2 Defining Relationships Between Material Properties and Biological Activity with High‐Throughput Molecular Screening

Traditional low‐throughput molecular biology assays provide limited information on the effects of nanomaterials on cellular processes. The recent emergence of ‐omics based techniques including epigenomics, genomics, metabolomics, proteomics, and transcriptomics has allowed a more comprehensive approach to nanoengineered biomaterial screening (Figure 8B). While each of these techniques can provide sufficient insight into a single component of the dynamic biological response to nanomaterials, the integration of multiple ‐omics based strategies would further capture the dynamicity and heterogeneity of these interactions.^[^ ^299^ ^]^ Additionally, the breadth of data that has been gathered using traditional, ‐omics based, and multi‐omics based molecular assays will eventually be used to form datasets for artificial intelligence‐based approaches to predict the responses of entire organ systems to applied nanoengineered materials. As the field of biomedicine is constantly progressing, there has been an increased focus on how the response to biomaterials changes based on sex or gender. AI‐based predictions can use the knowledge gathered since the earliest biomaterial research was published to shine a light on this aspect, improving the design of future materials for patient‐specific treatments.

### 8.3 Artificial Intelligence for Predictive Models of Nanoengineered Biomaterial Performance

Nanoengineered biomaterials can be derived from a variety of sources and are extensively tuned for different applications. While this unique versatility is useful, it also results in a lack of consistency in understanding relationships between material properties and performance. Recently, artificial intelligence has been increasingly integrated with nanoengineering to optimize the design, fabrication, biomedical applications, and environmental sustainability of nanoengineered materials.^[^ ^300^ ^]^ Nanotechnologies have allowed for advanced diagnostic and therapeutic techniques that show promise in individualized approaches.^[^ ^301^ ^]^ The integration of artificial intelligence enhances this potential through its ability to analyze and correlate extensive variables to determine optimal material designs for specific patient conditions (Figure 8C). This intersection has led to the development of promising approaches such as generative adversarial networks for nanomaterial design^[^ ^302^ ^]^ or convolutional neural networks to predict nanomaterial bioactivity.^[^ ^303^ ^]^ To further develop this convergence between artificial intelligence and nanoengineering, large amounts of reliable data are required for training various models.^[^ ^304^ ^]^ However, this is limited by the conventional approaches to assess nanomaterial performance, relating macro‐ and microscale data back to nanoscale phenomena with assumptions or general, indirect models that result in variability. Additionally, the lack of regulatory bodies surrounding this emerging field will hinder significant advancements initially. Collaborations between computer engineers, data scientists, materials scientists, chemists, and bioengineers will lead to interdisciplinary approaches to rapidly advance the merging of nanoengineering with artificial intelligence.

### 8.4 Sex‐ and Gender‐Specific Considerations in Nanoengineered Biomaterial Design

One factor that warrants careful consideration when investigating the clinical performance of nanotherapeutics is the effect of sex and gender (Figure 8D). Differences in the protein corona composition, nanoparticle uptake from mesenchymal stem cells, toxicity profile among others have been previously well studied. There has also been evidence of differential response to vaccines.^[^ ^305^ ^]^ This necessitates the strict inclusion of sex and gender as primary criteria in clinical trials, thus far lacking despite national mandates.^[^ ^306^ ^]^ Consideration should also be given to sex‐ and gender‐related differences in biodistribution, metabolism, and excretion when designing and applying nanomaterials.

## 9 Conclusion

Nanoengineering enhances the bioactivity of conventional biomaterials through strengthening biophysical and biochemical cues, tuning mechanical properties, and imparting stimuli‐responsiveness. These advanced biomaterials show improved performance in biomedical applications such as tissue regeneration, drug delivery, additive manufacturing, and biosensing. Engineered surface nanofeatures provide a biomimetic structure for cells to attach and proliferate, while the inherent bioactivity of nanomaterials initiates signaling pathways for a heightened therapeutic effect. Nanoengineered biomaterials also improve the efficacy of therapeutic agents by prolonging their circulation and performing localized delivery to the target tissues. Their stimuli‐responsive properties impart dynamicity to biomaterials, allowing for adaptive systems that synchronize their bioactivity to different physiological processes.

Designing the next generation of nanoengineered biomaterials will require a more comprehensive understanding of the relationship between nanomaterial surface properties and their interactions with cells, tissues, extracellular matrix components, and subcellular organelles. Advanced molecular biology assays including multi‐omics approaches should be used to further explore specific biological mechanisms and how different properties of nanoengineered materials influence them. As this would likely result in significantly varying results, powerful analysis tools based on artificial intelligence should be integrated with nanoengineering to define connections and predict nanoengineered biomaterial performance in specific applications. This would allow for the investigation of more nuanced variables, such as the effect of sex and gender on the performance of nanoengineered biomaterials.

## Conflict of Interest

The authors declare no conflict of interest.

## Author Contributions

R.D. and I.D. contributed equally to this work. R.D., I.D., N.A.P., and A.K.G. contributed to the article's conception, research, discussion, and final edits and revisions.

## References

[1] H. Ai , J. Anderson , K. Anseth , I. Antoniac , M. Barbosa , B. Basu , S. Best , R. Bettini , D. Bezuidenhout , R. Bizios , J. Brash , Y. Cao , J. Chang , G. Chen , E. Cosgriff‐Hernandez , A. Coury , J. Ding , X. Fu , A. García , B. Harley , J. Ji , K. Kataoka , J. Kohn , C. Laurencin , K. Leong , J.‐C. Lin , C. Liu , H. Lu , P. Ma , K. McLean , et al., in Definitions of Biomaterials for the Twenty‐First Century, (Eds: D. Williams , X. Zhang ), Elsevier, Cham 2019, II.

[2] R. P. Feynman , Resonance 1999, 16, 890.

[3] Y. Ying , Z. Huang , Y. Tu , Q. Wu , Z. Li , Y. Zhang , H. Yu , A. Zeng , H. Huang , J. Ye , W. Ying , M. Chen , Z. Feng , Z. Xiang , Q. Ye , S. Zhu , Z. Wang , Bioact. Mater. 2023, 22, 274. 36263097 10.1016/j.bioactmat.2022.09.019 PMC9556860

[4] H. Samadian , H. Khastar , A. Ehterami , M. Salehi , Sci. Rep. 2021, 11, 13877. 34230542 10.1038/s41598-021-93367-6 PMC8260712

[5] S. H. Zhu , S. Wang , Y. F. Huang , Q. Y. Tang , T. Q. Fu , R. Y. Su , C. Y. Fan , S. Xia , P. S. Lee , Y. H. Lin , Nat. Commun. 2024, 15, 118. 38168050 10.1038/s41467-023-44481-8 PMC10761753

[6] J. Li , Z. W. Li , Y. Xie , T. Cai , D. Shin , C. J. Chen , C. Mirkin , Adv. Mater. 2024, 36. 10.1002/adma.202408153 39128135

[7] B. M. Foley , S. C. Hernández , J. C. Duda , J. T. Robinson , S. G. Walton , P. E. Hopkins , Nano Lett. 2015, 15, 4876. 26125524 10.1021/acs.nanolett.5b00381

[8] K. A. Singh , J. Soukar , M. Zulkifli , A. Kersey , G. Lokhande , S. Ghosh , A. Murali , N. M. Garza , H. Kaur , J. N. Keeney , R. Banavath , H. Ceylan Koydemir , R. Sitcheran , I. Singh , V. M. Gohil , A. K. Gaharwar , Nat. Commun. 2024, 15, 8136. 39289340 10.1038/s41467-024-52276-8 PMC11408498

[9] J. M. Maita , S. Rommel , J. R. Davis , H. Ryou , J. A. Wollmershauser , E. P. Gorzkowski , B. N. Feigelson , M. Aindow , S. W. Lee , Acta Mater. 2023, 251, 118881.

[10] M. K. Ahmed , M. A. Zayed , S. I. El‐dek , M. A. Hady , D. H. El Sherbiny , V. Uskokovic , Bioact. Mater. 2021, 6, 2070. 33511308 10.1016/j.bioactmat.2020.12.026 PMC7809176

[11] a) P. Verma , J. Ubaid , K. M. Varadarajan , B. L. Wardle , S. Kumar , ACS Appl. Mater. Interfaces 2022, 14, 8361; 35119271 10.1021/acsami.1c20491

[12] a) E. Koukouviti , A. Economou , C. Kokkinos , Adv. Funct. Mater. 2024, 34;

[13] K. A. Deo , M. K. Jaiswal , S. Abasi , G. Lokhande , S. Bhunia , T. U. Nguyen , M. Namkoong , K. Darvesh , A. Guiseppi‐Elie , L. M. Tian , A. K. Gaharwar , ACS Nano 2022, 16, 8798. 35675588 10.1021/acsnano.1c09386 PMC13050497

[14] a) Y. X. Wang , C. H. Sun , Z. Y. Liu , S. M. Zhang , K. Gao , F. Yi , W. J. Zhou , H. Liu , Adv. Funct. Mater. 2024;

[15] M. Lele , S. Kapur , S. Hargett , N. M. Sureshbabu , A. K. Gaharwar , Sci. Adv. 2024, 10, abq0997. 10.1126/sciadv.abq0997 PMC466960 39018412

[16] T. Y. Wang , Y. X. Li , Y. N. Liu , Z. Q. Xu , M. Y. Wen , L. B. Zhang , Y. M. Xue , L. Shang , J. Colloid Interface Sci. 2023, 633, 851. 36495807 10.1016/j.jcis.2022.11.139

[17] Z. B. Zhang , J. J. Ren , W. B. Dai , H. Zhang , X. Q. Wang , B. He , Q. Zhang , Adv. Mater. 2023, 35. 10.1002/adma.202206636 36477943

[18] W. Y. Zhou , C. L. O'Neill , T. B. Ding , O. M. Zhang , J. S. Rudra , M. D. Lew , ACS Nano 2024, 18, 8798. 38478911 10.1021/acsnano.3c11771 PMC11025465

[19] Z. Q. Wan , X. Q. Bai , X. Wang , X. D. Guo , X. Wang , M. Zhai , Y. Fu , Y. S. Liu , P. Zhang , X. Zhang , R. L. Yang , Y. Liu , L. W. Lv , Y. S. Zhou , Adv. Sci. 2024, 11. 10.1002/advs.202308986 PMC11187922 38588510

[20] A. Keklikian , N. R. de Barros , A. Rashad , Y. Q. Chen , J. R. Tan , R. Y. Sheng , D. W. Sun , H. A. Liu , F. G. Thankam , Gels 2024, 10, 46. 38247769 10.3390/gels10010046 PMC10815274

[21] a) Y. C. Liu , Y. K. Lin , Y. T. Lin , C. W. Lin , G. Y. Lan , Y. C. Su , F. R. Hu , K. H. Chang , V. C. Chen , Y. C. Yeh , T. C. Chen , J. S. Yu , Adv. Sci. 2024; 10.1002/advs.202308635 PMC10953571 38233151

[22] a) S. Ghalei , M. Douglass , H. Handa , ACS Biomater. Sci. Eng. 2022; 10.1021/acsbiomaterials.1c01121 34890206

[23] L. Zou , L. Hu , P. P. Pan , S. Tarafder , M. Z. Du , Y. S. Geng , G. Xu , L. Chen , J. D. Chen , C. H. Lee , Compos. Pt B Eng. 2022, 232, 109625.

[24] S. Wu , Y. Li , C. Zhang , L. Tao , M. Kuss , J. Y. Lim , J. Butcher , B. Duan , Adv. Healthcare Mater. 2022, 11, 2200053. 10.1002/adhm.202200053 PMC10976923 35289986

[25] Y. Yang , Y. Ding , B. Fan , Y. Wang , Z. Mao , W. Wang , J. Wu , J. Controlled Release 2020, 321, 463. 10.1016/j.jconrel.2020.02.030 32087302

[26] C. Xu , W. Liu , Y. Hu , W. Li , W. Di , Theranostics 2020, 10, 3325. 32194871 10.7150/thno.41228 PMC7053183

[27] L. M. Cross , J. K. Carrow , X. Ding , K. A. Singh , A. K. Gaharwar , ACS Appl. Mater. Interfaces 2019, 11, 6741. 30676016 10.1021/acsami.8b17733 PMC6472961

[28] X. Zhang , J. Fan , C.‐S. Lee , S. Kim , C. Chen , M. Lee , ACS Appl. Mater. Interfaces 2020, 12, 16088. 32175721 10.1021/acsami.0c01241 PMC7161535

[29] J. H. Park , J. A. Jackman , A. R. Ferhan , J. N. Belling , N. Mokrzecka , P. S. Weiss , N.‐J. Cho , ACS Nano 2020, 14, 11950. 32845615 10.1021/acsnano.0c05097

[30] X. Zhang , F. Chen , M. Z. Turker , K. Ma , P. Zanzonico , F. Gallazzi , M. A. Shah , A. R. Prater , U. Wiesner , M. S. Bradbury , M. R. McDevitt , T. P. Quinn , Biomaterials 2020, 241, 119858. 32120314 10.1016/j.biomaterials.2020.119858 PMC7171978

[31] Y.‐H. Kim , X. Yang , L. Shi , S. A. Lanham , J. Hilborn , R. O. C. Oreffo , D. Ossipov , J. I. Dawson , Nat. Commun. 2020, 11, 10.1038/s41467-020-15152-9. PMC7069965 32170076

[32] W. Du , S. Du , X. Dong , H. Bai , J. Jiang , S. Hao , F. Yang , Q. Xiao , B. Zhang , J. Ge , L. Gao , L. Li , S. Q. Yao , W. Huang , Biomaterials 2023, 294, 122000. 36640541 10.1016/j.biomaterials.2023.122000

[33] Y. Zhang , J. Li , P. Habibovic , Bioactive Materials 2022, 15, 372. 35386339 10.1016/j.bioactmat.2022.02.028 PMC8958423

[34] A. Zamuner , P. Brun , R. Ciccimarra , F. Ravanetti , L. Veschini , H. Elsayed , S. Sivolella , G. Iucci , A. Porzionato , L. D. Silvio , A. Cacchioli , E. Bernardo , M. Dettin , Biomed. Mater. 2021, 16, 055007. 10.1088/1748-605X/ac1555 34271554

[35] A. M. Brokesh , L. M. Cross , A. L. Kersey , A. Murali , C. Richter , C. A. Gregory , I. Singh , A. K. Gaharwar , Sci. Adv. 2022, 8, 10.1126/sciadv.abl9404. PMC9045714 35476448

[36] S. Sánchez‐Salcedo , C. Heras , D. Lozano , M. Vallet‐Regí , A. J. Salinas , Acta Biomater. 2023, 166, 655. 37142110 10.1016/j.actbio.2023.04.046

[37] X. Han , C. Saengow , L. Ju , W. Ren , R. H. Ewoldt , J. Irudayaraj , Nat. Commun. 2024, 15, 10.1038/s41467-024-47696-5. PMC11039765 38653959

[38] Y. Chen , Y. Zhang , B. Wang , Q. Fan , Q. Yang , J. Xu , H. Dai , F. Xu , C. Wang , ACS Nano 2022, 17, 760. 36520665 10.1021/acsnano.2c10797

[39] J. Lin , Z. Wang , J. Huang , S. Tang , Q. Saiding , Q. Zhu , W. Cui , Small 2021, 17, 2007235. 10.1002/smll.202007235 33590681

[40] A. Fluksman , A. Lafuente , R. Braunstein , E. Steinberg , N. Friedman , Z. Yekhin , A. G. Roca , J. Nogues , R. Hazan , B. Sepulveda , O. Benny , ACS Appl. Mater. Interfaces 2023, 15, 50330. 37861446 10.1021/acsami.3c07188 PMC10623511

[41] S. Li , B. Gu , X. Li , S. Tang , L. Zheng , E. Ruiz‐Hitzky , Z. Sun , C. Xu , X. Wang , Adv. Healthcare Mater. 2022, 11, 2102367. 10.1002/adhm.202102367 35285165

[42] J. Kim , J. Roh , M. Park , C. Lee , Adv. Mater. 2023, 36, 2212220. 10.1002/adma.202212220 36853911

[43] a) Y. Bai , C. L. Liu , Y. Y. Shan , T. T. Chen , Y. Zhao , C. Yu , H. Pang , Adv. Energy Mater. 2022, 12, 2100346;

[44] L. K. Putri , B.‐J. Ng , W.‐J. Ong , H. W. Lee , W. S. Chang , A. R. Mohamed , S.‐P. Chai , Appl. Catal., B 2020, 265, 118592.

[45] W. Fu , H. Cao , A. K. Cheetham , Adv. Opt. Mater. 2023, 11, 2300434.

[46] H. Zhang , X. Dong , J. Wang , R. Guan , D. Cao , Q. Chen , ACS Appl. Mater. Interfaces 2019, 11, 32489. 31393690 10.1021/acsami.9b09545

[47] F. Krieg , P. C. Sercel , M. Burian , H. Andrusiv , M. I. Bodnarchuk , T. Stöferle , R. F. Mahrt , D. Naumenko , H. Amenitsch , G. Rainò , M. V. Kovalenko , ACS Cent. Sci. 2020, 7, 135. 33532576 10.1021/acscentsci.0c01153 PMC7845019

[48] C. Yang , H. Aslan , P. Zhang , S. Zhu , Y. Xiao , L. Chen , N. Khan , T. Boesen , Y. Wang , Y. Liu , L. Wang , Y. Sun , Y. Feng , F. Besenbacher , F. Zhao , M. Yu , Nat. Commun. 2020, 11, 10.1038/s41467-020-14866-0. PMC7070098 32170166

[49] C. Siefe , R. D. Mehlenbacher , C. S. Peng , Y. Zhang , S. Fischer , A. Lay , C. A. McLellan , A. P. Alivisatos , S. Chu , J. A. Dionne , J. Am. Chem. Soc. 2019, 141, 16997. 31592655 10.1021/jacs.9b09571 PMC8259630

[50] J. W. Myerson , P. N. Patel , K. M. Rubey , M. E. Zamora , M. H. Zaleski , N. Habibi , L. R. Walsh , Y.‐W. Lee , D. C. Luther , L. T. Ferguson , O. A. Marcos‐Contreras , P. M. Glassman , L. L. Mazaleuskaya , I. Johnston , E. D. Hood , T. Shuvaeva , J. Wu , H.‐Y. Zhang , J. V. Gregory , J. S. Brenner , Nat. Nanotechnol. 2021, 17, 86. 34795440 10.1038/s41565-021-00997-y PMC8776575

[51] M. Satpathy , L. Wang , R. J. Zielinski , W. Qian , Y. A. Wang , A. M. Mohs , B. A. Kairdolf , X. Ji , J. Capala , M. Lipowska , S. Nie , H. Mao , L. Yang , Theranostics 2019, 9, 778. 30809308 10.7150/thno.29964 PMC6376473

[52] S. K. Ghosh , D. Mandal , Nano Energy 2018, 53, 245.

[53] D. Wang , H. Peng , B. Yu , K. Zhou , H. Pan , L. Zhang , M. Li , M. Liu , A. Tian , S. Fu , Chem. Eng. J. 2020, 389, 124449.

[54] H. Kim , J. A. Lee , C. P. Ambulo , H. B. Lee , S. H. Kim , V. V. Naik , C. S. Haines , A. E. Aliev , R. Ovalle‐Robles , R. H. Baughman , T. H. Ware , Adv. Funct. Mater. 2019, 29, 1905063.

[55] T. E. Glier , M. Betker , M. Witte , T. Matsuyama , L. Westphal , B. Grimm‐Lebsanft , F. Biebl , L. O. Akinsinde , F. Fischer , M. Rübhausen , Nanoscale 2020, 12, 23831. 33237101 10.1039/d0nr05734g

[56] L. Wang , T. Zhu , Y. Kang , J. Zhang , J. Du , H. Gao , S. Chen , J. Jiang , J. Zhao , Bioactive Materials 2022, 16, 149. 35386329 10.1016/j.bioactmat.2022.01.031 PMC8958472

[57] F. Yang , J. Zhao , W. J. Koshut , J. Watt , J. C. Riboh , K. Gall , B. J. Wiley , Adv. Funct. Mater. 2020, 30, 2003451.

[58] L. Li , C. Wu , Y. Ling , C. Hou , Q. Zhang , Y. Li , H. Shi , H. Wang , C. Li , S. Yin , Materials Today Nano 2022, 20, 100256.

[59] X. Hu , X. Wang , Y. Xu , L. Li , J. Liu , Y. He , Y. Zou , L. Yu , X. Qiu , J. Guo , Adv. Healthcare Mater. 2020, 9, 1901570. 10.1002/adhm.201901570 32338461

[60] T. Hu , B. Xue , F. Meng , L. Ma , Y. Du , S. Yu , R. Ye , H. Li , Q. Zhang , L. Gu , Z. Zhou , R. Liang , C. Tan , Adv. Healthcare Mater. 2023, 12, 2202911. 10.1002/adhm.202202911 36603589

[61] J. T. DuBose , A. Christy , J. Chakkamalayath , P. V. Kamat , ACS Mater. Lett. 2021, 4, 93.

[62] H. Jiang , Q. Xia , J. Zheng , J. Bu , R. Li , Z. Cai , K. Ling , Biosens. Bioelectron. 2022, 216, 114622. 35973273 10.1016/j.bios.2022.114622

[63] T. Xia , Z. Tong , Y. Xie , M. C. Arno , S. Lei , L. Xiao , J. Y. Rho , C. T. J. Ferguson , I. Manners , A. P. Dove , R. K. O'Reilly , J. Am. Chem. Soc. 2023, 145, 25274. 37938914 10.1021/jacs.3c08770 PMC10682995

[64] G. Zhang , Y. Ji , X. Li , X. Wang , M. Song , H. Gou , S. Gao , X. Jia , Adv. Healthcare Mater. 2020, 9, 2000221. 10.1002/adhm.202000221 32548971

[65] A. Murali , G. Lokhande , K. A. Deo , A. Brokesh , A. K. Gaharwar , Mater. Today 2021, 50, 276. 10.1016/j.mattod.2021.04.020 PMC8713997 34970073

[66] Y. Shi , C. Liu , L. Liu , L. Fu , B. Yu , Y. Lv , F. Yang , P. Song , Chem. Eng. J. 2019, 378, 122267.

[67] P. Lin , Y. Xue , X. Mu , Y. Shao , Q. Lu , X. Jin , E. Yinwang , Z. Zhang , H. Zhou , W. Teng , H. Sun , W. Chen , W. Shi , C. Shi , X. Zhou , X. Jiang , X. Yu , Z. Ye , Small 2022, 18, 2200179. 10.1002/smll.202200179 35396783

[68] X. Yuan , Y. Zhu , S. Li , Y. Wu , Z. Wang , R. Gao , S. Luo , J. Shen , J. Wu , L. Ge , J. Nanobiotechnol. 2022, 20, 10.1186/s12951-022-01374-0. PMC8944145 35331256

[69] Z. Lv , T. Hu , Y. Bian , G. Wang , Z. Wu , H. Li , X. Liu , S. Yang , C. Tan , R. Liang , X. Weng , Adv. Mater. 2022, 35, 2206545. 10.1002/adma.202206545 36426823

[70] Z. Li , Q. Wang , X. Yang , S. Song , J. Wang , S.‐F. Wang , Chem. Eng. J. 2022, 430, 133049.

[71] C. Du , M. Cao , G. Li , Y. Hu , Y. Zhang , L. Liang , Z. Liu , G. Chen , Adv. Funct. Mater. 2022, 32, 2206083.

[72] K. Jayakumar , M. B. Camarada , R. Rajesh , R. Venkatesan , H. Ju , V. Dharuman , Y. Wen , Biosens. Bioelectron. 2018, 120, 55. 30145435 10.1016/j.bios.2018.08.032

[73] Z. Wu , H. Wu , W. Cai , Z. Wen , B. Jia , L. Wang , W. Jin , T. Ma , Angew. Chem., Int. Ed. 2021, 60, 12554. 10.1002/anie.202102832 33720479

[74] W. Wu , Y. Zhou , J. Pan , Y. Wu , G. Goksen , P. Shao , Carbohydr. Polym. 2023, 322, 121320. 37839838 10.1016/j.carbpol.2023.121320

[75] A. Ruiz‐Clavijo , O. Caballero‐Calero , C. V. Manzano , X. Maeder , A. Beardo , X. Cartoixà , F. X. Álvarez , M. Martín‐González , ACS Appl. Energy Mater. 2021, 4, 13556. 35647490 10.1021/acsaem.1c02129 PMC9127787

[76] Y. Li , J. Jia , H. Yu , S. Wang , Z.‐Y. Jin , Y.‐H. Zhang , H.‐Z. Ma , K. Zhang , K. Ke , B. Yin , M.‐B. Yang , ACS Appl. Mater. Interfaces 2022, 14, 15678. 35321545 10.1021/acsami.2c02090

[77] J. Chen , Z. Huang , H. Zhang , Z. Zhang , D. Wang , D. Xia , C. Yang , M. Dong , Chem. Eng. J. 2022, 443, 136234.

[78] S. S. Lee , G. E. Choi , H. J. Lee , Y. Kim , J.‐H. Choy , B. Jeong , ACS Appl. Mater. Interfaces 2017, 9, 42668. 29165981 10.1021/acsami.7b17173

[79] H. Yu , X. Chen , J. Cai , D. Ye , Y. Wu , L. Fan , P. Liu , Chem. Eng. J. 2019, 369, 253.

[80] H. A. Vignolo‐González , A. Gouder , S. Laha , V. Duppel , S. Carretero‐Palacios , A. Jimenez‐Solano , T. Oshima , P. Schützendübe , B. V. Lotsch , Adv. Energy Mater. 2023, 13. 10.1016/j.matt.2020.07.021 PMC7418450 32803152

[81] A. Galindo , J. L. Reyes‐Rodríguez , C. Botez , M. Moreno , A. Ponce , Mater. Adv. 2022, 3, 4548.

[82] J. Zhang , A. N. Keith , S. S. Sheiko , X. H. Wang , Z. G. Wang , ACS Appl. Mater. Interfaces 2021, 13, 3278. 33416300 10.1021/acsami.0c21494

[83] Y. Qin , M. Sayyad , A. R. P. Montblanch , M. S. G. Feuer , D. Dey , M. Blei , R. Sailus , D. M. Kara , Y. X. Shen , S. Z. Yang , A. S. Botana , M. Atature , S. Tongay , Adv. Mater. 2022, 34, 2106222. 10.1002/adma.202106222 34813678

[84] W. Liu , W. J. Wang , X. Y. Dong , Y. Sun , ACS Appl. Mater. Interfaces 2020, 12, 12618. 32105446 10.1021/acsami.0c02342

[85] J. V. Gregory , D. R. Vogus , A. Barajas , M. A. Cadena , S. Mitragotri , J. Lahann , Adv. Healthcare Mater. 2020, 9, 2000564. 10.1002/adhm.202000564 32959525

[86] a) B. Haney , J. G. Werner , D. A. Weitz , S. Ramakrishnan , ACS Appl. Mater. Interfaces 2020, 12, 33439; 32598144 10.1021/acsami.0c11408

[87] B. D. Frank , M. Antonietti , L. Zeininger , Macromolecules 2021, 54, 981. 33518808 10.1021/acs.macromol.0c02152 PMC7842141

[88] J. Gu , Y. Huang , Z. Yan , D. He , Y. Zhang , J. Xu , Y. Li , X. Xie , J. Xie , D. Shi , R. Abagyan , J. Zhang , Q. Tan , ACS Appl. Mater. Interfaces 2020, 12, 31112. 32544316 10.1021/acsami.0c06207

[89] Z. Ding , Y. Wang , F. Chen , X. Hu , W. Cheng , Q. Lu , D. L. Kaplan , Adv. Funct. Mater. 2024, 34, 2308888.

[90] F. A. Chapa‐Villarreal , M. Miller , J. J. Rodriguez‐Cruz , D. Perez‐Carlos , N. A. Peppas , Biomaterials 2023, 300, 122191. 37295223 10.1016/j.biomaterials.2023.122191

[91] C. Wang , Z. Wang , X. Zhang , Acc. Chem. Res. 2012, 45, 608. 22242811 10.1021/ar200226d

[92] S. Chagri , D. Y. W. Ng , T. Weil , Nat. Rev. Chem. 2022, 6, 320. 37117928 10.1038/s41570-022-00373-x PMC8972907

[93] L. D. Yue , C. Gao , J. Y. Li , H. B. Chen , S. M. Y. Lee , R. F. Luo , R. B. Wang , Adv. Mater. 2023, 35. 10.1002/adma.202211626 36905923

[94] X. Zhao , K. X. Zhang , Y. Y. Wang , W. X. Jiang , H. Cheng , Q. W. Wang , T. T. Xiang , Z. Z. Zhang , J. J. Liu , J. J. Shi , Adv. Funct. Mater. 2022, 32, 2108883.

[95] W. B. Zhang , Z. H. Zhai , S. F. Li , X. Lin , W. Bai , N. Ding , Y. Zhang , J. Q. Tong , J. Z. Sun , C. Y. Gao , Nanoscale 2021, 13, 138. 33350429 10.1039/d0nr06661c

[96] R. S. Li , J. Liu , H. Shi , P. P. Hu , Y. Wang , P. F. Gao , J. Wang , M. Jia , H. Li , Y. F. Li , Nano Lett. 2021, 21, 8455. 34569805 10.1021/acs.nanolett.1c03112

[97] L. Wang , P. P. Yang , X. X. Zhao , H. Wang , Nanoscale 2016, 8, 2488. 26757620 10.1039/c5nr07437a

[98] a) D. Fan , B. Miller Naranjo , S. Mansi , P. Mela , O. Lieleg , ACS Appl. Mater. Interfaces 2023, 15, 37986; 37491732 10.1021/acsami.3c05298

[99] a) J. Min , R. D. Braatz , P. T. Hammond , Biomaterials 2014, 35, 2507; 24388389 10.1016/j.biomaterials.2013.12.009 PMC3951715

[100] J. Di , J. Wang , S. Wang , M. Ma , H. Zhang , N. Liu , A. Zheng , X. Gao , B. Liu , J. Gao , Small 2023, 19, 2207892. 10.1002/smll.202207892 36732845

[101] N. Boehnke , S. Correa , L. Hao , W. Wang , J. P. Straehla , S. N. Bhatia , P. T. Hammond , Angew. Chem., Int. Ed. 2020, 59, 2776. 10.1002/anie.201911762 PMC7002217 31747099

[102] A. Ivanova , K. Ivanova , A. Tied , T. Heinze , T. Tzanov , Adv. Funct. Mater. 2020, 30, 2001284.

[103] B. V. Slaughter , S. S. Khurshid , O. Z. Fisher , A. Khademhosseini , N. A. Peppas , Adv. Mater. 2009, 21, 3307. 20882499 10.1002/adma.200802106 PMC4494665

[104] a) P. X. Li , L. W. Fu , Z. Y. Liao , Y. Peng , C. Ning , C. J. Gao , D. X. Zhang , X. Sui , Y. F. Lin , S. Y. Liu , C. X. Hao , Q. Y. Guo , Biomaterials 2021, 278, 121131; 34543785 10.1016/j.biomaterials.2021.121131

[105] J. R. Clegg , A. S. Irani , E. W. Ander , C. M. Ludolph , A. K. Venkataraman , J. X. Zhong , N. A. Peppas , Sci. Adv. 2019, 5, aax7946. 10.1126/sciadv.aax7946 PMC6764836 31598554

[106] a) G. Li , C. Wang , Y. Chen , F. Liu , H. Fan , B. Yao , J. Hao , Y. Yu , D. Wen , Small 2023, 19, 2206868; 10.1002/smll.202206868 36710247

[107] a) L. Chang , S. Chen , Y. Fei , D. J. Stacchiola , Y. H. Hu , Proc. Natl. Acad. Sci. USA 2023, 120, 2219950120; 10.1073/pnas.2219950120 PMC10041093 36913567

[108] T. Li , Y. Yuan , L. Gu , J. Li , Y. Shao , S. Yan , Y. Zhao , C. Carlos , Y. Dong , H. Qian , Sci. Adv. 2024, 10, adn8706. 10.1126/sciadv.adn8706 PMC11259165 39028816

[109] S. Ozcan , P. Kaner , D. Thomas , P. Cebe , A. Asatekin , ACS Appl. Mater. Interfaces 2018, 10, 18300. 29658698 10.1021/acsami.8b03268

[110] X. Wei , L. Wang , C. Duan , K. Chen , X. Li , X. Guo , P. Chen , H. Liu , Y. Fan , Bioact. Mater. 2023, 27, 271. 37122901 10.1016/j.bioactmat.2023.03.023 PMC10130885

[111] J. J. Norman , T. A. Desai , Ann. Biomed. Eng. 2006, 34, 89. 16525765 10.1007/s10439-005-9005-4

[112] J. del Barrio , C. Sánchez‐Somolinos , Adv. Opt. Mater. 2019, 7, 1900598.

[113] N. Qin , Z.‐G. Qian , C. Zhou , X.‐X. Xia , T. H. Tao , Nat. Commun. 2021, 12, 10.1038/s41467-021-25470-1. PMC8390743 34446721

[114] E. Bat , J. Lee , U. Y. Lau , H. D. Maynard , Nat. Commun. 2015, 6, 10.1038/ncomms7654. PMC4412366 25791943

[115] M. Leitgeb , D. Nees , S. Ruttloff , U. Palfinger , J. Götz , R. Liska , M. R. Belegratis , B. Stadlober , ACS Nano 2016, 10, 4926. 27023664 10.1021/acsnano.5b07411

[116] J. Ge , B. Ding , S. Hou , M. Luo , D. Nam , H. Duan , H. Gao , Y. C. Lam , H. Li , Nat. Commun. 2021, 12, 10.1038/s41467-021-23427-y. PMC8149427 34035283

[117] O. Dadras‐Toussi , M. Khorrami , A. S. C. Louis Sam Titus , S. Majd , C. Mohan , M. R. Abidian , Adv. Mater. 2022, 34, 2200512. 10.1002/adma.202200512 PMC9339506 35707927

[118] K. L. Choy , Prog. Mater. Sci. 2003, 48, 57.

[119] W. Liu , R. Shao , L. Guo , J. Man , C. Zhang , L. Li , H. Wang , B. Wang , L. Guo , S. Ma , Adv. Sci. 2024, 11, 2304046. 10.1002/advs.202304046 PMC11005734 38311581

[120] a) G. Ouyang , G. Yang , G. Zhou , Nanoscale 2012, 4, 2748; 22422101 10.1039/c2nr30095h

[121] a) D. Su , S. Dou , G. Wang , NPG Asia Mater. 2015, 7, 155; 10.1038/am.2015.114 PMC4861403 27175221

[122] M. N. Esfahani , B. E. Alaca , Mech. Mater. 2018, 127, 112.

[123] T. Hill , Nano Lett. 2001, 1.

[124] T. Gilanyi , J. Phys. Chem. B 1999, 103, 2085.

[125] P. Couchman , W. Jesser , Surf. Sci. 1973, 34, 212.

[126] H. Jiang , K.‐s. Moon , F. Hua , C. Wong , Chem. Mater. 2007, 19, 4482.

[127] S. Schofield , P. Studer , C. Hirjibehedin , N. Curson , G. Aeppli , D. Bowler , Nat. Commun. 2013, 4, 1649. 23552064 10.1038/ncomms2679 PMC3644071

[128] G. Ouyang , C. Wang , G. Yang , Chem. Rev. 2009, 109, 4221. 19670888 10.1021/cr900055f

[129] E. Gentleman , M. Gentleman , Int. Mater. Rev. 2014, 59, 417.

[130] M. Shaker , E. Salahinejad , Prog. Org. Coat. 2018, 119, 123.

[131] B. Molleman , Wageningen University and Research, 2019.

[132] F. D. Fischer , T. Waitz , D. Vollath , N. K. Simha , Prog. Mater. Sci. 2008, 53, 481.

[133] M. P. Finnegan , H. Zhang , J. F. Banfield , J. Phys. Chem. C 2007, 111, 1962.

[134] P. Pawlow , Z. Phys. Chem. 1909, 65, 1.

[135] Z. Wu , S. Yang , W. Wu , Nanoscale 2016, 8, 1237. 26696235 10.1039/c5nr07681a

[136] E. Ringe , R. P. Van Duyne , L. Marks , Nano Lett. 2011, 11, 3399. 21744799 10.1021/nl2018146

[137] F. Song , M. E. Brasch , H. Wang , J. H. Henderson , K. Sauer , D. Ren , ACS Appl. Mater. Interfaces 2017, 9, 22176. 28636823 10.1021/acsami.7b04757

[138] a) E. M. Harnett , J. Alderman , T. Wood , Colloids Surf. B Biointerfaces 2007, 55, 90; 17207976 10.1016/j.colsurfb.2006.11.021

[139] J. Wei , M. Yoshinari , S. Takemoto , M. Hattori , E. Kawada , B. Liu , Y. Oda , J. Biomed. Mater. Res. B Appl. Biomater. 2007, 81, 66. 16924616 10.1002/jbm.b.30638

[140] a) S.‐C. Luo , S. S. Liour , H.‐h. Yu , Chem. Commun. 2010, 46, 4731; 10.1039/c002321c 20490407

[141] a) L. Zeng , J. Gao , Y. Liu , J. Gao , L. Yao , X. Yang , X. Liu , B. He , L. Hu , J. Shi , M. Song , G. Qu , G. Jiang , TrAC, Trends Anal. Chem. 2019, 118, 303;

[142] V. Karde , C. Ghoroi , Int. J. Pharm. 2014, 475, 351. 25195729 10.1016/j.ijpharm.2014.09.002

[143] Y. Zhao , H. M. Wong , S. C. Lui , E. Y. Chong , G. Wu , X. Zhao , C. Wang , H. Pan , K. M. Cheung , S. Wu , ACS Appl. Mater. Interfaces 2016, 8, 3901. 26796319 10.1021/acsami.5b10881

[144] P. Zheng , Y. Ami'erjiang , B. Liu , M. F. Wang , H. Ding , B. B. Ding , J. Lin , Angew. Chem., Int. Ed. 2024, 63. 10.1002/anie.202317218 38212251

[145] a) J. P. Jeon , Y. J. Kim , S. H. Joo , H. J. Noh , S. K. Kwak , J. B. Baek , Angew. Chem., Int. Ed. 2023, 62; 10.1002/anie.202217416 36545845

[146] S. H. Ma , X. Luo , G. Ran , Y. P. Li , Z. Q. Cao , X. Y. Liu , G. Q. Chen , J. H. Yan , L. Wang , Chem. Eng. J. 2022, 435, 134810.

[147] M. Y. Chen , Y. Z. Sun , H. F. Ji , M. Jiang , W. D. Liu , M. Z. Shao , Z. Hao , H. Y. Zhang , X. Y. Li , Y. F. Dang , R. Z. Zhang , L. B. Zhang , Chem. Eng. J. 2023, 478, 147397.

[148] L. Wang , D. L. Chen , S. Q. Miao , F. Chen , C. F. Guo , P. C. Ye , J. Q. Ning , Y. J. Zhong , Y. Hu , Chem. Eng. J. 2022, 434, 133867.

[149] a) I. Z. Gutierrez , C. Gerke , Y. L. Shen , E. Ximendes , M. M. Silvan , R. Marin , D. Jaque , O. G. Calderón , S. Melle , J. Rubio‐Retama , ACS Appl. Mater. Interfaces 2022, 14, 4871; 35049282 10.1021/acsami.1c19344 PMC8815038

[150] X. F. Hu , X. Li , H. M. Yang , C. J. Xu , W. Q. Xiong , X. Guo , C. S. Xie , D. W. Zeng , ACS Sensors 2022, 7, 1894. 35734877 10.1021/acssensors.2c00487

[151] J. Y. Liu , H. Tang , P. M. Jian , B. Liu , Appl. Catal. B‐Environ. 2023, 334, 122828.

[152] a) X. Y. Gao , S. Vaidya , S. Dikshit , P. Ju , K. H. Shen , Y. B. Jin , S. X. Zhang , T. C. Li , Nat. Commun. 2024, 15, 7697; 39227570 10.1038/s41467-024-51941-2 PMC11372065

[153] A. Bhattacharjee , B. Pereira , P. Soares , K. C. Popat , Nanoscale 2024, 16, 12510. 38874593 10.1039/d4nr01123f PMC11223589

[154] J. M. Park , P. Asghari‐Rad , A. Zargaran , J. W. Bae , J. Moon , H. Kwon , J. Choe , S. S. Yang , J. H. Yu , H. S. Kim , Acta Mater. 2021, 221, 117426.

[155] W. S. Xia , X. B. Zhao , Q. Z. Yue , L. Yue , J. W. Wang , Q. Q. Ding , H. B. Bei , Z. Zhang , Acta Mater. 2021, 206, 116653.

[156] H. L. Deng , Y. B. Chen , Q. Zhu , Q. K. Zhao , Q. S. Huang , J. W. Wang , H. F. Zhou , Nano Lett. 2024, 24, 2511. 38373158 10.1021/acs.nanolett.3c04439

[157] X. H. Li , Y. Y. Zhang , L. Zhang , S. H. Xia , Y. Zhao , J. H. Yan , J. Y. Yu , B. Ding , Small 2022, 18, 2106500. 10.1002/smll.202106500 35199487

[158] S. Mandal , A. Kishore , S. Mandal , B. Bhar , B. B. Mandal , S. K. Nandi , M. Roy , Acta Biomater. 2023, 168, 650. 37451660 10.1016/j.actbio.2023.07.004

[159] J. E. Park , G. M. Na , K. Yeom , S. Park , H. J. Sim , Y. E. Sung , C. Choi , Chem. Eng. J. 2023, 459, 141671.

[160] P. Liu , H. Y. Zhang , Y. P. Han , J. Q. Zhao , Y. C. Zhang , Chem. Eng. J. 2023, 476, 146820.

[161] D. Y. Kim , J. H. Kim , M. C. Li , S. Noda , J. Kim , K. S. Kim , K. S. Kim , C. M. Yang , Appl. Surf. Sci. 2021, 566, 150751.

[162] W. B. Jian , W. L. Zhang , X. E. Wei , B. C. Wu , W. L. Liang , Y. Wu , J. Yin , K. Lu , Y. A. Chen , H. N. Alshareef , X. Q. Qiu , Adv. Funct. Mater. 2022, 32.

[163] J. C. Liu , D. J. Xu , G. C. Xu , X. A. Li , J. T. Dong , X. K. Luan , X. Z. Du , Chem. Eng. J. 2023, 475, 146312.

[164] L. H. T. Nguyen , Y. T. Dang , T. T. T. Nguyen , B. Q. G. Le , N. X. D. Mai , H. V. Nguyen , M. T. Le , T. B. Phan , T. L. Doan , New J. Chem. 2022, 46, 6630.

[165] X. L. Su , Y. L. Zhai , C. Jia , Z. Xu , D. F. Luo , Z. Y. Pan , H. X. Xiang , S. L. Yu , L. P. Zhu , M. F. Zhu , ACS Appl. Mater. Interfaces 2023, 15, 42920. 37650731 10.1021/acsami.3c06791

[166] L. Sutrisno , H. J. Chen , Y. Z. Chen , T. Yoshitomi , N. Kawazoe , Y. N. Yang , G. P. Chen , Biomaterials 2021, 275, 120923. 34098151 10.1016/j.biomaterials.2021.120923

[167] R. Ghafari , M. Jonoobi , F. Naijian , A. Ashori , T. H. Mekonnen , A. R. Taheri , Int. J. Biol. Macromol. 2022, 223, 100. 36347362 10.1016/j.ijbiomac.2022.10.281

[168] K. Xu , C. Lai , Y. X. Yang , H. Zhou , C. W. Zhou , Y. Yang , T. Yu , C. L. Yuan , Sensors Actuators B Chem. 2021, 329, 129095.

[169] H. X. Deng , B. J. Fang , Q. R. Zhang , X. Xiao , W. Yang , Mater. Lett. 2022, 328, 133169.

[170] H. Fan , H. Wang , M. Peng , H. Meng , A. Mundstock , A. Knebel , J. Caro , ACS Nano 2023, 17, 7584. 37026681 10.1021/acsnano.2c12774 PMC10134499

[171] J. M. Li , Y. M. Kang , W. L. Wei , X. Li , Z. Q. Lei , P. Liu , Chem. Eng. J. 2021, 407, 127961.

[172] K. P. Cao , W. Chen , D. Fan , Z. H. Jia , N. Chen , D. L. Zhu , S. T. Xu , A. M. Zheng , P. Tian , Z. M. Liu , Chem. Eng. J. 2024, 487, 150344.

[173] T. N. Vu , V. Pham , T. H. Fang , Mater. Today Commun. 2022, 33, 104282. 10.1016/j.mtcomm.2022.104288 PMC9394096 36033158

[174] M. D. Nguyen , L. Z. Deng , J. M. Lee , K. M. Resendez , M. Fuller , S. Hoijang , F. Robles‐Hernandez , C. W. Chu , D. Litvinov , V. G. Hadjiev , S. J. Xu , M. H. Phan , T. R. Lee , Small 2024. 10.1002/smll.202402940 39004867

[175] D. Zhou , G. H. Zhang , Y. Li , S. Liu , S. B. Han , Y. Zhou , W. J. Shen , Chem. Eng. J. 2023, 472, 144875.

[176] F. Saleem , G. Y. Liu , G. G. Liu , B. Chen , Q. B. Yun , Y. Y. Ge , A. Zhang , X. X. Wang , X. C. Zhou , G. Wang , L. W. Liao , Z. He , L. J. Li , H. Zhang , Small Methods 2024. 10.1002/smtd.202400430 PMC11579570 38970552

[177] G. Manohar , K. M. Pandey , S. R. Maity , Mater. Chem. Phys. 2022, 282, 126000.

[178] C. S. Zhang , Q. Hu , H. L. Fang , Z. X. Peng , J. Liu , J. Mater. Res. Technol. 2024, 31, 2531.

[179] H. J. Woo , S. J. Chung , M. L. Hill , K. Hadrick , T. Kim , ACS Appl. Nano Mater. 2023, 6, 9884. 38572409 10.1021/acsanm.3c01547 PMC10985654

[180] S. D. Liu , H. L. Liu , G. F. Zhou , X. G. Li , S. R. Wang , Chem. Eng. J. 2022, 427, 131430.

[181] J. Guo , Y. H. Fu , M. Lu , X. Y. Zhang , S. V. Kershaw , J. Zhang , S. L. Luo , Y. X. Li , W. W. Yu , A. L. Rogach , L. J. Zhang , X. Bai , Adv. Sci. 2020, 7, 2000930. 10.1002/advs.202000930 PMC7404144 32775167

[182] Z. C. Cao , H. M. Wang , J. L. Chen , Y. A. Zhang , Q. Y. Mo , P. Zhang , M. Y. Wang , H. Y. Liu , X. Y. Bao , Y. Z. Sun , W. Zhang , Q. Q. Yao , Bioact. Mater. 2023, 20, 221. 35702612 10.1016/j.bioactmat.2022.05.025 PMC9163388

[183] K. Xu , C. Mu , C. Zhang , S. Deng , S. Lin , L. Zheng , W. Chen , Q. Zhang , Biomaterials 2023, 301, 122268. 37572468 10.1016/j.biomaterials.2023.122268

[184] Y. Xiong , L. Chen , P. Liu , T. Yu , C. C. Lin , C. C. Yan , Y. Q. Hu , W. Zhou , Y. Sun , A. C. Panayi , F. Q. Cao , H. Xue , L. C. Hu , Z. Lin , X. D. Xie , X. F. Xiao , Q. Feng , B. B. Mi , G. H. Liu , Small 2022, 18, 2104229. 10.1002/smll.202104229 34791802

[185] X. Z. Dong , P. Wu , L. Yan , K. Liu , W. Y. Wei , Q. Cheng , X. Y. Liang , Y. Chen , H. L. Dai , Biomaterials 2022, 280, 121288. 34894585 10.1016/j.biomaterials.2021.121288

[186] R. Sheng , J. Mu , R. V. Chernozem , Y. R. Mukhortova , M. A. Surmeneva , I. O. Pariy , T. Ludwig , S. Mathur , C. Xu , R. A. Surmenev , H. H. Liu , ACS Appl. Mater. Interfaces 2023, 15, 3731. 36626669 10.1021/acsami.2c15802

[187] Y. N. Hu , Z. Y. Chen , H. Y. Wang , J. H. Guo , J. Y. Cai , X. Y. Chen , H. Wei , J. Y. Qi , Q. J. Wang , H. S. Liu , Y. J. Zhao , R. J. Chai , ACS Nano 2022, 16, 1868. 35112853 10.1021/acsnano.1c11627

[188] R. Shelat , L. K. Bhatt , B. Paunipagar , T. Kurian , A. Khanna , S. Chandra , J. Tissue Eng. Regener. Med. 2020, 14, 1604. 10.1002/term.3120 32840054

[189] Y. Cai , C. X. Wu , Q. H. Ou , M. H. Zeng , S. Xue , J. L. Chen , Y. Lu , C. H. Ding , Bioact. Mater. 2023, 19, 444. 35574050 10.1016/j.bioactmat.2022.04.021 PMC9079106

[190] C. C. Barrera‐Ortega , L. Hoz‐Rodríguez , H. Arzate , A. Fonseca‐García , J. Pérez‐Alvarez , S. E. Rodil , Mater. Sci. Eng., C 2017, 76, 1075. 10.1016/j.msec.2017.03.213 28482471

[191] A. L. Rosa , R. B. Kato , L. M. Castro Raucci , L. N. Teixeira , F. S. de Oliveira , L. S. Bellesini , P. T. de Oliveira , M. Q. Hassan , M. M. Beloti , J. Cell. Biochem. 2014, 115, 540. 24122940 10.1002/jcb.24688

[192] D. Cui , N. Kong , L. Ding , Y. Guo , W. Yang , F. Yan , Adv. Healthcare Mater. 2021, 10, 2101215. 10.1002/adhm.202101215 PMC11468541 34586717

[193] Z. Li , S. Q. Xiang , Z. X. Lin , E. N. Li , H. Yagi , L. Yocum , L. Li , K. K. Bruce , M. R. Fritch , H. L. Hu , B. Wang , P. G. Alexander , K. A. Khor , R. S. Tuan , H. Lin , Biomaterials 2021, 277, 121082. 34464823 10.1016/j.biomaterials.2021.121082 PMC8478843

[194] a) J. Qiu , H. Geng , D. Wang , S. Qian , H. Zhu , Y. Qiao , W. Qian , X. Liu , ACS Appl. Mater. Interfaces 2017, 9, 12253; 28345852 10.1021/acsami.7b00314

[195] K. H. Tan , S. Sattari , S. Beyranvand , A. Faghani , K. Ludwig , K. Schwibbert , C. Böttcher , R. Haag , M. Adeli , Langmuir 2019, 35, 4736. 30840824 10.1021/acs.langmuir.8b03660

[196] W. Liu , N. N. Zhao , Q. Yin , X. Y. Zhao , K. L. Guo , Y. F. Xian , S. W. Li , C. L. Wang , M. M. Zhu , Y. R. Du , F. J. Xu , C. Y. Wang , J. Zhou , ACS Nano 2023.

[197] P. Lazar , E. Otyepková , M. Pykal , K. Čépe , M. Otyepka , Nanoscale 2018, 10, 8979. 29693674 10.1039/c8nr00329g PMC5958343

[198] X. Liu , L. Li , B. Gaihre , S. Park , Y. Li , A. Terzic , B. D. Elder , L. Lu , ACS Nano 2022, 16, 2741. 35072461 10.1021/acsnano.1c09688 PMC9271266

[199] Y. Li , R. Z. Fu , Z. G. Duan , C. H. Zhu , D. D. Fan , Bioact. Mater. 2022, 9, 461. 34820583 10.1016/j.bioactmat.2021.07.023 PMC8586748

[200] S. Chen , A. F. U. H. Saeed , Q. Liu , Q. Jiang , H. Xu , G. G. Xiao , L. Rao , Y. Duo , Signal Transduction Targeted Ther. 2023, 8, 207. 10.1038/s41392-023-01452-1 PMC10200802 37211559

[201] F. Zhang , M. C. Lv , S. Y. Wang , M. Y. Li , Y. Wang , C. J. Hu , W. Hu , X. K. Wang , X. G. Wang , Z. D. Liu , Z. Fan , J. Z. Du , Y. Sun , Bioact. Mater. 2024, 31, 231. 37637084 10.1016/j.bioactmat.2023.08.008 PMC10450354

[202] M. Y. Hou , Y. S. Wei , Z. Y. Zhao , W. Q. Han , R. X. Zhou , Y. Zhou , Y. R. Zheng , L. C. Yin , Adv. Mater. 2022, 34, 2108817. 10.1002/adma.202108817 35044010

[203] Y. J. Yu , J. H. Yan , Q. W. Chen , J. Y. Qiao , S. Y. Peng , H. Cheng , M. W. Chen , X. Z. Zhang , J. Controlled Release 2023, 353, 591. 10.1016/j.jconrel.2022.12.014 36503071

[204] J. S. Suk , Q. Xu , N. Kim , J. Hanes , L. M. Ensign , Adv. Drug Delivery Rev. 2016, 99, 28. 10.1016/j.addr.2015.09.012 PMC4798869 26456916

[205] Z. G. Chen , Trends Mol. Med. 2010, 16, 594. 20846905 10.1016/j.molmed.2010.08.001 PMC3729441

[206] L. Rao , L. L. Bu , J. H. Xu , B. Cai , G. T. Yu , X. Yu , Z. He , Q. Huang , A. Li , S. S. Guo , W. F. Zhang , W. Liu , Z. J. Sun , H. Wang , T. H. Wang , X. Z. Zhao , Small 2015, 11, 6225. 26488923 10.1002/smll.201502388

[207] X. Ren , R. Zheng , X. Fang , X. Wang , X. Zhang , W. Yang , X. Sha , Biomaterials 2016, 92, 13. 27031929 10.1016/j.biomaterials.2016.03.026

[208] Y. Duan , J. Zhou , Z. Zhou , E. Zhang , Y. Yu , N. Krishnan , D. Silva‐Ayala , R. H. Fang , A. Griffiths , W. Gao , L. Zhang , Small 2023, 19, 2305551. 10.1002/smll.202305551 37635117

[209] D. E. 3rd Owens , N. A. Peppas , Int. J. Pharm. 2006, 307, 93. 16303268 10.1016/j.ijpharm.2005.10.010

[210] S. Peng , B. Ouyang , Y. Men , Y. Du , Y. Cao , R. Xie , Z. Pang , S. Shen , W. Yang , Biomaterials 2020, 231, 119680. 31864018 10.1016/j.biomaterials.2019.119680

[211] A. M. Wagner , D. S. Spencer , N. A. Peppas , J. Appl. Polym. Sci. 2018, 135, 46154. 30174339 10.1002/app.46154 PMC6114141

[212] S. Correa , E. C. Dreaden , L. Gu , P. T. Hammond , J. Control Release 2016, 240, 364. 26809005 10.1016/j.jconrel.2016.01.040 PMC6450096

[213] W. She , H. Li , Z. Wang , T. Liu , D. Zhao , Z. Guo , Y. Liu , Y. Liu , J. Controlled Release 2024, 366, 204. 10.1016/j.jconrel.2023.12.022 38109945

[214] a) M. F. Attia , N. Anton , J. Wallyn , Z. Omran , T. F. Vandamme , J. Pharm. Pharmacol. 2019, 71, 1185; 31049986 10.1111/jphp.13098

[215] Y. Song , Y. Du , C. Hu , L. Lei , L. Yang , X. Wang , C. Jiang , H. Gao , Adv. Funct. Mater., 2316145.

[216] R. Jin , G. Zhang , L. Tang , M. Li , M. Yang , J. Li , Z. Wang , F. Guan , ACS Appl. Nano Mater. 2024, 7, 3968.

[217] R. Davis , R. A. Urbanowski , A. K. Gaharwar , Curr. Opin. Biomed. Eng. 2021, 20, 100319. 35814330 10.1016/j.cobme.2021.100319 PMC9262334

[218] J. W. Yoo , D. J. Irvine , D. E. Discher , S. Mitragotri , Nat. Rev. Drug Discov. 2011, 10, 521. 21720407 10.1038/nrd3499

[219] Y. N. Ma , W. H. Gao , Y. J. Zhang , M. Yang , X. J. Yan , Y. Y. Zhang , G. Y. Li , C. Liu , C. L. Xu , M. Z. Zhang , ACS Appl. Mater. Interfaces 2022, 14, 6358. 35099925 10.1021/acsami.1c21700

[220] I. Ramirez‐Velez , A. A. Namjoshi , U. M. Effiong , N. A. Peppas , B. Belardi , ACS Nano 2024, 18, 21038. 39096293 10.1021/acsnano.4c02116 PMC12090900

[221] Z. C. Deng , W. Gao , F. Kohram , E. H. Li , T. V. Kalin , D. L. Shi , V. V. Kalinichenko , Bioact. Mater. 2024, 31, 1. 37593494 10.1016/j.bioactmat.2023.07.022 PMC10432146

[222] A. C. Anselmo , Y. Gokarn , S. Mitragotri , Nat. Rev. Drug Discov. 2019, 18, 19. 30498202 10.1038/nrd.2018.183

[223] Y. H. Zhou , Z. X. Chen , D. Zhao , D. Li , C. L. He , X. S. Chen , Adv. Mater. 2021, 33, 2102044. 10.1002/adma.202102044 34216408

[224] a) H. Cheng , Z. Shi , K. Yue , X. S. Huang , Y. C. Xu , C. H. Gao , Z. Q. Yao , Y. S. Zhang , J. Wang , Acta Biomater. 2021, 124, 219; 33556605 10.1016/j.actbio.2021.02.002

[225] M. J. Lin , J. Svensson‐Arvelund , G. S. Lubitz , A. Marabelle , I. Melero , B. D. Brown , J. D. Brody , Nat. Cancer 2022, 3, 911. 35999309 10.1038/s43018-022-00418-6

[226] Y. Li , Y. Luo , L. Hou , Z. Huang , Y. Wang , S. Zhou , Adv. Healthcare Mater. 2023, 12, 2202871. 10.1002/adhm.202202871 37276021

[227] A. K. Gaharwar , I. Singh , A. Khademhosseini , Nat. Rev. Mater. 2020, 5, 686.

[228] X. J. Zhou , Y. H. Qian , L. Chen , T. Li , X. Sun , X. J. Ma , J. W. Wang , C. L. He , ACS Nano 2023, 17, 5140. 36808939 10.1021/acsnano.3c00598

[229] J. C. Liang , H. J. Zeng , L. Qiao , H. Jiang , Q. Ye , Z. L. Wang , B. Liu , Z. J. Fan , ACS Appl. Mater. Interfaces 2022, 14, 30507. 35768948 10.1021/acsami.2c04168

[230] K. Feng , H. Y. Qiu , A. Gapeeva , X. Li , Y. Li , S. Kaps , Y. K. Mishra , R. Adelung , M. Baum , L. M. Yu , Prog. Org. Coat. 2023, 185, 107939.

[231] S. W. Yu , N. Sadaba , E. Sanchez‐Rexach , S. L. Hilburg , L. D. Pozzo , G. Altin‐Yavuzarslan , L. M. Liz‐Marzán , D. J. de Aberasturi , H. Sardon , Adv. Funct. Mater. 2024, 34. 10.1002/adfm.202311209 PMC11221775 38966003

[232] X. M. Xia , J. Meng , J. J. Qin , G. Z. Yang , P. Y. Xuan , Y. P. Huang , W. Fan , Y. Gu , F. L. Lai , T. X. Liu , ACS Appl. Polym. Mater 2024, 6, 3170.

[233] Y. Deng , F. Zhang , M. Jiang , Y. Liu , H. Yuan , J. Leng , ACS Appl. Mater. Interfaces 2022, 14, 42568. 36097702 10.1021/acsami.2c13982

[234] T. Kuhnt , S. Camarero‐Espinosa , M. Takhsha Ghahfarokhi , M. Arreguín , R. Cabassi , F. Albertini , D. Nieto , M. B. Baker , Adv. Funct. Mater. 2022, 32, 2202539.

[235] H. Ai , J. Anderson , K. Anseth , I. Antoniac , M. Barbosa , B. Basu , S. Best , R. Bettini , D. Bezuidenhout , R. Bizios , J. Brash , Y. Cao , J. Chang , G. Chen , E. Cosgriff‐Hernandez , A. Coury , J. Ding , X. Fu , A. García , B. Harley , J. Ji , K. Kataoka , J. Kohn , C. Laurencin , K. Leong , J.‐C. Lin , C. Liu , H. Lu , P. Ma , K. McLean , et al., in Definitions of Biomaterials for the Twenty‐First Century, (Eds: D. Williams , X. Zhang ), Elsevier, Cham 2019, IX.

[236] H. Zhu , M. Monavari , K. Zheng , T. Distler , L. L. Ouyang , S. Heid , Z. R. Jin , J. K. He , D. C. Li , A. R. Boccaccini , Small 2022, 18, 2104996. 10.1002/smll.202104996 35102718

[237] K. A. Gold , B. Saha , N. K. R. Pandian , B. K. Walther , J. A. Palma , J. Jo , J. P. Cooke , A. Jain , A. K. Gaharwar , Adv. Healthcare Mater. 2021, 10, 2101141. 10.1002/adhm.202101141 PMC9295047 34310082

[238] A. M. Duraj‐Thatte , A. Manjula‐Basavanna , J. Rutledge , J. Xia , S. Hassan , A. Sourlis , A. G. Rubio , A. Lesha , M. Zenkl , A. Kan , D. A. Weitz , Y. S. Zhang , N. S. Joshi , Nat. Commun. 2021, 12, 6600. 34815411 10.1038/s41467-021-26791-x PMC8611031

[239] P. Sharma , S. Brown , G. Walter , S. Santra , B. Moudgil , Adv. Colloid Interface Sci. 2006, 123–126, 471. 10.1016/j.cis.2006.05.026 16890182

[240] J. Li , F. Cheng , H. Huang , L. Li , J.‐J. Zhu , Chem. Soc. Rev. 2015, 44, 7855. 26214317 10.1039/c4cs00476k

[241] Y.‐P. Wang , X.‐H. Duan , Y.‐H. Huang , Y.‐J. Hou , K. Wu , F. Zhang , M. Pan , J. Shen , C.‐Y. Su , ACS Appl. Mater. Interfaces 2023, 15, 43479. 37694454 10.1021/acsami.3c08503

[242] B. Li , W. Wang , L. Zhao , D. Y. Yan , X. X. Li , Q. X. Gao , J. D. Zheng , S. T. Zhou , S. S. Lai , Y. Feng , J. Zhang , H. Jiang , C. M. Long , W. J. Gan , X. D. Chen , D. Wang , B. Z. Tang , Y. H. Liao , ACS Nano 2023, 17, 4601. 36826229 10.1021/acsnano.2c10694

[243] J. Tian , Y. Luo , L. Huang , Y. Feng , H. Ju , B. Y. Yu , Biosens. Bioelectron. 2016, 80, 519. 26890827 10.1016/j.bios.2016.02.018

[244] X. Yin , B. Yang , B. Chen , M. He , B. Hu , Anal. Chem. 2019, 91, 10596. 31311267 10.1021/acs.analchem.9b01721

[245] N. Liu , C. Homann , S. Morfin , M. S. Kesanakurti , N. D. Calvert , A. J. Shuhendler , T. Al , E. Hemmer , Nanoscale 2023, 15, 19546. 37982139 10.1039/d3nr05380f

[246] F. Jiang , B. Ding , S. Liang , Y. Zhao , Z. Cheng , B. Xing , P. a. Ma , J. Lin , Biomaterials 2021, 268, 120545. 33253965 10.1016/j.biomaterials.2020.120545

[247] T. Wang , Y. Chen , B. Wang , M. Wu , Front. Physiol. 2023, 14, 1126805. 36895633 10.3389/fphys.2023.1126805 PMC9990761

[248] T. Zhang , B. Wang , Q. Cheng , Q. Wang , Q. Zhou , L. Li , S. Qu , H. Sun , C. Deng , Z. Tang , Sci. Adv. 2024, 10, adn7896. 10.1126/sciadv.adn7896 PMC11225785 38968361

[249] J. Yao , J. Lifante , P. Rodríguez‐Sevilla , M. de la Fuente‐Fernández , F. Sanz‐Rodríguez , D. H. Ortgies , O. G. Calderon , S. Melle , E. Ximendes , D. Jaque , Small 2021, 17, 2103505. 10.1002/smll.202103505 34554636

[250] A. Deep , U. Tiwari , P. Kumar , V. Mishra , S. C. Jain , N. Singh , P. Kapur , L. M. Bharadwaj , Biosens. Bioelectron. 2012, 33, 190. 22284544 10.1016/j.bios.2011.12.051

[251] J. Luo , S. Jiang , H. Zhang , J. Jiang , X. Liu , Anal. Chim. Acta 2012, 709, 47. 22122930 10.1016/j.aca.2011.10.025

[252] B. A. Kairdolf , X. Qian , S. Nie , Anal. Chem. 2017, 89, 1015. 28043119 10.1021/acs.analchem.6b04873

[253] a) S. K. Mahobiya , S. Balayan , N. Chauhan , W. Rosario , N. K. Kuchhal , S. Islam , U. Jain , Appl. Surf. Sci. Adv. 2023, 16, 100425;

[254] B. C. Park , J. O. Soh , H.‐J. Choi , H. S. Park , S. M. Lee , H. E. Fu , M. S. Kim , M. J. Ko , T. M. Koo , J.‐Y. Lee , Y. K. Kim , J. H. Lee , ACS Nano 2024, 18, 12781. 38733343 10.1021/acsnano.3c12266

[255] Y. Wu , R. D. Tilley , J. J. Gooding , J. Am. Chem. Soc. 2019, 141, 1162. 30463401 10.1021/jacs.8b09397

[256] Z. Li , J. Lu , W. Wei , M. Tao , Z. Wang , Z. Dai , Chem. Commun. 2022, 58, 12418. 10.1039/d2cc04298c 36281644

[257] Y. Qin , J. Zhang , R. Tan , Z. Wu , M. Liu , J. Li , M. Xu , W. Gu , C. Zhu , L. Hu , ACS Sens. 2023, 8, 3257. 37566793 10.1021/acssensors.3c01269

[258] Y. Chen , Y. Qin , M. Liu , W. Yang , Y. Qiu , W. Li , L. Zheng , W. Gu , C. Zhu , L. Hu , Nat. Commun. 2025, 16, 2960. 40140374 10.1038/s41467-025-58174-x PMC11947233

[259] J. Park , C. Ban , Sci. Rep. 2023, 13, 10224. 37353600 10.1038/s41598-023-37418-0 PMC10290134

[260] X. Jiang , X. Zhang , C. Guo , B. Ma , Z. Liu , Y. Du , B. Wang , N. Li , X. Huang , L. Ou , Adv. Funct. Mater. 2024, 34, 2304426.

[261] T. Wang , Z. Wang , L. Bai , X. Zhang , J. Feng , C. Qian , Y. Wang , R. Wang , TrAC, Trends Anal. Chem. 2023, 168, 117328.

[262] M. A. Cook , G. D. Wright , Sci. Transl. Med. 2022, 14, abo7793. 10.1126/scitranslmed.abo7793 35947678

[263] Y. H. Luo , L. W. Chang , P. Lin , Biomed Res. Int. 2015, 2015, 143720. 26125021 10.1155/2015/143720 PMC4466342

[264] Y. L. Wang , Y. H. Lee , C. L. Chou , Y. S. Chang , W. C. Liu , H. W. Chiu , Environ. Pollut. 2024, 346, 123617. 38395133 10.1016/j.envpol.2024.123617

[265] I. U. Haq , K. Krukiewicz , Appl. Surf. Sci. Adv. 2023, 18, 100532.

[266] H. Bai , Z. Ding , J. Qian , M. Jiang , D. Yao , ACS Appl. Nano Mater. 2023, 6, 17531.

[267] K. Gwon , S. Lee , Y. Kim , J. Choi , S. Kim , S. J. Kim , H. J. Hong , Y. Hwang , M. Mori , D. N. Lee , Int. J. Biol. Macromol. 2023, 242, 124840. 37169053 10.1016/j.ijbiomac.2023.124840

[268] X. Wu , Z. Xing , H. Huang , Z. Ding , Y. Gao , M. Adeli , L. Ma , T. Ma , C. Cheng , C. Zhao , ACS Nano 2024, 18, 26168. 10.1021/acsnano.4c07406 39263719

[269] R. S. Li , J. Liu , C. Wen , Y. Shi , J. Ling , Q. Cao , L. Wang , H. Shi , C. Z. Huang , N. Li , Sci. Adv. 2023, 9, adg9601. 10.1126/sciadv.adg9601 PMC10456869 37624881

[270] M. Cloutier , D. Mantovani , F. Rosei , Trends Biotechnol. 2015, 33, 637. 26463723 10.1016/j.tibtech.2015.09.002

[271] Y. Zhang , Y. Chen , C. Lo , J. Zhuang , P. Angsantikul , Q. Zhang , X. Wei , Z. Zhou , M. Obonyo , R. H. Fang , W. Gao , L. Zhang , Angew. Chem., Int. Ed. 2019, 58, 11404. 10.1002/anie.201906280 31206942

[272] K. Quan , Y. Lu , Z. Mao , S. Wang , X. Ren , C. Yu , T. Zhang , J.‐J. Nie , Y. Cheng , D. Chen , Y. Zheng , D. Xia , Chem. Eng. J. 2024, 150147.

[273] Y. Xue , Z. Zhao , W. Huang , Z. Qiu , X. Li , Y. Zhao , C. Wang , R. Cui , S. Shen , H. Tian , L. Fang , R. Zhou , B. Zhu , J. Mater. Chem. B 2023, 11, 7750. 37475586 10.1039/d3tb01105d

[274] T. Chang , R. P. Babu , W. Zhao , C. M. Johnson , P. Hedström , I. Odnevall , C. Leygraf , ACS Appl. Mater. Interfaces 2021, 13, 49402. 34618446 10.1021/acsami.1c11236 PMC8532116

[275] N. Zandi , B. Dolatyar , R. Lotfi , Y. Shallageh , M. A. Shokrgozar , E. Tamjid , N. Annabi , A. Simchi , Acta Biomater. 2021, 124, 191. 33508511 10.1016/j.actbio.2021.01.029

[276] J. Park , J. Y. Kim , J. H. Heo , Y. Kim , S. A. Kim , K. Park , Y. T. Lee , Y. H. Jin , S. R. Shin , D. W. Kim , J. Seo , Adv. Sci. 2023, 10.

[277] Z. Li , C. Feng , W. Pang , C. Tian , Y. Zhao , ACS Nano 2021, 15, 9469. 33988023 10.1021/acsnano.1c02407

[278] C. Adiguzel , H. Karaboduk , F. G. Apaydin , S. Kalender , Y. Kalender , Toxicology Research 2023, 12, 741. 37915490 10.1093/toxres/tfad062 PMC10615818

[279] H. L. Karlsson , J. Gustafsson , P. Cronholm , L. Möller , Toxicol. Lett. 2009, 188, 112. 19446243 10.1016/j.toxlet.2009.03.014

[280] B. Singh , R. Bahadur , D. Rai , R. Srivastava , Advanced Therapeutics 2024, 7, 2300268.

[281] M. Sanavandi , K. Aalikhani , M. Shafiee , H. Rabbani , G. Fazli , N. Sadeghi , B. Shokri , Nano Lett. 2025, 25, 1974. 39868719 10.1021/acs.nanolett.4c05641

[282] Y. Li , K.‐C. Mei , R. Liam‐Or , J. T.‐W. Wang , F. N. Faruqu , S. Zhu , Y. Wang , Y. Lu , K. T. Al‐Jamal , ACS Nano 2024, 18, 22572. 39110092 10.1021/acsnano.4c08561 PMC11342366

[283] G. Scala , M. N. Delaval , S. P. Mukherjee , A. Federico , T. O. Khaliullin , N. Yanamala , L. M. Fatkhutdinova , E. R. Kisin , D. Greco , B. Fadeel , A. A. Shvedova , Carbon 2021, 178, 563. 37206955 10.1016/j.carbon.2021.03.045 PMC10193301

[284] J. R. Shaw , N. Caprio , N. Truong , M. Weldemariam , A. Tran , N. Pilli , S. Pandey , J. W. Jones , M. A. Kane , R. M. Pearson , Nat. Commun. 2025, 16, 10.1038/s41467-025-56210-4. PMC11754911 39843415

[285] L. Zhang , S. Chen , R. Ma , L. Zhu , T. Yan , G. Alimu , Z. Du , N. Alifu , X. Zhang , ACS Appl. Nano Mater. 2021, 4, 13060.

[286] G. Ultav , H. Tonbul , E. Salva , Journal of Drug Delivery Science and Technology 2022, 76, 103828.

[287] Y. Li , P. Hu , X. Wang , X. Hou , F. Liu , X. Jiang , Regenerative Biomaterials 2021, 8, rbab046. 34457350 10.1093/rb/rbab046 PMC8387661

[288] Q. Xia , Y. Zhang , Z. Li , X. Hou , N. Feng , Acta Pharm. Sin. B 2019, 9, 675. 31384529 10.1016/j.apsb.2019.01.011 PMC6663920

[289] J. Zhang , Y. Sun , L. Ren , L. Chen , L. Nie , A. Shavandi , K. E. Yunusov , U. E. Aharodnikau , S. O. Solomevich , G. Jiang , ACS Biomater. Sci. Eng. 2023, 10, 442. 38047725 10.1021/acsbiomaterials.3c01239

[290] I. Ferreira‐Faria , S. Yousefiasl , A. Macário‐Soares , M. Pereira‐Silva , D. Peixoto , H. Zafar , F. Raza , H. Faneca , F. Veiga , M. R. Hamblin , F. R. Tay , J. Gao , E. Sharifi , P. Makvandi , A. C. Paiva‐Santos , J. Controlled Release 2022, 351, 174. 10.1016/j.jconrel.2022.09.012 36103910

[291] J. Park , J. Y. Park , Y. Jeong , J. Park , Y. H. Park , S. Kim , D. Khang , Adv. Mater. 2023, 35, 2300934. 10.1002/adma.202300934 37114740

[292] N. Ren , N. Liang , M. Dong , Z. Feng , L. Meng , C. Sun , A. Wang , X. Yu , W. Wang , J. Xie , C. Liu , H. Liu , Small 2022, 18, 2202485. 10.1002/smll.202202485 35633288

[293] M. Kang , H. Y. Kim , S. H. Bhang , Journal of Industrial and Engineering Chemistry 2023, 119, 252.

[294] Y. Yin , N. Tian , Z. Deng , J. Wang , L. Kuang , Y. Tang , S. Zhu , Z. Dong , Z. Wang , X. Wu , M. Han , X. Hu , Y. Deng , T. Yin , Y. Wang , ACS Nano 2024, 18, 29089. 39393070 10.1021/acsnano.4c10509

[295] R. Q. Yao , C. Ren , Z. F. Xia , Y. M. Yao , Autophagy 2021, 17, 385. 32048886 10.1080/15548627.2020.1725377 PMC8007140

[296] a) S. Fulda , L. Galluzzi , G. Kroemer , Nat. Rev. Drug Discov. 2010, 9, 447; 20467424 10.1038/nrd3137

[297] C. Zelmer , L. P. Zweifel , L. E. Kapinos , I. Craciun , Z. P. Güven , C. G. Palivan , R. Y. H. Lim , Proc. Natl. Acad. Sci. USA 2020, 117, 2770. 31988132 10.1073/pnas.1916395117 PMC7022206

[298] P. Gao , W. Pan , N. Li , B. Tang , ACS Appl. Mater. Interfaces 2019, 11, 26529. 31136142 10.1021/acsami.9b01370

[299] A. L. Kersey , T.‐U. Nguyen , B. Nayak , I. Singh , A. K. Gaharwar , Mater. Today 2023, 64, 98.

[300] a) M. Taniguchi , S. Minami , C. Ono , R. Hamajima , A. Morimura , S. Hamaguchi , Y. Akeda , Y. Kanai , T. Kobayashi , W. Kamitani , Y. Terada , K. Suzuki , N. Hatori , Y. Yamagishi , N. Washizu , H. Takei , O. Sakamoto , N. Naono , K. Tatematsu , T. Washio , Y. Matsuura , K. Tomono , Nat. Commun. 2021, 12, 3726; 34140500 10.1038/s41467-021-24001-2 PMC8211865

[301] a) M. Jiang , P. Y. Wu , Y. W. Zhang , M. L. Wang , M. J. Zhang , Z. X. Ye , X. J. Zhang , C. Zhang , Adv. Healthcare Mater. 2024, 13;

[302] a) S. So , J. Rho , Nanophotonics 2019, 8, 1255;

[303] R. Menze , B. Hesse , M. Kusmierczuk , D. T. Chen , T. Weitkamp , S. Bettink , B. Scheller , Bioact. Mater. 2024, 32, 1. 37771679 10.1016/j.bioactmat.2023.09.008 PMC10522944

[304] G. G. Naik , V. A. Jagtap , Nano TransMed 2024, 3, 100041.

[305] F. P. Polack , S. J. Thomas , N. Kitchin , J. Absalon , A. Gurtman , S. Lockhart , J. L. Perez , G. P. Marc , E. D. Moreira , C. Zerbini , R. Bailey , K. A. Swanson , S. Roychoudhury , K. Koury , P. Li , W. V. Kalina , D. Cooper , R. W. Frenck , L. L. Hammitt , Ö. Türeci , H. Nell , A. Schaefer , S. Ünal , D. B. Tresnan , S. Mather , P. R. Dormitzer , U. Şahin , K. U. Jansen , W. C. Gruber , N. Engl. J. Med. 2020, 383, 2603. 33301246 10.1056/NEJMoa2034577 PMC7745181

[306] S. Sharifi , G. Caracciolo , D. Pozzi , L. Digiacomo , J. Swann , H. E. Daldrup‐Link , M. Mahmoudi , Adv. Drug Delivery Rev. 2021, 174, 337. 10.1016/j.addr.2021.04.028 33957181

